# Validation of the EmotiBit wearable sensor for heart-based measures under varying workload conditions

**DOI:** 10.3389/fnrgo.2025.1585469

**Published:** 2025-06-18

**Authors:** Anna Vorreuther, Nektaria Tagalidou, Mathias Vukelić

**Affiliations:** ^1^Applied Neurocognitive Systems, Institute of Human Factors and Technology Management, University of Stuttgart, Stuttgart, Germany; ^2^Applied Neurocognitive Systems, Fraunhofer Institute for Industrial Engineering, Stuttgart, Germany

**Keywords:** photoplethysmography, electrocardiography, electrodermal activity, heart rate, skin conductance, cognitive workload

## Abstract

**Introduction:**

The EmotiBit photoplethysmography (PPG) device allows user-owned data collection for measures of cardiovascular activity (CVA) and electrodermal activity (EDA) in naturalistic settings. The aim of this study was to evaluate the validity of this device for collecting high-quality data while participants experience varying levels of cognitive workload.

**Methods:**

Using a standardized criterion validity protocol, recordings of 15 participants performing a cognitive workload task were compared for the EmotiBit and a reference electrocardiography (ECG) device (BITalino PsychoBit). Multiple preprocessing pipelines and a signal quality check were implemented. Parameters of interest including heart rate (HR), heart rate variability (HRV) measures, skin conductance level (SCL), and skin conductance response (SCR) measures were assessed using Bland-Altman plot and ratio (BAr) analyses, as well as cross-correlations of the EDA signal time series of both devices.

**Results:**

BAr results indicated good agreement between devices regarding HR with an average difference of 1–2 beats per minute (bpm). HRV measures yielded an insufficient BAr, albeit most data points lay within a priori boundaries of agreement. EDA measures yielded insufficient agreement for comparing SCL and SCR number and amplitude.

**Discussion:**

The results are comparable to the validation of similar wearable PPG devices and extend the validation of the EmotiBit by assessing the acquired signals during varying levels of cognitive workload. While the device may be used to collect HR for scientific data analysis, its quality regarding HRV and EDA measures is not comparable to a standard ECG.

**Significance:**

This study provides the first systematic validation following a standardized protocol of the EmotiBit PPG device relative to an ECG when considering recordings collected during cognitive workload induction.

## 1 Introduction

The introduction of consumer-grade wearable sensors has extended physiological monitoring beyond clinical applications to everyday, occupational, and consumer settings. These devices offer the potential to assess cardiovascular activity (CVA), cognitive workload, and stress in real-time, providing valuable insights for domains such as human performance, neuroergonomics, and adaptive work environments (Brandt-Rauf and Ayaz, 2024; Hogervorst et al., 2014). The most prominent way for mobile devices to assess physiological signals is photoplethysmography (PPG). This method non-invasively captures the blood volume changes in the microvascular tissue of the skin by detecting changes in light transmission from a light-emitting diode (LED) to a photodiode (Peláez and Villegas, 2007). PPG is well-suited for wearable devices as it is cost-effective in production and can be worn flexibly without gel or any other supporting material, unlike the electrodes utilized during electrocardiography (ECG). Although ECG is superior in detecting CVA and related phenomena and remains the standard in medical settings, PPG is considered sufficient for most consumer uses like measuring heart rate (HR), heart rate variability (HRV), or oxygen saturation (Huhn et al., 2022).

Wearable sensors are increasingly applied in neuroergonomics to unobtrusively monitor physiological correlates of mental effort, fatigue, and stress in naturalistic work environments. While some PPG wearables have medical certifications (e.g., Empatica series) and may thus be considered for clinical use cases, the data is not solely user-owned, and algorithms are usually not published. The importance of open-source PPG software and hardware is apparent for applications in science and a focus on data privacy. Open-source wearables like the EmotiBit attempt to bridge the gap between cost-effective consumer-grade affordability and high-quality research-grade data acquisition (e.g., Empatica series, BIOPAC series, BITalino series). Such devices enable customizable data pipelines, algorithmic transparency, and cost-effective large-scale data collection—critical aspects when monitoring cognitive states in real-world work environments (Langevin et al., 2021; van Lier et al., 2020).

The EmotiBit has already gained some popularity. Numerous studies utilized the EmotiBit in applied scenarios (Gao and Zhou, 2022; Lobosco, 2023; Morris et al., 2023; Olivaz and Kulgod, 2023; Pelc et al., 2023; Reyes-Consuelo et al., 2023; Rizzi et al., 2023). Guarducci et al. (2025) included the EmotiBit in a comprehensive technical review of wearables detailing software and hardware features. The developer team released a validation of the device utilizing simultaneous data collection with an ECG device (the Brain Products V-Amp) from participants watching videos in sitting and standing positions (Montgomery et al., 2024). A recently published study by Haratian (2024) focusing on utilizing body-sensing technologies to assess user experience and safety during human-robot interaction employed both Empatica and EmotiBit devices and compared the recorded HR and galvanic skin response (GSR) data. On a descriptive level focusing on mean values over time, they found that HR detection seemed similar in devices, whereas GSR recordings seemed to differ substantially with the EmotiBit not correctly capturing the changes. Prior validation work furthermore includes a study by Langevin et al. (2021). The authors validated the EmotiBit PPG data of CVA and electrodermal activity (EDA) with a reference ECG device during resting state recordings. They utilized a standardized validation protocol for physiological signals from wearable technology (van Lier et al., 2020). It focuses on signal comparison, employing cross-correlations and inspecting parameters of interest commonly derived from the physiological signals. While the EmotiBit has shown promise in resting-state evaluations of HR, this prior validation work revealed limitations in HRV and EDA measurement accuracy (Langevin et al., 2021). No studies to date have systematically validated the EmotiBit during cognitive workload induction against a reference device. Given the increasing use of wearable sensors in applied neuroergonomics and workload monitoring, assessing the EmotiBit under task-induced cognitive workload is essential to establish its reliability in applied contexts such as neuroergonomics.

This study aims to assess the validity of the EmotiBit wearable sensor for measuring cardiovascular activity (HR and HRV) and electrodermal activity (EDA) under task-induced cognitive workload, following the standardized protocol proposed by van Lier et al. (2020) and extending the validation studies of the Emotibit device (Langevin et al., 2021; Montgomery et al., 2024). We specifically evaluated the EmotiBit's PPG sensor performance compared to an ECG and EDA system (BITalino PsychoBit; Batista et al., 2017) across two workload levels, while positioning the sensor on the upper arm—a location more practical for field applications but potentially challenging for signal quality (Kasos et al., 2020).

## 2 Methods

### 2.1 Mobile multi-sensor wearable device

**Figure 1:**
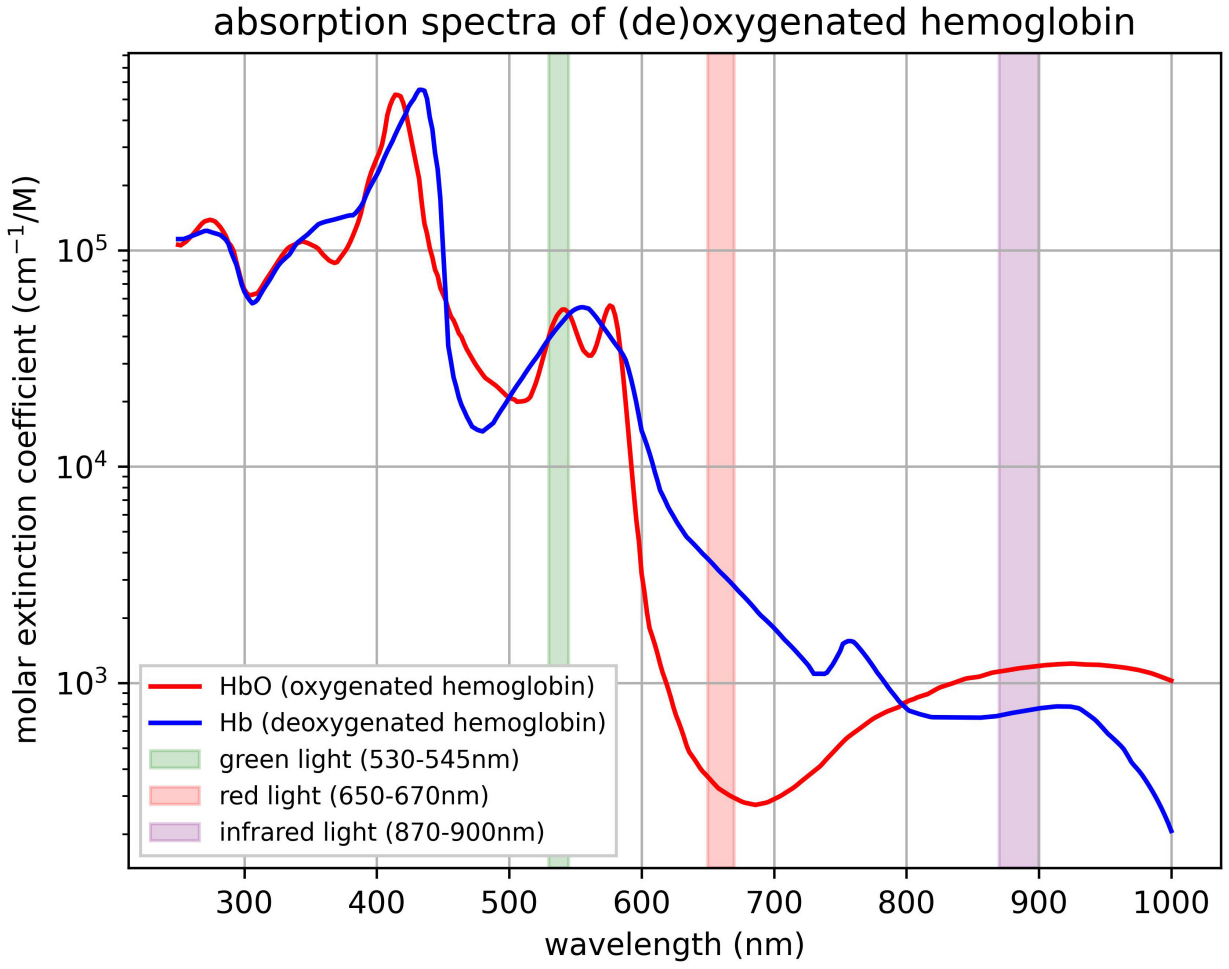
Light wavelengths of EmotiBit PPG sensor. The absorption spectra of oxygenated hemoglobin (HbO) and deoxygenated hemoglobin (Hb) represented by the molar extinction coefficient (**cm**^**−1**^**/M**) on a logarithmic scale is plotted against wavelength (nm) with highlighted bands corresponding to specific light wavelengths used in the MAX30101 sensor (values derived from SparkFun Photodetector (MAX30101) Hookup Guide - SparkFun Learn, 2025). The highlighted bands include the wavelengths measured of the green light spectrum (530–545 nm), the red light spectrum (650–670 nm), and the infrared light spectrum (870–900 nm) built into the sensor. Reproduced from Prahl (1999).

The EmotiBit (https://www.emotibit.com) is an open-source, mobile wearable capable of measuring physiological and movement data with 16 signals including PPG measurements, EDA, temperature, accelerometer, and gyroscope. We used the device built with the Arduino FeatherWing module (featherwing version “Adafruit Feather HUZZAH32,” hardware version “V05c”). The device can be attached at multiple different body locations through differently sized Velcro straps. All data are recorded on a built-in 32Gb-sized SD card and are thus 100% user-owned. Wireless data streaming options are also possible via a local network for live analysis and evaluation of signals' quality. The EmotiBit stores three distinct wavelengths as separate PPG signal streams. The wavelengths are those of green, red, and infrared light as measured by the MAX30101 sensor (see Figure 1). With an alternative open-source firmware version (“EmotiBit_stock_firmware_PPG_100Hz,” version 1.9.0; https://github.com/EmotiBit/EmotiBit_FeatherWing/releases), the sampling frequency of the PPG can be enhanced to 100 Hz as opposed to the default firmware with 25 Hz. The present study focuses exclusively on the PPG and EDA data, which were recorded at a sampling frequency of 100 Hz and 15 Hz, respectively. The recordings were performed with the corresponding oscilloscope and data parser software. Timestamps of experimental events and recorded data of the EmotiBit as well as the reference ECG device were synchronized using Lab Streaming Layer (LSL; Kothe et al., 2024; Kothe, 2025).

### 2.2 Reference device

The BITalino PsychoBit (BITalino [6.2] v5.2; https://www.pluxbiosignals.com/collections/bitalino/products/psychobit) is a data acquisition tool that is utilized in the measurement of various physiological parameters, including ECG, EDA, respiration, pulse rate, and light. In the present study, the BITalino PsychoBit was employed for the measurement of ECG and EDA. The ECG and EDA data were recorded at a sampling rate of 1,000 Hz using the corresponding recording software OpenSignals (version: 1.1; https://www.pluxbiosignals.com/pages/opensignals).

### 2.3 Participants

A total of 18 participants were recorded for this study (nine female, nine male; 48.67 ± 13.95 years old). They were recruited via the participants database of the research team. Three participants were excluded prior to analyses: for one participant, we experienced technical difficulties with the stimulus presentation; for two participants, the EmotiBit recording with LSL failed due to network issues. Thus, the data of 15 participants (46.87 ± 14.40 years; seven females, eight males) was used for analyses.

### 2.4 Experimental procedure

**Figure 2:**
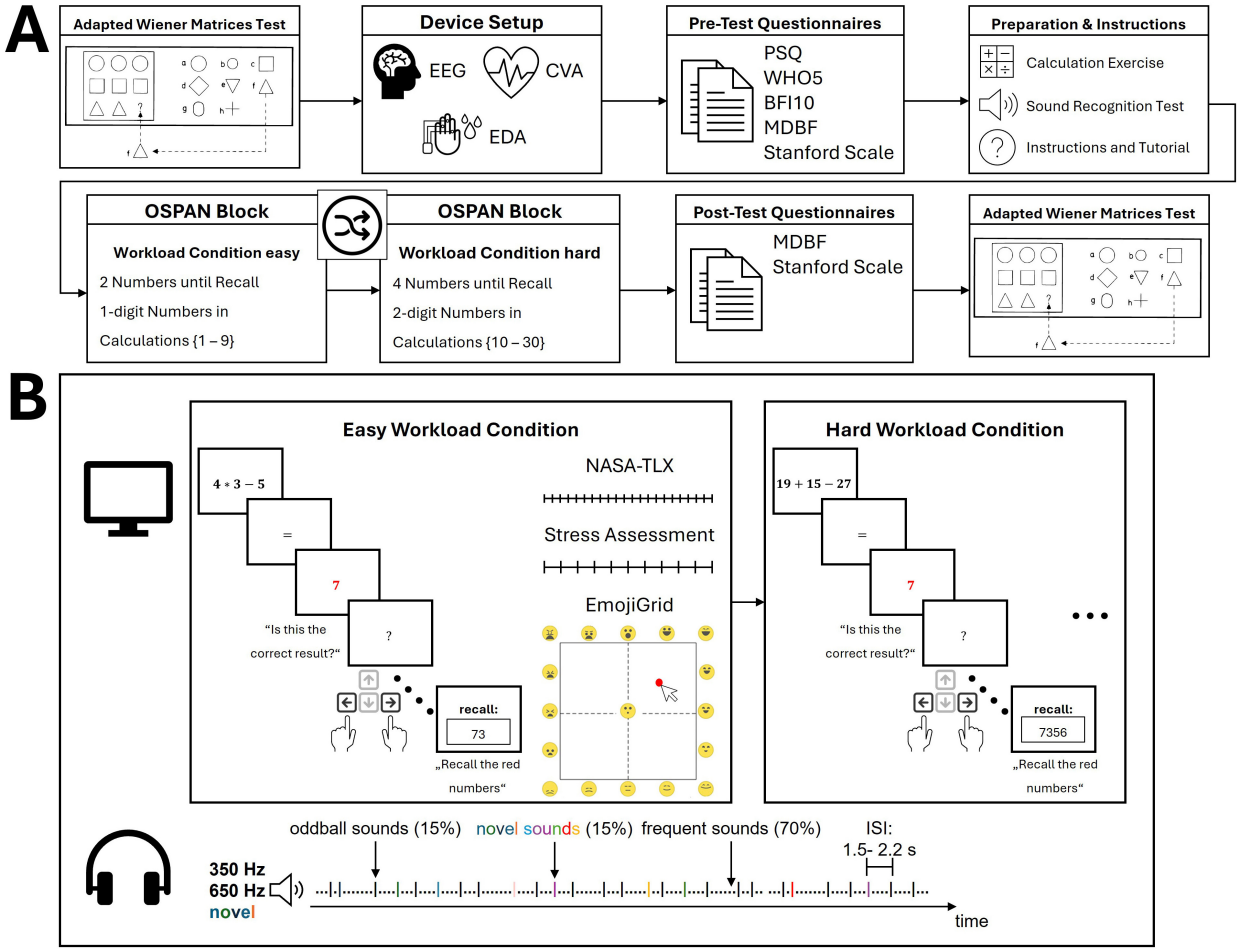
Experimental pocedure. **(A)** The timeline of the experimental procedure is illustrated with time estimates for each phase. The experimental block consisted of an operation span task with an easy and hard workload condition counterbalanced in order across participants. Note that the adapted Wiener Matrices Test had a fixed duration while the other time periods were dependent on the individual speed of completion. **(B)** The experimental blocks are illustrated for both workload conditions. During the visual OSPAN task, participants performed mental calculations and delayed recalls and responded with button presses (left or right) counterbalanced across participants. This was interjected with three types of questionnaires to assess current mental load, stress, and emotional state. An auditory implicit oddball task was presented and implicitly attended to with counterbalanced frequencies for oddball and frequent sounds and jittered inter-stimulus intervals. BFI10, Big Five Inventory-10 (Rammstedt et al., 2017); ISI, inter-stimulus interval; MDBF, German Multidimensional Mood State Questionnaire (Hinz et al., 2012); NASA-TLX, NASA Task Load Index (Hart and Staveland, 1988); OSPAN, operation span task; PSQ, Perceived Stress Questionnaire (Fliege et al., 2001); Stanford Scale, Stanford Sleepiness Scale (Hoddes et al., 1972); WHO5, WHO-5 WellBeing Index (Brähler et al., 2007).

Following the recruitment, participants signed informed consent and were informed about the study's procedure. The study was approved by the ethics commission of the University of Tübingen (Germany) and conducted according to the Declaration of Helsinki (502/2023BO2; the preregistered study design can be found at https://doi.org/10.17605/OSF.IO/AUVDR). Firstly, the study commenced with a pre-test in the form of an adapted Wiener Matrices Test (WMT; Formann, 1979). After completing the WMT, the participants were equipped with the sensors. For this study, only the CVA and EDA activity will be reported. However, additional research questions involved electroencephalography (EEG) data of a 64-channel gel-based system and a commercial PPG-Sensor (Fitbit Sense 2). After all the sensors were set up, the main experiment and physiological data acquisition commenced. Participants completed an operation span task in two difficulty levels counterbalanced across participants. The task consisted of a calculation component interjected with a delayed recall component (Daneman and Carpenter, 1980; Scharinger et al., 2017; Turner and Engle, 1989). The recall component involved the memorization of sequences of individual digits of varying lengths, whilst the calculation component necessitated the performance of calculations (addition, subtraction, multiplication) within each round. The dual nature of this task is known to impose significant demands on cognitive processing and, by extension, on cognitive workload (Scharinger et al., 2017). For inducing two distinct difficulty levels—easy and hard runs— the complexity of the calculations was varied (one-digit vs. two-digit numbers) and the length of sequenced numbers presented per recall phase (two vs. four numbers in a row). In addition, an auditory oddball task was presented throughout both conditions (Squires et al., 1975). The participants were not instructed to respond to the auditory task but rather to listen to the sounds passively as an additional measure of attentional resources. Finally, the participants completed the second half of the WMT as a post-session measurement (see Figure 2).

### 2.5 Sensor positioning

**Figure 3:**
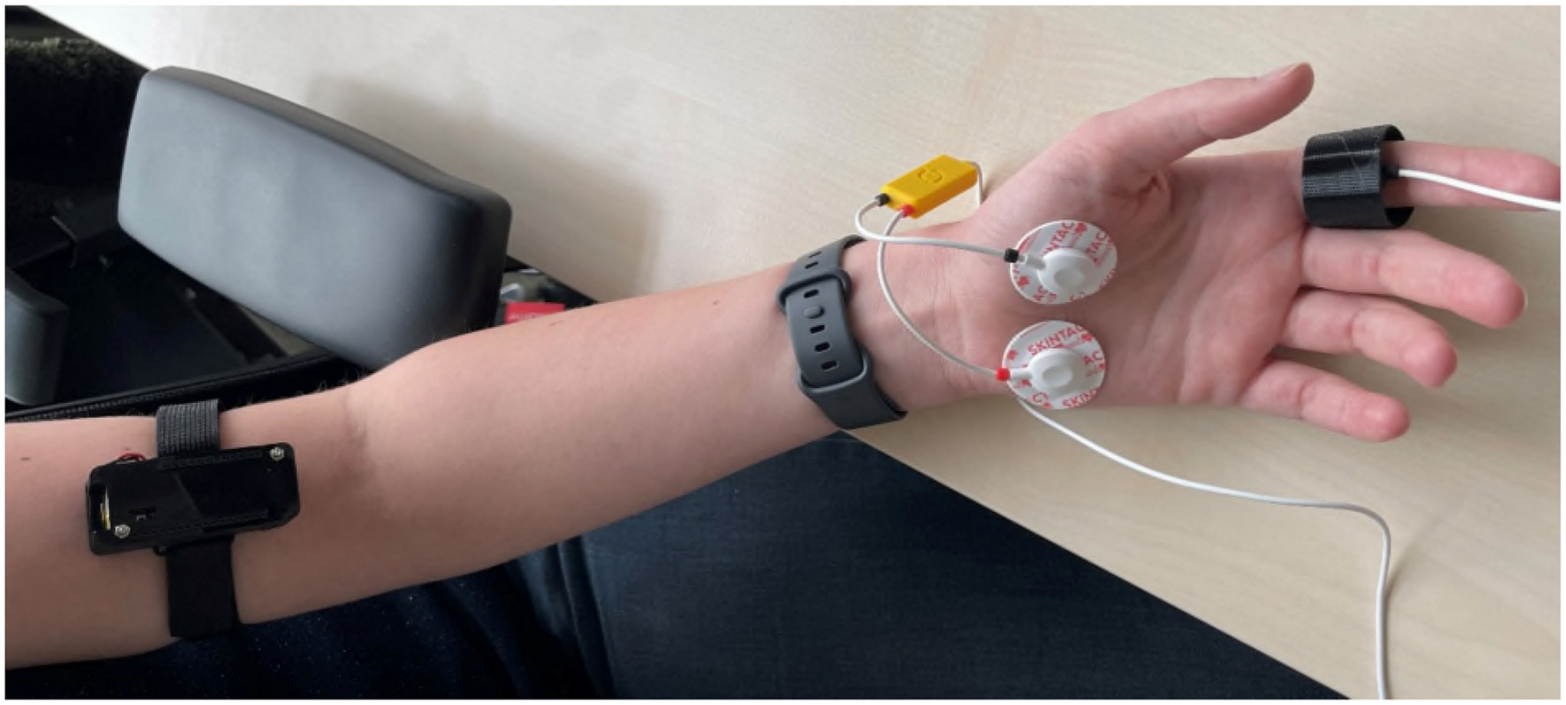
Picture of the sensor positioning at the arm. The EmotiBit device was placed on the upper arm and the PsychoBit EDA sensors were adhered to the left palm. Additionally, the PsychoBit pulse sensor was attached to the index finger and a consumer wearable (Fitbit Sense 2) was positioned above the wrist, albeit not reported in the present study.

The EmotiBit was positioned on the inner aspect of the left arm at the level of the biceps. The veins beneath the skin were identified and the EmotiBit was positioned above them for optimal signal detection (see Figure 3). This body location allows for comfortable placement without impeding natural movements. The PsychoBit ECG configuration comprised three electrodes: two positioned beneath the left and right clavicular bones and a reference electrode on the left elbow bone. The EDA sensors were adhered to the left palm (see Figure 3).

### 2.6 Data analysis

We compared both the EDA and CVA as measured by the EmotiBit and PsychoBit. For analysis, the python library *neurokit* (version 0.2.10; Makowski et al., 2021) was primarily used and multiple configurations of preprocessing steps were tested. The conditions of the experimental task were assessed separately.

#### 2.6.1 Preprocessing pipelines

**Figure 4:**
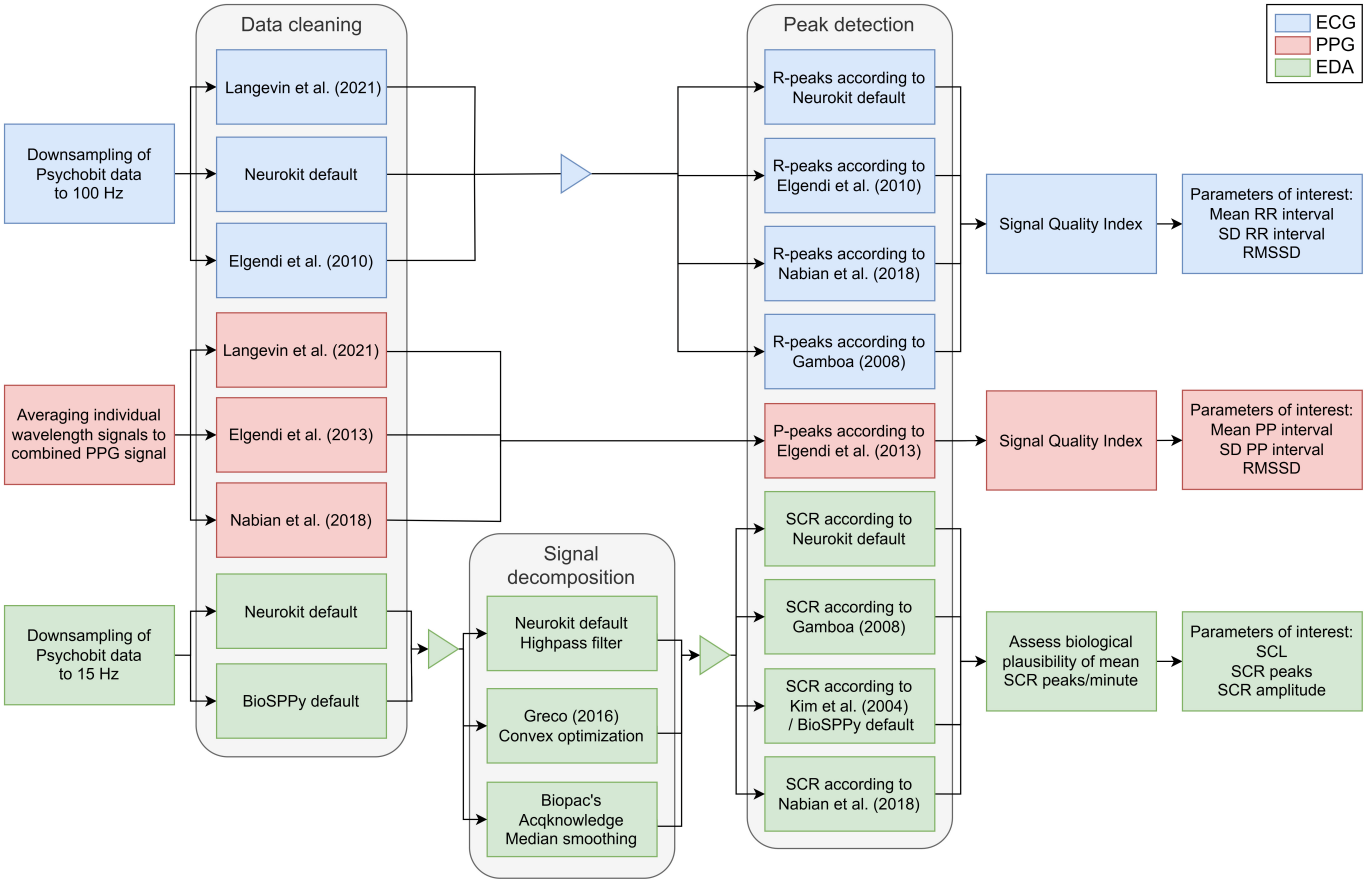
Processing pipelines for CVA and EDA signals. The processing pipelines are depicted for ECG (blue), PPG (red), and EDA (green) data. For data cleaning, signal decomposition of the EDA, and peak detection, various methods were tested. ECG, electrocardiography; EDA, electrodermal activity; PPG, photoplethysmography; RMSSD, root mean square difference of the successive differences; SCL, skin conductance level; SCR, skin conductance response; SD, standard deviation.

To assess the comparability of the measures between devices, we implemented several preprocessing pipelines derived from previous studies for both EDA and CVA signals (for details, see Figure 4; Table 1).

**Table 1:** Overview of processing configurations.

**Abbreviation**	**Processing**		
	**Data cleaning**	**Signal decomposition**	**Peak detection**
**ECG**			
Neurokit default	High-pass 5th-order Butterworth filter with cut-off frequency of 0.5 Hz, followed by powerline filtering at 50 Hz	n.a.	Method of Brammer (2020)^[1]^: QRS complexes are detected based on the steepness of the absolute gradient of the ECG signal and subsequently, R-peaks are detected as local maxima in the QRS complexes
Langevin et al. (2021)	Notch filter with a cut-off frequency of 0.05 Hz	n.a.	n.a.
Elgendi et al. (2010)	IIR Butterworth filter following the second-order-section method with cut-off frequencies of 8 Hz and 20 Hz	n.a.	Dual moving averages with dynamic thresholding
Nabian et al. (2018)	n.a.	n.a.	A sliding window approach identifies local maxima within a defined window size, marking points as R-peaks if they are the highest within their respective window
Gamboa (2008)	n.a.	n.a.	Zero crossings in the second-order derivative of the normalized signal are filtered for potential peaks based on amplitude and timing constraints, and R-peaks are selected by finding local maxima
**PPG**			
Langevin et al. (2021)	Band-pass filter with cut-off frequencies of 0.7 Hz and 3.5 Hz	n.a.	n.a.
Neurokit default/Elgendi et al. (2013)	3rd-order Butterworth filter with cut-off frequencies of 0.5 Hz and 8 Hz	n.a.	Dual moving averages with dynamic thresholding
Nabian et al. (2018)	2nd-order Butterworth filter with cut-off frequencies of 40 Hz	n.a.	n.a.
**EDA**			
Neurokit default	Low-pass filter with a cut-off frequency of 3 Hz and a 4th-order Butterworth filter	High-pass 4th-order Butterworth filter with a cut-off frequency of 0.05 Hz is applied to decompose the phasic and tonic signal components	Local maxima in the first-order derivative exceeding a dynamic threshold are identified and validated as peaks based on the expected SCR waveform shape
BioSPPy default	Low-pass filter with a cut-off frequency of 5 Hz and a 4th-order Butterworth filter	n.a.	same as Kim et al. (2004)^[5]^
Kim et al. (2004)	n.a.	n.a.	Convolution of the phasic component with a 20-point Bartlett window is performed and the output waveform is checked for the occurrence of the SCR by finding two consecutive zero-crossings, from negative to positive and positive to negative
Gamboa (2008)	n.a.	n.a.	Local maxima (peaks) and minima (onsets) are identified by computing the second derivative and to ensure proper peak-onset pairing and SCR amplitudes are calculated based on the difference between these points.
Nabian et al. (2018)	n.a.	n.a.	The amplitude of the SCR is obtained by finding the maximum value between these two zero-crossings, and calculating the difference between the initial zero crossing and the maximum value; detected SCRs with amplitudes smaller than 10% of the maximum SCR amplitudes that are already detected on the differentiated signal will be eliminated
Greco et al. (2016)	n.a.	A convex optimization approach grounded in Bayesian statistic models the components by fitting a Bateman autoregressive moving average (ARMA) model and applying spline and trend regressors	n.a.
Biopac's AcqKnowledge (BIOPAC Systems, Inc., 2015)	n.a.	A median smoothing convolutional filter with a window of 4 s is applied to extract the tonic component and subtract it from the original signal to obtain the phasic component	n.a.

Processing steps were derived from previous work by Brammer, 2020; Elgendi et al., 2010; Elgendi et al., 2013; Gamboa, 2008; Kim et al., 2004; Langevin et al., 2021; Nabian et al., 2018; Greco et al., 2016; BIOPAC Systems, Inc., 2015. ECG, electrocardiography; EDA, electrodermal activity; IIR, infinite impulse response; n.a., not applicable; PPG, photoplethysmography; SCR, skin conductance response.

##### 2.6.1.1 CVA

For the CVA signal, the PsychoBit ECG signal was downsampled to match the 100 Hz sampling frequency of the EmotiBit PPG and allow for accurate comparison. For comparative analysis with the ECG, we averaged the wavelengths to one PPG signal stream (Orphanidou et al., 2015). We followed various processing pipelines for the CVA data of both devices, as detailed in Figure 4 and Table 1. To ascertain the parameters of interest, the distances between two successive R-peaks from the QRS complexes (RR-interval) in the ECG signal were determined. Concurrently, the corresponding time intervals between the top of two peaks in the blood volume pulse signal (PP-interval) from the PPG signals were detected. The parameters of interest for subsequent comparative analysis were the following: (1) The mean RR/PP intervals which were converted to HR in beats per minute (bpm), (2) the standard deviation (SD) of RR/PP intervals, and (3) the root mean square of the successive differences (RMSSD) of RR/PP intervals, which is related to HR variability (Kim et al., 2018).

After preprocessing the signals were checked for quality by computing a signal quality index (SQI; Langevin et al., 2021; Orphanidou et al., 2015). The signals were epoched into 10 s time segments and sequentially checked for the biological plausibility of the measured signals. Each epoch was checked for the following rules: (1) The extrapolated HR is within the range of 30–180 bpm; (2) the maximum gap between two successive peaks is 3 s to ensure no more than one beat is missed; (3) within a 10 s epoch the HR is not expected to change more than 10% and thus the ratio of the maximum beat-to-beat interval to the minimum beat-to-beat interval within an epoch should be < 2.2.

Finally, the average correlation coefficient between each pulse peak within an epoch was calculated (Orphanidou et al., 2015). For each sample, the median beat-to-beat interval was calculated using all the detected pulse peaks. Individual pulse waves were extracted by taking a window of width equal to the median beat-to-beat interval centered on each peak. A template of the average pulse wave was obtained by taking the mean of all waves of the sample. The correlation coefficient of each individual pulse wave with the template was then calculated. The average correlation coefficient was obtained by averaging all correlation coefficients over the whole sample. If this coefficient was smaller than 0.66 for the ECG signal and 0.86 for the PPG signal (Langevin et al., 2021; Orphanidou et al., 2015) or the epoch did not pass the physiological rules, the epoch was labeled as “bad.” Epochs of sufficient quality were concatenated for computation of the parameters of interest, since the variance of especially HRV measures increases by short signal lengths (Fujita and Suzuki, 2019; Taoum et al., 2022). To allow for a balanced comparison of devices, the signals were trimmed to equal lengths based on the minimum number of good epochs per condition. That is, if the PPG signal retained fewer good epochs compared to the ECG signal, the ECG signal was cut to the length of the PPG signal.

##### 2.6.1.2 EDA

For the EDA signal, we first down sampled the PsychoBit signal to match the 15 Hz sampling frequency of the EmotiBit. For the following steps, different configurations of preprocessing steps were tested. The resampled data was initially low-pass filtered with either a 3 Hz (*neurokit* default) or 5 Hz (BioSPPy default; Carreiras et al., 2015) cut-off frequency and a 4th-order Butterworth filter to remove noise and smooth the signal. Subsequently, the EDA was decomposed into the phasic and tonic components of the signal by applying three different signal decomposition methods (for details, see Table 1). The following parameters of interest were retrieved: (1) The mean and standard deviation of the skin conductance level (SCL) was calculated by averaging the tonic component of the data over each run; (2) the number of skin conductance responses (SCRs) per minute, and (3) their amplitudes as derived from the phasic signal component. The implemented approaches are detailed in Table 1.

Biologically plausible values for the number of SCRs are on average 1–3 per minute during resting state (Braithwaite et al., 2013) and 20–25 per minute during high-arousal states (Boucsein et al., 2012). Since the initial inspection of results revealed implausibly high SCR detection for some processing pipelines, a quality check was implemented based on the average SCRs per minute detected in the reference device. If the average across participants was higher than 30 responses per minute, an overestimation of SCR was assumed, and the pipeline was excluded from further analysis.

#### 2.6.2 Signal comparison of EDA: cross-correlations

Data of both devices was processed using multiple different processing pipelines (for details, see Section 2.6.1). For an initial assessment of similarity, the cross-correlation between the z-scored, detrended signals of the two devices was determined for time lags from −8 to +8 samples, which corresponds to a time window of −0.53 s and 0.53 s at a sampling rate of 15 Hz (Langevin et al., 2021; van Lier et al., 2020). Note that due to the different sensing technology employed by PPG and ECG for HR measurement, the signal waveforms exhibit substantial disparities. Consequently, the time-series data of these two signals was not compared.

#### 2.6.3 Parameter comparison: Bland-Altman plots

To compare the measurements of the EmotiBit PPG and the PsychoBit ECG as well as the EDA signals, a Bland-Altman ratio (BAr) was computed (Bland and Altman, 1986; Langevin et al., 2021; Orphanidou et al., 2015; Schäfer and Vagedes, 2013; van Lier et al., 2020). The primary focus was to assess the absolute agreement between the devices, for which Bland-Altman analyses and Bland-Altman ratios offer a direct and well-established framework. The ratio quantifies the relative agreement between two devices and is defined as


$$\begin{array}{l} BAr=\frac{1.96\cdot SD}{A_{pm}}, \end{array}$$

where the 95% CI of the standard deviation is divided by the average of the pairwise means (*A*_*pm*_). A BAr < 0.01 is considered as an excellent agreement, values between 0.01 and 0.1 are considered as a good agreement, values between 0.1 and 0.2 as a moderate agreement, and values >0.2 are defined as insufficient agreement. To visualize differences between methods in detail, Bland-Altman plots were created as well. The mean values of two measurements were plotted on the abscissa (the x-axis) and the difference between the two values on the ordinate (the y-axis). The 95% confidence intervals (CIs) of the differences were indicated to be interpreted as limits of agreement. To compare the signals of devices per condition, the mean values per participant were first log-transformed with base 10 (Euser et al., 2008). For each parameter, additional value boundaries depicting ±10% of the biological plausible values following previous work to add to the interpretability of observed values (see Supplementary Tables 5, 7) (Boucsein, 2012; Braithwaite et al., 2013; Langevin et al., 2021; O'Neal et al., 2016; van Lier et al., 2020). Note that these fixed thresholds do not account for the actual variability in the data and were thus not used for interpretation of signal comparability. Additionally, violin plots of the z-scored parameters of interest were created to visualize the distributions of data points across conditions and devices.

## 3 Results

### 3.1 CVA processing

Several processing pipelines were implemented before assessing the comparability of the devices. The pipelines were tested in various combinations, though not all were originally intended during development. Therefore, only pipelines successfully applied to all runs were included in the signal quality assessment (for an overview, see Supplementary Table 6). The SQI revealed which processing pipeline yielded the highest percentage of good epochs for both the ECG and PPG signals (see Supplementary Table 1). The pipeline applied the data cleaning method implemented by Elgendi et al. (2010) for ECG data and the method employed by Langevin et al. (2021) for PPG data. Peak detection was performed using the ECG method by Nabian et al. (2018) and the peak detection algorithm by Elgendi et al. (2013) for PPG (see Table 1). This yielded 97.76% of epochs rated with sufficient quality for ECG and 89.5% for PPG. The primary reason for ECG epoch rejection was an insufficient correlation between *R*-peaks (< 0.66) affecting 2.2% of epochs. For PPG, the main reason was a ratio of the maximum to the minimum beat-to-beat interval larger than 2.2 (9.1%).

### 3.2 EDA processing

Several processing pipelines consisting of data cleaning, signal decomposition, and peak detection for the EDA signals were implemented to comprehensively test the comparability of devices. The pipeline combinations were not all compatible (see Supplementary Table 3). No clear optimal choice for preprocessing emerged from cleaning, decomposition, and peak detection methods.

While the cross-correlations did not yield differentiable results for the various pipelines, the inspection of the SCR peaks revealed a gross overestimation of peaks per minute for several methods in either the reference device or the EmotiBit or both. According to Boucsein (2012), a realistic number of peaks per minute during high arousal states should be up to 25 peaks/minute, whereas some processing pipelines yielded more than 100 peaks/minute. The pipelines were thus not used for further parameter comparison, assuming that the results were inaccurate for both the reference device and EmotiBit. In the same line of reasoning, one pipeline yielded empty or close-to-zero values for the amplitudes of the SCRs and was also excluded for parameter comparison. Several processing pipelines resulted in the automatic exclusion of one or more runs due to the incompatibility of the methods used per step.

For illustration of the parameter comparison, the processing pipeline that resulted in the smallest standard deviation of differences between means of devices was chosen, i.e., the pipeline resulting in the most consistent differences between devices across runs (for an overview, see Supplementary Table 3). The difference in body location between devices during data collection likely caused differences in measurements larger than 10% of the range of plausible values (the definition of the boundaries). Therefore, we assumed that the least variance within differences reflected the most robust processing for signal comparison rather than the mean of the difference. Note that the chosen analysis pipeline is not necessarily the best-suited for other analyses, for instance, for investigating the effect of conditions on the EDA.

### 3.3 Parameter comparison: CVA

**Figure 5:**
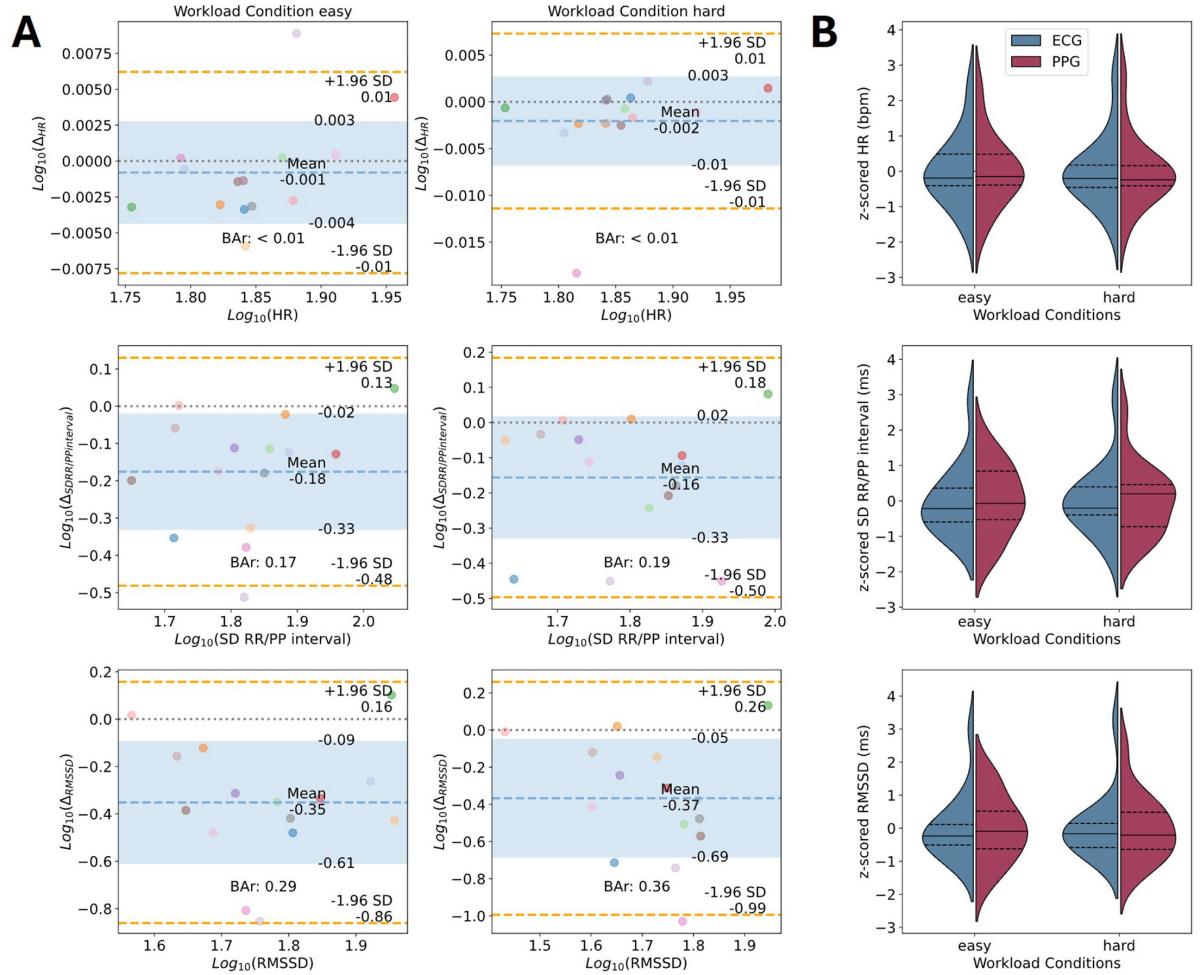
Device comparison of CVA parameters of interest. **(A)** Bland-Altman plots for the parameters' comparison of the CVA signals in terms of the log-transformed mean HR, the SD of the RR/PP intervals, and the RMSSD. Colored dots represent individual participants during the easy condition (left) and the hard condition (right). Each color corresponds to one participant. The x-axis corresponds to the average of the two measures for one parameter, and the y-axis shows the difference between the two measures. The theoretical difference of zero is marked as a gray dotted line. The observed mean difference is plotted as a dashed blue line with the standard deviations marked as blue areas. The orange lines represent the observed 95% confidence interval limits. The computed BAr is indicated within each plot. **(B)** Violin plots of z-scored parameters of interest per condition for ECG (blue) and EmotiBit PPG (red). The median (solid lines) and 1st and 3rd quartiles (dashed lines) for each measured parameter of interest are indicated per device. Bar, Bland-Altman ratio; Δ, Mean difference; ECG, Electrocardiography; HR, Heart rate; PPG, Photoplethysmography; RMSSD: Root mean square of successive differences; SCL, Skin conductance level; SCR, Skin conductance response; SD, Standard deviation.

The Bland-Altman plots for CVA parameters of interest are shown in Figure 5. Detailed results for all processing pipelines can be found in Supplementary Tables 6, 7, including data on original scales (i.e., not log-transformed; see Supplementary Figure 1). The mean HR estimate from the EmotiBit PPG signal is close to the estimate of the PsychoBit ECG signal with an average difference of −0.1 ± 0.62 bpm with a 95% CI of [−1.31, 1.11] bpm for the easy workload condition and −0.31 ± 0.73 bpm with a 95% CI of [−1.73, 1.12] bpm for the hard workload condition. The corresponding BAr of the log-transformed data indicated excellent agreement (easy: BAr < 0.01, hard: BAr = 0.01; see Figure 5). The results of all runs are within the biologically plausible boundaries of ± 5 bpm (see Supplementary Table 7). The difference between the devices regarding the RR/PP interval SD were observed to be −26.36 ± 24.65 ms and a 95% CI of [−74.68, 21.95] ms for the easy workload condition, and −23.41 ± 28.46 ms with a 95% CI of [−79.18, 32.37] ms for the hard workload condition. The BAr of log-transformed data indicated a moderate agreement (easy: BAr = 0.17, hard: BAr = 0.19; see Figure 5) and the majority of runs lay within the agreement boundaries (86.36%). The RMSSD of the peak intervals of the EmotiBit PPG was on average −51.88 ± 41.49 ms lower than that of the PsychoBit ECG with a 95% CI of [−133.19, 29.44] ms for the easy, and −51.78 ± 52.54 ms with a 95% CI of [−154.76, 51.19] ms for the hard workload condition. The BAr of log-transformed data indicated insufficient agreement (easy: BAr = 0.29, hard: BAr = 0.36; see Figure 5).

### 3.4 Signal comparison: EDA

**Figure 6:**
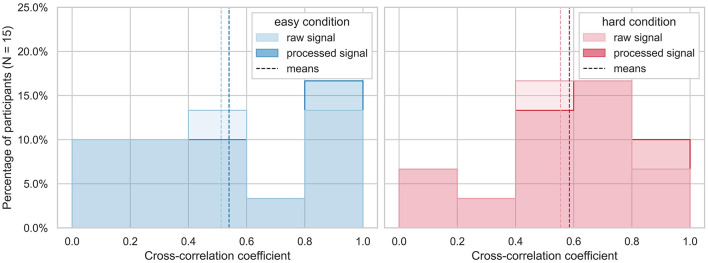
Highest cross-correlation coefficients across subjects. The highest cross-correlation of raw (top) and processed (bottom) EDA signals of is plotted as histogram devices between −8 and +8 sample time lags (corresponding to −0.53 to 0.53 s with a sampling rate of 15 Hz) is plotted for each run divided by easy (blue) and hard (red) condition of the workload task. The mean cross-correlations are indicated as dashed lines.

All tested processing pipelines resulted in cross-correlation coefficients ranging from 0.52 to 0.54 for the easy and 0.56 to 0.59 for the hard runs. In the case of the pipelines with the highest correlation coefficients, 5/15 runs obtained a cross-correlation higher than 0.8 for the easy condition of the task. For the hard condition of the task, 3/15 runs obtained a cross-correlation higher than 0.8 (see Figure 6). This is considered a very high correlation (Evans, 1996). Cross-correlation of the raw data of both devices yielded mean coefficients of 0.51 for easy and 0.55 for hard runs, respectively (see Figure 6).

### 3.5 Parameter comparison: EDA

**Figure 7:**
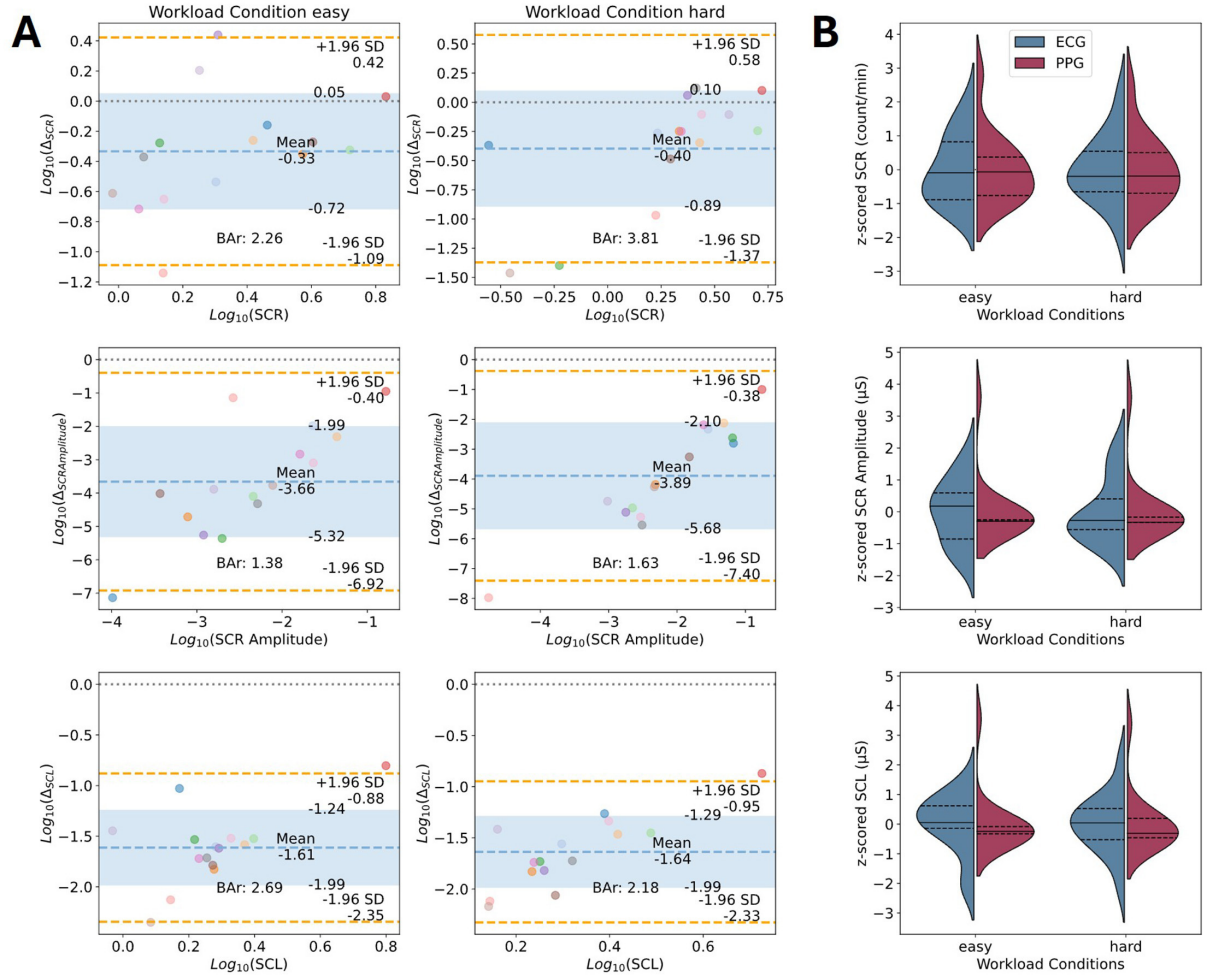
Device comparison of EDA parameters of interest. **(A)** Bland-Altman plots for the parameters' comparison of the EDA signals in terms of the log-transformed mean SCL, the number of SCRs per minute, and the mean amplitude of SCRs. Colored dots represent individual participants during the easy condition (left), and the hard condition (right). Each color corresponds to one participant. The x-axis corresponds to the average of the two measures for one parameter, and the y-axis shows the difference between the two measures. The theoretical difference of zero is marked as a gray dotted line. The observed mean difference is plotted as a dashed blue line with the standard deviations marked as blue areas. The orange lines represent the observed 95% confidence interval limits. The computed BAr is indicated within each plot. **(B)** Violin plots of z-scored parameters of interest per condition for ECG (blue) and EmotiBit PPG (red). The median (solid lines) and 1st and 3rd quartiles (dashed lines) for each measured parameter of interest are indicated per device. Abbeviations: Bar, Bland-Altman ratio; Δ, Mean difference; ECG, Electrocardiography; HR, Heart rate; PPG, Photoplethysmography; RMSSD, Root mean square of successive differences; SCL, Skin conductance level; SCR, Skin conductance response; SD, Standard deviation.

The results of the parameters comparison for the EDA data are illustrated through Bland-Altman plots (see Figure 7; for detailed results see Supplementary Figure 2, Supplementary Tables 4, 5). The pipeline underlying the presented Bland-Altman plots used the BioSSPy default filter parameters for data cleaning, the convex optimization approach introduced by Greco et al. (2016) for signal decomposition, and finally the peak detection by Nabian et al. (2018) as implemented in the Neurokit library (for details, see Table 1). The parameters that were compared between the devices were the mean SCL, the SCRs per minute, and the amplitude of the SCRs. The mean SCL estimate differed by −12.32 ± 3.76 μS for the EmotiBit and PsychoBit with a 95% CI of [−19.68, −4.96] μS in the easy workload condition and −13.57 ± 3.14 μS with a 95% CI of [−19.73, −7.41] μS in the hard workload condition. The BAr of log-transformed values indicated insufficient agreement (easy: BAr = 2.69, hard: BAr = 2.18; see Figure 7). The SCRs per minute derived from the EmotiBit differed on average by −1.6 ± 1.82 from the count of the PsychoBit with a 95% CI of [−5.17, 1.96] for the easy condition and −1.31 ± 1.53 SCRs with a 95% CI of [−4.3, 1.68] for the hard condition. The BAr agreement was insufficient (easy: BAr = 2.26, hard: BAr = 3.81). The amplitudes of the EmotiBit SCRs were on average estimated smaller than those of the PsychoBit (easy: −0.44 ± 0.28 μS, 95% CI of [−0.99, 0.12] μS; hard: −0.76 ± 0.51 μS, 95% CI of [−1.77, 0.24] μS). The BAr of log-transformed data implied insufficient agreement (easy: BAr = 1.38, hard: BAr = 1.63; see Figure 7).

## 4 Discussion

The purpose of this study was to assess the validity of the EmotiBit wearable sensor for monitoring CVA and EDA during task-induced cognitive workload. We positioned the EmotiBit PPG on the upper arm, a realistically applicable site for hands-free monitoring in mobile work settings. Using a standardized validity assessment protocol (Langevin et al., 2021; van Lier et al., 2020), we compared PPG-derived HR and HRV measures as well as EDA indices against an ECG and palmar EDA system (BITalino PsychoBit) during two levels of cognitive workload.

### 4.1 CVA

Our results demonstrate excellent agreement between PPG-based HR estimates from the EmotiBit and ECG-based HR measures, with a Bland-Altman ratio (BAr) indicative of excellent criterion validity (see Figure 5). The 95% CIs indicated that the measures could differ by around one to two beats per minute at most. This applied to runs of both the easy and hard workload conditions. This is an important finding for applied neuroergonomics, as HR is a well-established physiological marker of mental effort and task engagement (Charles and Nixon, 2019; Da et al., 2019; Hughes et al., 2019; Jorna, 1992). Its non-invasive, wearable design allows for data collection in naturalistic settings, thus minimizing laboratory constraints and enhancing ecological validity. HR from the EmotiBit PPG can serve as a reliable surrogate for ECG in mobile workload monitoring systems, enabling real-time physiological assessments in operational environments such as safety-critical industries, remote work monitoring, and adaptive workstations. Furthermore, its integration with other biometric signals, such as temperature and motion, offers the possibility of multi-modal physiological research. In practical applications, such as fitness tracking and stress detection, the reliability of the EmotiBit ensures its efficacy as an alternative to ECG, which is often unwieldy and requires specialist equipment. We demonstrated the feasibility of using the Emotibit PPG device for measuring CVA. Nevertheless, the recorded sample of participants was relatively small, and thus robustness and generalizability of observed results is likely affected. We recommend that another research team should replicate our findings with a large and diverse sample to enhance the generalizability of findings.

Conversely, HRV metrics (SD and RMSSD) derived from PPG peak intervals showed moderate to insufficient BAr agreement with ECG (see Figure 5). The SDs of peak-to-peak intervals were in moderate agreement between devices, whereas the RMSSDs were of insufficient agreement. This result aligns with prior validation work of the EmotiBit (Langevin et al., 2021) as well as other PPG devices (O'Grady et al., 2024; Schäfer and Vagedes, 2013; Taoum et al., 2022). To be able to test the biological plausibility of the PPG signal in line with the established criterion validity method (Langevin et al., 2021; van Lier et al., 2020), all signal wavelengths (green, red, infrared) were averaged to create the standard PPG signal stream. The resulting signal might not be reliable to assess the HRV-related measures of SD and RMSSD of the PP intervals, similar to other PPG wearable devices (for a review, see Schäfer and Vagedes, 2013). However, the EmotiBit stores the recorded wavelength signals separately, thus allowing for separate analysis of the three signals if desired.

**Figure 8:**
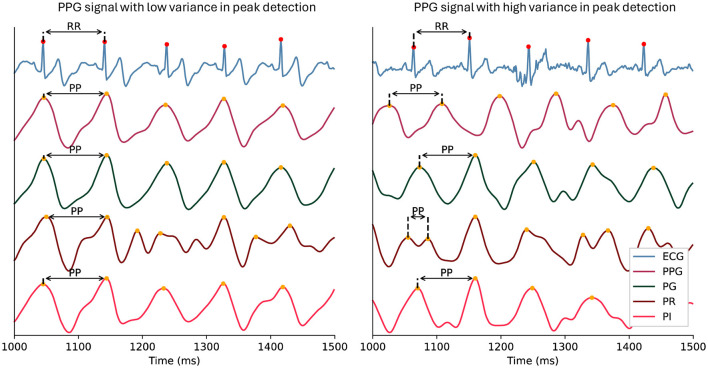
Example of peak detection in PPG signal streams. The figure illustrates the effect of averaging the three light wavelength signals of a PPG (PG, PR, PI) on the peak detection comparability to an ECG in the case of low variance between the signal streams (left) compared to high variance in the signal streams (right). In the left panel, an example of consistent peak-to-peak intervals across signals is highlighted with arrows. The right panel shows inconsistent peak-to-peak intervals across signals. ECG, Electrocardiography; PG: green PPG signal; PI, infrared PPG signal; PP, P-peak interval PPG, photoplethysmography; PR, red PPG signal; RR, R-peak interval.

The green light spectrum is best suited for HRV-related measurements compared to red wavelengths (see Figure 1; Maeda et al., 2011). Red and infrared light spectra offer a more detailed insight into oxygenation levels of hemoglobin as they penetrate deeper into the tissue (Nitzan et al., 2014). For the same reason, this can introduce more noise and variability into the PPG signal compared to the ECG signal, especially in wearable devices (see Figure 8; Biswas et al., 2019). Therefore, it is recommended to use the EmotiBit PPG signal streams independently from each other, depending on the effect of interest.

### 4.2 EDA

Our findings indicate insufficient agreement between EmotiBit EDA (upper arm) and BITalino EDA (palm) for all EDA parameters (SCL, SCR count, SCR amplitude) as indicated by the BAr ratios being considerably larger than 0.2 regardless of the processing choices, thus indicating insufficient agreement (see Figure 7 and Supplementary Table 4). Our findings extend prior reports of EmotiBit's limited EDA performance (Haratian, 2024; Langevin et al., 2021) and are consistent with broader evidence on anatomical variations in sweat gland density (Baker, 2019). Given that the placement of the devices differed substantially, we expected mean signal differences to some extent. The palm has more sweat glands compared to the upper arm (Baker, 2019). Therefore, it is highly unlikely to observe no difference in signal level between the devices. The finding that the upper arm site proved suboptimal for capturing EDA reactivity relative to the palm reflects a broader challenge in the field of neuroergonomics: the data acquisition in naturalistic settings often prompts a trade-off decision between the comfortable, unobtrusive functionality of a device and optimal signal recording sites. It is recommended that future validation work systematically harmonize placement locations when possible. Although the quality of wearable sensors has improved in recent years (Huhn et al., 2022), emerging alternatives to body EDA sensors, such as facial thermal imaging, could offer a more reliable and accurate solution for workload detection in the wild (Gioia et al., 2022).

## 5 Conclusion

We evaluated the validity of utilizing the EmotiBit wearable sensor for cardiovascular and electrodermal measures during cognitive workload. We employed a standardized protocol for signal comparisons following an analysis of both signals using various processing pipelines. Our results pertaining to the CVA measures highlight that the EmotiBit can be employed with high biological plausibility in a natural setup and delivers reliable HR recordings, even under various workload conditions. However, when it comes to HRV and EDA measures, comparable measurement values were not observed, although the inherent differences between the measured body locations could be held accountable for this observed disagreement. While the EmotiBit HR monitoring is field-ready for workload detection and adaptive human-system interaction, observed HRV and EDA limitations necessitate algorithmic improvements or site-optimized solutions for high-resolution cognitive state monitoring. The trade-off between placement convenience and measurement fidelity remains an unresolved challenge when moving from laboratory ECG setups to consumer-oriented wearable solutions. Thus, research in the field of consumer neuroergonomics should focus on integrating high-quality multimodal physiological sensing with user comfort to advance real-time workload-adaptive environments.

## Data Availability

The data analyzed in this study is subject to the following licenses/restrictions: data and code pertaining to the study will be made available upon request. Requests to access these datasets should be directed to anna.vorreuther@iat.uni-stuttgart.de.
