# Supplementary material for: Validation of the EmotiBit wearable sensor for heart-based measures under varying workload conditions
Source: Front Neuroergon. 2025 Jun 18;6:1585469. doi: 10.3389/fnrgo.2025.1585469 (PMC12213893; doi:10.3389/fnrgo.2025.1585469)
Supplement: Supplementary file 1 [file Data_Sheet_1.docx]

Supplemental Material

Table 1
*Signal Quality Indexing for the CVA Processing Pipelines Including ECG Downsampling*

| Processing Pipeline | Total Sessions | ECG | | PPG | |
| --- | --- | --- | --- | --- | --- |
|  |  | Good Epochs (%) | Bad Epochs (%) | Good Epochs (%) | Bad Epochs (%) |
| ECG Elgendi & Nabian; PPG Langevin & Elgendi * | 30 | 97.76 | 2.24 | 89.5 | 10.5 |
| ECG Elgendi & Nabian; PPG Elgendi & Elgendi | 30 | 97.76 | 2.24 | 77.27 | 22.73 |
| ECG Neurokit & Neurokit; PPG Langevin & Elgendi | 30 | 96.27 | 3.73 | 89.5 | 10.5 |
| ECG Neurokit & Neurokit; PPG Elgendi & Elgendi | 30 | 96.27 | 3.73 | 77.27 | 22.73 |
| ECG Elgendi & Neurokit; PPG Langevin & Elgendi | 30 | 95.32 | 4.68 | 89.5 | 10.5 |
| ECG Elgendi & Neurokit; PPG Elgendi & Elgendi | 30 | 95.32 | 4.68 | 77.27 | 22.73 |
| ECG BioSSPy & Neurokit; PPG Langevin & Elgendi | 30 | 95.29 | 4.71 | 89.5 | 10.5 |
| ECG BioSSPy & Neurokit; PPG Elgendi & Elgendi | 30 | 95.29 | 4.71 | 77.27 | 22.73 |
| ECG Neurokit & Nabian; PPG Langevin & Elgendi | 30 | 93.89 | 6.11 | 89.5 | 10.5 |
| ECG Neurokit & Nabian; PPG Elgendi & Elgendi | 30 | 93.89 | 6.11 | 77.27 | 22.73 |
| ECG BioSSPy & Nabian; PPG Langevin & Elgendi | 30 | 92.37 | 7.63 | 89.5 | 10.5 |
| ECG BioSSPy & Nabian; PPG Elgendi & Elgendi | 30 | 92.37 | 7.63 | 77.27 | 22.73 |
| ECG Langevin & Neurokit; PPG Langevin & Elgendi | 30 | 89.49 | 10.51 | 89.5 | 10.5 |
| ECG Langevin & Neurokit; PPG Elgendi & Elgendi | 30 | 89.49 | 10.51 | 77.27 | 22.73 |
| ECG Elgendi & Elgendi; PPG Langevin & Elgendi | 30 | 89.38 | 10.62 | 89.5 | 10.5 |
| ECG Elgendi & Elgendi; PPG Elgendi & Elgendi | 30 | 89.38 | 10.62 | 77.27 | 22.73 |
| ECG Langevin & Nabian; PPG Langevin & Elgendi | 30 | 89.29 | 10.71 | 89.5 | 10.5 |
| ECG Langevin & Nabian; PPG Elgendi & Elgendi | 30 | 89.29 | 10.71 | 77.27 | 22.73 |
| ECG BioSSPy & Elgendi; PPG Langevin & Elgendi | 30 | 82.75 | 17.25 | 89.5 | 10.5 |
| ECG BioSSPy & Elgendi; PPG Elgendi & Elgendi | 30 | 82.75 | 17.25 | 77.27 | 22.73 |
| ECG Neurokit & Elgendi; PPG Langevin & Elgendi | 30 | 77.44 | 22.56 | 89.5 | 10.5 |
| ECG Neurokit & Elgendi; PPG Elgendi & Elgendi | 30 | 77.44 | 22.56 | 77.27 | 22.73 |
| ECG Langevin & Elgendi; PPG Langevin & Elgendi | 28 | 64.74 | 35.26 | 89.27 | 10.73 |
| ECG Langevin & Elgendi; PPG Elgendi & Elgendi | 28 | 64.74 | 35.26 | 76.46 | 23.54 |
| ECG BioSSPy & Gamboa; PPG Langevin & Elgendi | 3 | 1.01 | 98.99 | 96.2 | 3.8 |
| ECG BioSSPy & Gamboa; PPG Elgendi & Elgendi | 3 | 1.01 | 98.99 | 91.39 | 8.61 |
| ECG Neurokit & Neurokit; PPG Nabian & Elgendi | - | - | - | - | - |
| ECG Neurokit & Nabian; PPG Nabian & Elgendi | - | - | - | - | - |
| ECG Neurokit & Gamboa; PPG Nabian & Elgendi | - | - | - | - | - |
| ECG Neurokit & Elgendi; PPG Nabian & Elgendi | - | - | - | - | - |
| ECG Langevin & Neurokit; PPG Nabian & Elgendi | - | - | - | - | - |
| ECG Langevin & Nabian; PPG Nabian & Elgendi | - | - | - | - | - |
| ECG Langevin & Gamboa; PPG Nabian & Elgendi | - | - | - | - | - |
| ECG Langevin & Elgendi; PPG Nabian & Elgendi | - | - | - | - | - |
| ECG BioSSPy & Neurokit; PPG Nabian & Elgendi | - | - | - | - | - |
| ECG BioSSPy & Nabian; PPG Nabian & Elgendi | - | - | - | - | - |
| ECG BioSSPy & Gamboa; PPG Nabian & Elgendi | - | - | - | - | - |
| ECG BioSSPy & Elgendi; PPG Nabian & Elgendi | - | - | - | - | - |
| ECG Elgendi & Neurokit; PPG Nabian & Elgendi | - | - | - | - | - |
| ECG Elgendi & Nabian; PPG Nabian & Elgendi | - | - | - | - | - |
| ECG Elgendi & Gamboa; PPG Nabian & Elgendi | - | - | - | - | - |
| ECG Elgendi & Elgendi; PPG Nabian & Elgendi | - | - | - | - | - |

*Note*. The results of the signal quality indexing of ECG and PPG data for used processing pipelines. The number of total sessions processed by each pipeline equals 30 if all recorded runs were successfully processed. Lower numbers indicate incompatible processing steps. The percentage of 10-s epochs qualified as “good” or “bad” by the signal quality indexing is shown. The pipeline annotation consists of “ECG data cleaning method & peak detection method; PPG data cleaning method & peak detection method”. The pipeline configuration used for creating the Bland-Altman plots is marked with an asterisk.

Table 2
*Signal Quality Indexing for the CVA Processing Pipelines with Original ECG Sampling Frequency*

| Processing Pipeline | Total Sessions | ECG | | PPG | |
| --- | --- | --- | --- | --- | --- |
|  |  | Good Epochs (%) | Bad Epochs (%) | Good Epochs (%) | Bad Epochs (%) |
| ECG Elgendi & Nabian; PPG Langevin & Elgendi | 30 | 98.08 | 1.92 | 89.5 | 10.5 |
| ECG Elgendi & Nabian; PPG Elgendi & Elgendi | 30 | 98.08 | 1.92 | 77.27 | 22.73 |
| ECG Neurokit & Neurokit; PPG Langevin & Elgendi | 30 | 97.19 | 2.81 | 89.5 | 10.5 |
| ECG Neurokit & Neurokit; PPG Elgendi & Elgendi | 30 | 97.19 | 2.81 | 77.27 | 22.73 |
| ECG Elgendi & Neurokit; PPG Langevin & Elgendi | 30 | 96.5 | 3.5 | 89.5 | 10.5 |
| ECG Elgendi & Neurokit; PPG Elgendi & Elgendi | 30 | 96.5 | 3.5 | 77.27 | 22.73 |
| ECG Neurokit & Nabian; PPG Langevin & Elgendi | 30 | 94.55 | 5.45 | 89.5 | 10.5 |
| ECG Neurokit & Nabian; PPG Elgendi & Elgendi | 30 | 94.55 | 5.45 | 77.27 | 22.73 |
| ECG BioSPPy & Neurokit; PPG Langevin & Elgendi | 30 | 93.97 | 6.03 | 89.5 | 10.5 |
| ECG BioSPPy & Neurokit; PPG Elgendi & Elgendi | 30 | 93.97 | 6.03 | 77.27 | 22.73 |
| ECG BioSPPy & Nabian; PPG Langevin & Elgendi | 30 | 93.94 | 6.06 | 89.5 | 10.5 |
| ECG BioSPPy & Nabian; PPG Elgendi & Elgendi | 30 | 93.94 | 6.06 | 77.27 | 22.73 |
| ECG Elgendi & Elgendi; PPG Langevin & Elgendi | 29 | 91.99 | 8.01 | 89.28 | 10.72 |
| ECG Elgendi & Elgendi; PPG Elgendi & Elgendi | 29 | 91.99 | 8.01 | 76.66 | 23.34 |
| ECG Langevin & Nabian; PPG Langevin & Elgendi | 30 | 90.84 | 9.16 | 89.5 | 10.5 |
| ECG Langevin & Nabian; PPG Elgendi & Elgendi | 30 | 90.84 | 9.16 | 77.27 | 22.73 |
| ECG BioSPPy & Elgendi; PPG Langevin & Elgendi | 30 | 80.86 | 19.14 | 89.5 | 10.5 |
| ECG BioSPPy & Elgendi; PPG Elgendi & Elgendi | 30 | 80.86 | 19.14 | 77.27 | 22.73 |
| ECG Langevin & Neurokit; PPG Langevin & Elgendi | 30 | 75.57 | 24.43 | 89.5 | 10.5 |
| ECG Langevin & Neurokit; PPG Elgendi & Elgendi | 30 | 75.57 | 24.43 | 77.27 | 22.73 |
| ECG Neurokit & Elgendi; PPG Langevin & Elgendi | 30 | 73.31 | 26.69 | 89.5 | 10.5 |
| ECG Neurokit & Elgendi; PPG Elgendi & Elgendi | 30 | 73.31 | 26.69 | 77.27 | 22.73 |
| ECG Langevin & Elgendi; PPG Langevin & Elgendi | 29 | 65.37 | 34.63 | 89.5 | 10.5 |
| ECG Langevin & Elgendi; PPG Elgendi & Elgendi | 29 | 65.37 | 34.63 | 77.1 | 22.9 |
| ECG BioSPPy & Gamboa; PPG Langevin & Elgendi | 24 | 29.52 | 70.48 | 91.07 | 8.93 |
| ECG BioSPPy & Gamboa; PPG Elgendi & Elgendi | 24 | 29.52 | 70.48 | 77.73 | 22.27 |
| ECG Neurokit & Neurokit; PPG Nabian & Elgendi | - | - | - | - | - |
| ECG Neurokit & Nabian; PPG Nabian & Elgendi | - | - | - | - | - |
| ECG Neurokit & Gamboa; PPG Nabian & Elgendi | - | - | - | - | - |
| ECG Neurokit & Elgendi; PPG Nabian & Elgendi | - | - | - | - | - |
| ECG Langevin & Neurokit; PPG Nabian & Elgendi | - | - | - | - | - |
| ECG Langevin & Nabian; PPG Nabian & Elgendi | - | - | - | - | - |
| ECG Langevin & Gamboa; PPG Nabian & Elgendi | - | - | - | - | - |
| ECG Langevin & Elgendi; PPG Nabian & Elgendi | - | - | - | - | - |
| ECG BioSPPy & Neurokit; PPG Nabian & Elgendi | - | - | - | - | - |
| ECG BioSPPy & Nabian; PPG Nabian & Elgendi | - | - | - | - | - |
| ECG BioSPPy & Gamboa; PPG Nabian & Elgendi | - | - | - | - | - |
| ECG BioSPPy & Elgendi; PPG Nabian & Elgendi | - | - | - | - | - |
| ECG Elgendi & Neurokit; PPG Nabian & Elgendi | - | - | - | - | - |
| ECG Elgendi & Nabian; PPG Nabian & Elgendi | - | - | - | - | - |
| ECG Elgendi & Gamboa; PPG Nabian & Elgendi | - | - | - | - | - |
| ECG Elgendi & Elgendi; PPG Nabian & Elgendi | - | - | - | - | - |

*Note*. The results of the signal quality indexing of ECG and PPG data for used processing pipelines excluding the initial downsampling step for the ECG data. The number of total sessions processed by each pipeline equals 30 if all recorded runs were successfully processed. Lower numbers indicate incompatible processing steps. The percentage of 10-s epochs qualified as “good” or “bad” by the signal quality indexing is shown. The pipeline annotation consists of “ECG data cleaning method & peak detection method; PPG data cleaning method & peak detection method”. The pipeline configuration used for creating the Bland-Altman plots is marked with an asterisk.

Table 3
*Cross-Correlation Results for Preprocessing Pipelines*

| Processing pipeline | Easy condition | | Hard condition | | Remark |
| --- | --- | --- | --- | --- | --- |
|  | N_runs_ | Mean | N_runs_ | Mean |  |
| BioSSPy & Greco & Nabian* | 15 | 0.54 | 15 | 0.59 | Smallest SCR SD mean difference between devices (Δμ_SD SCR_ = 1.65) |
| BioSSPy & Greco & Gamboa | 15 | 0.54 | 15 | 0.59 |  |
| BioSSPy & Neurokit & Kim | 15 | 0.54 | 15 | 0.59 |  |
| BioSSPy & Neurokit & Nabian | 15 | 0.54 | 15 | 0.59 |  |
| BioSSPy & Neurokit & Neurokit | 15 | 0.54 | 15 | 0.59 |  |
| BioSSPy & Biopac & Neurokit | 15 | 0.54 | 15 | 0.59 |  |
| Neurokit & Greco & Gamboa | 15 | 0.52 | 15 | 0.56 |  |
| Neurokit & Greco & Nabian | 15 | 0.52 | 15 | 0.56 |  |
| Neurokit & Greco & Neurokit | 15 | 0.52 | 15 | 0.56 |  |
| Raw data | 15 | 0.51 | 15 | 0.55 |  |
| Neurokit & Biopac & Neurokit | 15 | 0.52 | 15 | 0.56 | Mean SCR peaks/minute overestimated for Emotibit (μ_SCR_ = 47.93) |
| Neurokit & Neurokit & Neurokit | 15 | 0.52 | 15 | 0.56 | Mean SCR peaks/minute overestimated for Emotibit (μ_SCR_ = 37.0) |
| BioSSPy & Neurokit & Gamboa | 15 | 0.54 | 15 | 0.59 | Mean SCR peaks/minute overestimated for Psychobit (μ_SCR_ = 33.2) and Emotibit (μ_SCR_ = 42.1) |
| Neurokit & Neurokit & Gamboa | 15 | 0.52 | 15 | 0.56 | Mean SCR peaks/minute overestimated for Psychobit (μ_SCR_ = 99.15) and Emotibit (μ_SCR_ = 113.05) |
| Neurokit & Greco & Gamboa | 15 | 0.52 | 15 | 0.56 | Mean SCR peaks/minute overestimated for Psychobit (μ_SCR_ = 33.48) |
| BioSSPy & Greco & Neurokit | 15 | 0.54 | 15 | 0.59 | NaN values for amplitude calculations |
| BioSSPy & Biopac & Kim | 11 | 0.58 | 6 | 0.55 | Processing failed for multiple runs |
| Neurokit & Neurokit & Kim | 9 | 0.47 | 9 | 0.66 | Processing failed for multiple runs |
| Neurokit & Neurokit & Nabian | 8 | 0.48 | 10 | 0.57 | Processing failed for multiple runs |
| Neurokit & Biopac & Kim | 4 | 0.44 | 3 | 0.55 | Processing failed for multiple runs |
| BioSSPy & Greco & Kim | 15 | 0.54 | 14 | 0.58 | Processing failed for one run |
| Neurokit & Greco & Kim | 14 | 0.51 | 15 | 0.56 | Processing failed for one run |
| BioSSPy & Greco & Gamboa | - | - | - | - | Processing steps not compatible |
| BioSSPy & Greco & Kim | - | - | - | - | Processing steps not compatible |
| BioSSPy & Greco & Nabian | - | - | - | - | Processing steps not compatible |
| BioSSPy & Greco & Neurokit | - | - | - | - | Processing steps not compatible |
| BioSSPy & Biopac & Gamboa | - | - | - | - | Processing steps not compatible |
| BioSSPy & Biopac & Nabian | - | - | - | - | Processing steps not compatible |
| Neurokit & Greco & Kim | - | - | - | - | Processing steps not compatible |
| Neurokit & Greco & Nabian | - | - | - | - | Processing steps not compatible |
| Neurokit & Greco & Neurokit | - | - | - | - | Processing steps not compatible |
| Neurokit & Biopac & Gamboa | - | - | - | - | Processing steps not compatible |
| Neurokit & Biopac & Nabian | - | - | - | - | Processing steps not compatible |

*Note*. The mean of the cross-correlation coefficients (if applicable) for all preprocessing pipeline configurations per task condition. The pipeline annotation consists of “data cleaning method & signal decomposition method & peak detection method”. The pipeline configuration used for creating the Bland-Altman plots is marked with an asterisk.

Figure 1
*Bland-Altman and Violin Plots for CVA Parameters of Interest (Original Scale)*
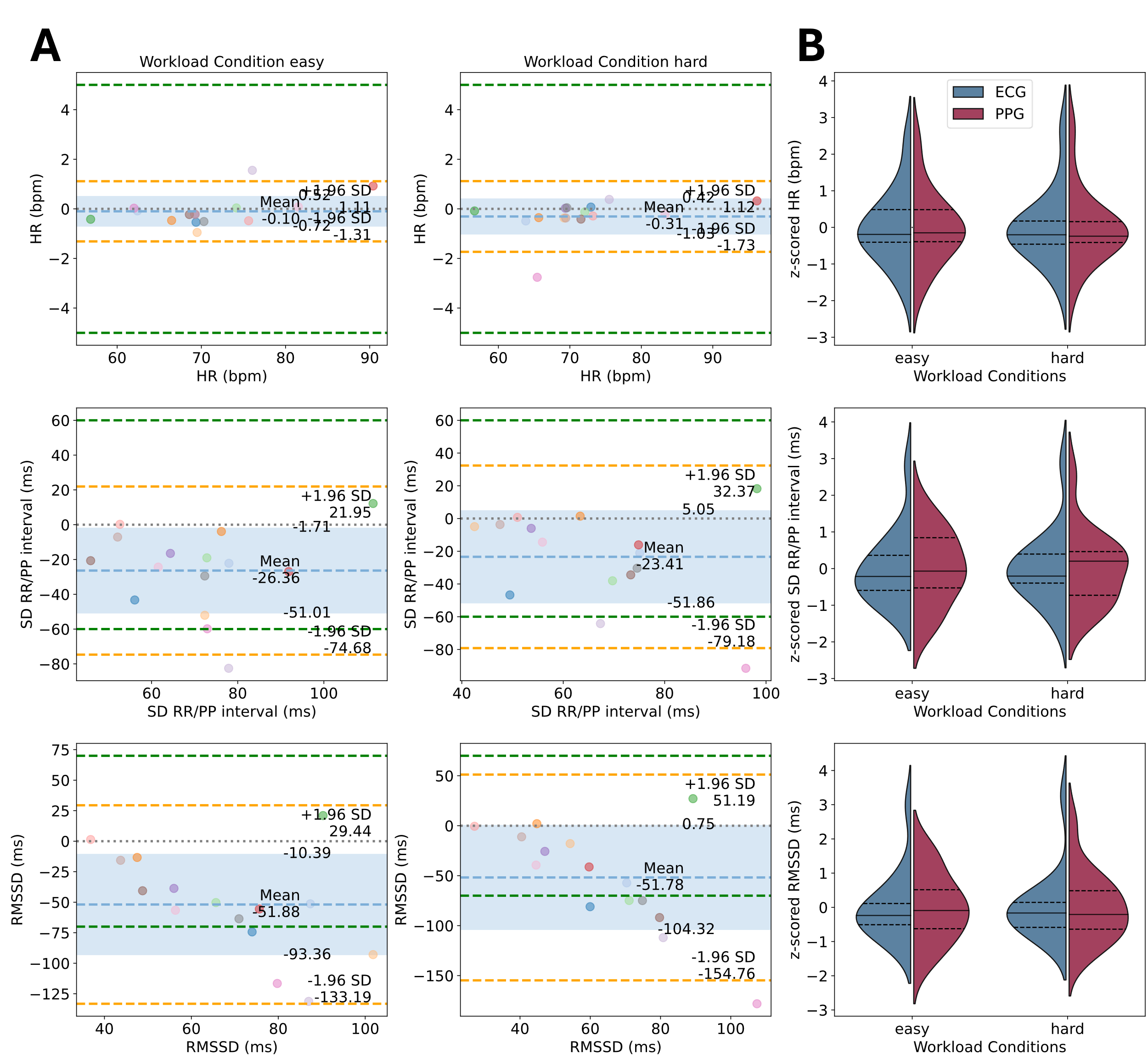


*Note*. A) Bland-Altman plots for the parameters’ comparison of the CVA signals in terms of the mean HR, the SD of the RR/PP intervals, and the RMSSD. Colored dots represent individual participants during the easy condition (left) and the hard condition (right). Each color corresponds to one participant. The x-axis corresponds to the average of the two measures for one parameter, and the y-axis shows the difference between the two measures. A theoretical difference of zero is marked as a grey dotted line. The observed mean difference is plotted as a dashed blue line with the standard deviations marked as blue areas. The orange lines represent the observed 95% confidence interval limits. Green lines show biologically plausible difference values relative to zero. B) Violin plots of z-scored parameters of interest per condition for ECG (blue) and EmotiBit PPG (red). The median (solid lines) and 1^st^ and 3^rd^ quartiles (dashed lines) for each measured parameter of interest are indicated per device. Abbreviations: BAr: Bland-Altman ratio; ECG: Electrocardiography; HR: Heart rate; PPG: Photoplethysmography; RMSSD: Root mean square of successive differences; SCL: Skin conductance level; SCR: Skin conductance response; SD: Standard deviation

Figure 2
*Bland-Altman and Violin Plots for EDA Parameters of Interest (Original Scale)*


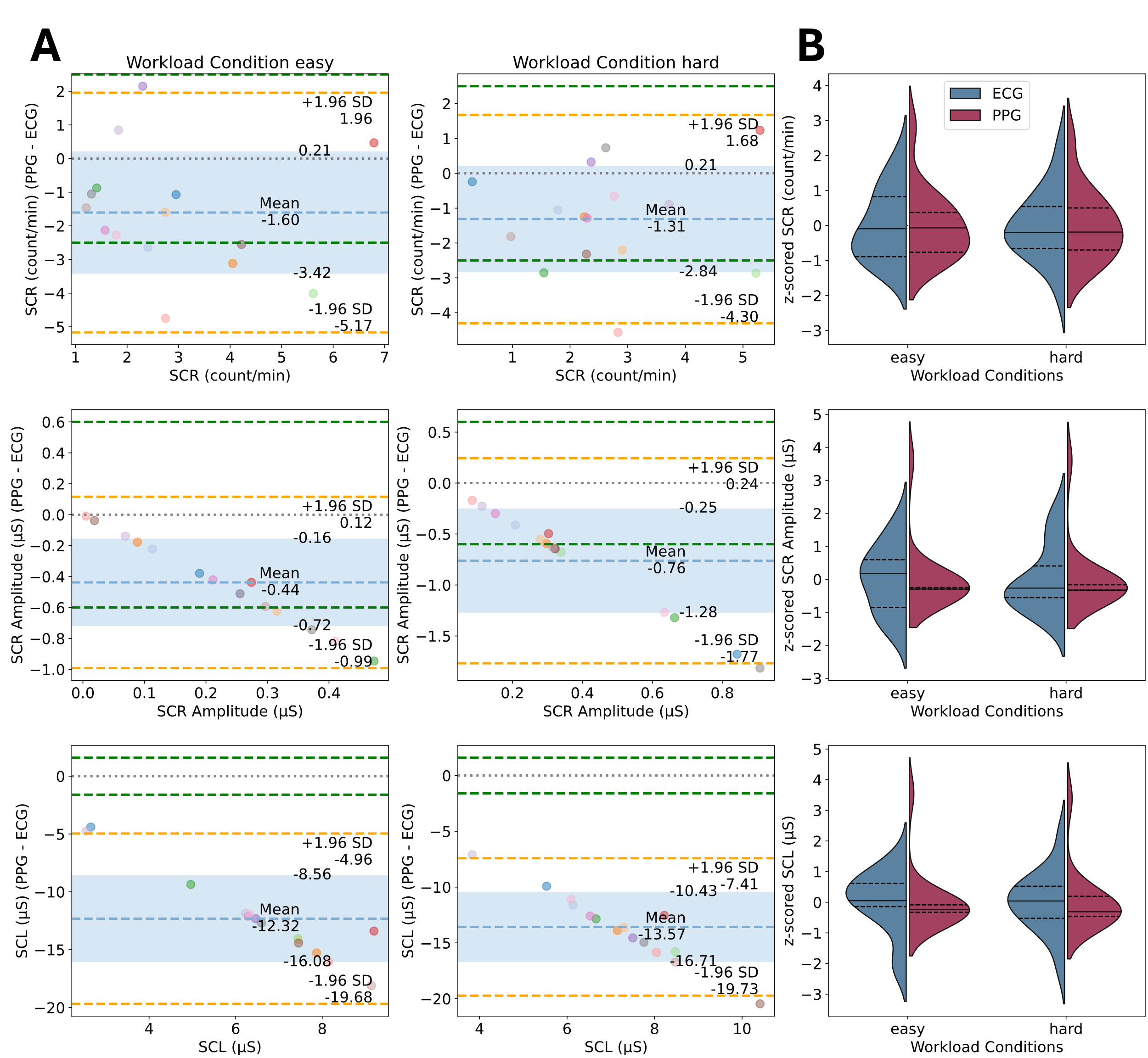


*Note*. A) Bland-Altman plots for the parameters’ comparison of the EDA signals in terms of the mean SCL, the number of SCRs per minute, and the mean amplitude of SCRs. Colored dots represent individual participants during the easy condition (left) and the hard condition (right). Each color corresponds to one participant. The x-axis corresponds to the average of the two measures for one parameter, and the y-axis shows the difference between the two measures. A theoretical difference of zero is marked as a grey dotted line. The observed mean difference is plotted as a dashed blue line with the standard deviations marked as blue areas. The orange lines represent the observed 95% confidence interval limits. Green lines show biologically plausible difference values relative to zero. B) Violin plots of z-scored parameters of interest per condition for ECG (blue) and EmotiBit PPG (red). The median (solid lines) and 1^st^ and 3^rd^ quartiles (dashed lines) for each measured parameter of interest are indicated per device. Abbreviations: BAr: Bland-Altman ratio; ECG: Electrocardiography; HR: Heart rate; PPG: Photoplethysmography; RMSSD: Root mean square of successive differences; SCL: Skin conductance level; SCR: Skin conductance response; SD: Standard deviation

Table 4
*BAr Results for Log-Transformed EDA Parameters Across Processing Pipelines*

|  |  | Log(SCR) | | | | | Log(SCRAmplitude) | | | | | Log(SCL) | | | | |
| --- | --- | --- | --- | --- | --- | --- | --- | --- | --- | --- | --- | --- | --- | --- | --- | --- |
| Processing pipeline | Workload condition | Mean ± SD | 95% CI | BAr | normality | homoscedasticity | Mean ± SD | 95% CI | BAr | normality | homoscedasticity | Mean ± SD | 95% CI | BAr | normality | homoscedasticity |
| BioSPPy & Greco & Gamboa | easy | -0.48 ± 0.24 | [-0.95, -0.01] | 0.48 | True (W = 0.903, p = 0.105) | False (rho = 0.600, p = 0.018) | -3.58 ± 1.43 | [-6.40, -0.77] | 0.97 | True (W = 0.960, p = 0.698) | False (rho = -0.779, p = 0.001) | -1.61 ± 0.37 | [-2.35, -0.88] | 2.69 | True (W = 0.931, p = 0.283) | True (rho = -0.339, p = 0.216) |
| BioSPPy & Greco & Gamboa | hard | -0.49 ± 0.29 | [-1.05, 0.07] | 0.58 | True (W = 0.895, p = 0.079) | True (rho = 0.511, p = 0.052) | -3.85 ± 1.69 | [-7.16, -0.54] | 1.19 | True (W = 0.951, p = 0.538) | False (rho = -0.914, p = 0.000) | -1.64 ± 0.35 | [-2.33, -0.95] | 2.18 | True (W = 0.965, p = 0.776) | False (rho = -0.743, p = 0.002) |
| BioSPPY & Greco & Kim | easy | -0.44 ± 0.85 | [-2.12, 1.23] | 4.02 | True (W = 0.899, p = 0.093) | False (rho = -0.914, p = 0.000) | -3.79 ± 2.18 | [-8.06, 0.48] | 1.67 | True (W = 0.932, p = 0.290) | False (rho = -0.879, p = 0.000) | -1.61 ± 0.37 | [-2.35, -0.88] | 2.69 | True (W = 0.931, p = 0.283) | True (rho = -0.339, p = 0.216) |
| BioSPPY & Greco & Kim | hard | -0.59 ± 0.82 | [-2.19, 1.01] | 4.94 | False (W = 0.862, p = 0.032) | False (rho = -0.635, p = 0.015) | -4.06 ± 2.06 | [-8.09, -0.03] | 1.72 | True (W = 0.960, p = 0.722) | False (rho = -0.837, p = 0.000) | -1.66 ± 0.35 | [-2.35, -0.98] | 2.19 | True (W = 0.951, p = 0.578) | False (rho = -0.719, p = 0.004) |
| BioSPPY & Greco & Nabian * | easy | -0.33 ± 0.39 | [-1.09, 0.42] | 2.26 | True (W = 0.972, p = 0.889) | False (rho = -0.646, p = 0.009) | -3.66 ± 1.66 | [-6.92, -0.40] | 1.38 | True (W = 0.975, p = 0.925) | False (rho = -0.757, p = 0.001) | -1.61 ± 0.37 | [-2.35, -0.88] | 2.69 | True (W = 0.931, p = 0.283) | True (rho = -0.339, p = 0.216) |
| BioSPPY & Greco & Nabian * | hard | -0.40 ± 0.50 | [-1.37, 0.58] | 3.81 | False (W = 0.821, p = 0.007) | False (rho = -0.800, p = 0.000) | -3.89 ± 1.79 | [-7.40, -0.38] | 1.63 | True (W = 0.954, p = 0.587) | False (rho = -0.871, p = 0.000) | -1.64 ± 0.35 | [-2.33, -0.95] | 2.18 | True (W = 0.965, p = 0.776) | False (rho = -0.743, p = 0.002) |
| BioSPPY & Greco & Neurokit | easy | -0.56 ± 0.68 | [-1.90, 0.78] | 91.57 | True (W = 0.959, p = 0.680) | False (rho = -0.868, p = 0.000) | nan ± nan | [nan, nan] |  | True (W = 0.952, p = 0.631) | True (rho = nan, p = nan) | -1.61 ± 0.37 | [-2.35, -0.88] | 2.69 | True (W = 0.931, p = 0.283) | True (rho = -0.339, p = 0.216) |
| BioSPPY & Greco & Neurokit | hard | -0.44 ± 0.72 | [-1.84, 0.96] | 76.01 | True (W = 0.892, p = 0.072) | False (rho = -0.671, p = 0.006) | nan ± nan | [nan, nan] |  | True (W = 0.956, p = 0.658) | True (rho = nan, p = nan) | -1.64 ± 0.35 | [-2.33, -0.95] | 2.18 | True (W = 0.965, p = 0.776) | False (rho = -0.743, p = 0.002) |
| BioSPPY & Neurokit & Gamboa | easy | nan ± nan | [nan, nan] |  |  |  | -2.48 ± 0.68 | [-3.82, -1.14] | 0.53 | True (W = 0.969, p = 0.840) | True (rho = -0.075, p = 0.791) | -1.62 ± 0.37 | [-2.35, -0.89] | 2.62 | True (W = 0.926, p = 0.240) | True (rho = -0.321, p = 0.243) |
| BioSPPY & Neurokit & Gamboa | hard | nan ± nan | [nan, nan] |  |  |  | -2.67 ± 0.67 | [-3.99, -1.35] | 0.55 | True (W = 0.934, p = 0.310) | True (rho = -0.364, p = 0.182) | -1.65 ± 0.35 | [-2.34, -0.96] | 2.12 | True (W = 0.961, p = 0.718) | False (rho = -0.739, p = 0.002) |
| BioSPPY & Neurokit & Kim | easy | 0.13 ± 0.95 | [-1.72, 1.99] | 2.85 | True (W = 0.918, p = 0.181) | True (rho = -0.250, p = 0.369) | -2.69 ± 1.14 | [-4.93, -0.45] | 1.14 | True (W = 0.928, p = 0.259) | False (rho = -0.529, p = 0.043) | -1.62 ± 0.37 | [-2.35, -0.89] | 2.62 | True (W = 0.926, p = 0.240) | True (rho = -0.321, p = 0.243) |
| BioSPPY & Neurokit & Kim | hard | 0.15 ± 0.56 | [-0.94, 1.25] | 1.8 | True (W = 0.952, p = 0.551) | True (rho = 0.207, p = 0.459) | -2.80 ± 0.89 | [-4.54, -1.06] | 0.99 | True (W = 0.944, p = 0.440) | False (rho = -0.743, p = 0.002) | -1.65 ± 0.35 | [-2.34, -0.96] | 2.12 | True (W = 0.961, p = 0.718) | False (rho = -0.739, p = 0.002) |
| BioSPPY & Neurokit & Nabian | easy | 0.36 ± 0.47 | [-0.56, 1.28] | 0.89 | True (W = 0.934, p = 0.310) | True (rho = -0.257, p = 0.355) | -2.70 ± 0.87 | [-4.40, -1.00] | 0.8 | True (W = 0.929, p = 0.261) | True (rho = -0.182, p = 0.516) | -1.62 ± 0.37 | [-2.35, -0.89] | 2.62 | True (W = 0.926, p = 0.240) | True (rho = -0.321, p = 0.243) |
| BioSPPY & Neurokit & Nabian | hard | 0.24 ± 0.40 | [-0.55, 1.04] | 0.81 | True (W = 0.982, p = 0.983) | True (rho = -0.046, p = 0.869) | -2.82 ± 0.80 | [-4.39, -1.25] | 0.8 | True (W = 0.947, p = 0.477) | True (rho = -0.457, p = 0.087) | -1.65 ± 0.35 | [-2.34, -0.96] | 2.12 | True (W = 0.961, p = 0.718) | False (rho = -0.739, p = 0.002) |
| BioSPPY & Neurokit & Neurokit | easy | 0.20 ± 0.86 | [-1.49, 1.88] | 2.39 | True (W = 0.935, p = 0.320) | True (rho = -0.039, p = 0.889) | -2.61 ± 0.88 | [-4.34, -0.89] | 0.88 | True (W = 0.941, p = 0.394) | True (rho = -0.396, p = 0.143) | -1.62 ± 0.37 | [-2.35, -0.89] | 2.62 | True (W = 0.926, p = 0.240) | True (rho = -0.321, p = 0.243) |
| BioSPPY & Neurokit & Neurokit | hard | 0.18 ± 0.55 | [-0.89, 1.25] | 1.6 | True (W = 0.952, p = 0.559) | True (rho = 0.293, p = 0.289) | -2.83 ± 0.88 | [-4.55, -1.11] | 0.95 | True (W = 0.929, p = 0.263) | False (rho = -0.764, p = 0.001) | -1.65 ± 0.35 | [-2.34, -0.96] | 2.12 | True (W = 0.961, p = 0.718) | False (rho = -0.739, p = 0.002) |
| BioSPPY & Biopac & Kim | easy | 0.09 ± 0.57 | [-1.03, 1.21] | 1.53 | True (W = 0.935, p = 0.463) | True (rho = -0.136, p = 0.689) | -2.57 ± 0.76 | [-4.05, -1.09] | 0.55 | True (W = 0.967, p = 0.857) | False (rho = -0.691, p = 0.019) | -1.76 ± 0.27 | [-2.28, -1.24] | 2.21 | True (W = 0.874, p = 0.087) | True (rho = -0.409, p = 0.212) |
| BioSPPY & Biopac & Kim | hard | 0.12 ± 0.86 | [-1.57, 1.80] | 2.19 | True (W = 0.959, p = 0.811) | True (rho = -0.543, p = 0.266) | nan ± nan | [nan, nan] |  | True (W = 0.897, p = 0.394) | True (rho = nan, p = nan) | -1.59 ± 0.22 | [-2.03, -1.15] | 1.24 | True (W = 0.924, p = 0.534) | True (rho = -0.771, p = 0.072) |
| BioSPPY & Biopac & Neurokit | easy | 0.10 ± 0.70 | [-1.27, 1.47] | 1.6 | True (W = 0.903, p = 0.104) | True (rho = -0.025, p = 0.930) | -2.40 ± 0.74 | [-3.86, -0.95] | 0.64 | True (W = 0.966, p = 0.799) | True (rho = -0.429, p = 0.111) | -1.62 ± 0.37 | [-2.35, -0.89] | 2.62 | True (W = 0.926, p = 0.240) | True (rho = -0.321, p = 0.243) |
| BioSPPY & Biopac & Neurokit | hard | 0.32 ± 0.58 | [-0.81, 1.44] | 1.22 | True (W = 0.931, p = 0.282) | True (rho = -0.311, p = 0.260) | -2.66 ± 0.85 | [-4.33, -1.00] | 0.76 | False (W = 0.856, p = 0.021) | False (rho = -0.557, p = 0.031) | -1.65 ± 0.35 | [-2.34, -0.96] | 2.12 | True (W = 0.962, p = 0.719) | False (rho = -0.739, p = 0.002) |
| Neurokit & Greco & Gamboa | easy | nan ± nan | [nan, nan] |  |  |  | -3.70 ± 1.62 | [-6.88, -0.52] | 1.1 | True (W = 0.973, p = 0.901) | False (rho = -0.596, p = 0.019) | nan ± nan | [nan, nan] |  | True (W = 0.922, p = 0.237) | True (rho = nan, p = nan) |
| Neurokit & Greco & Gamboa | hard | nan ± nan | [nan, nan] |  |  |  | -3.93 ± 1.81 | [-7.47, -0.39] | 1.3 | True (W = 0.959, p = 0.673) | False (rho = -0.821, p = 0.000) | nan ± nan | [nan, nan] |  | True (W = 0.965, p = 0.825) | True (rho = nan, p = nan) |
| Neurokit & Greco & Kim | easy | -0.49 ± 0.75 | [-1.97, 0.98] | 2.55 | True (W = 0.888, p = 0.077) | True (rho = -0.429, p = 0.126) | -3.85 ± 2.43 | [-8.61, 0.91] | 1.72 | True (W = 0.932, p = 0.324) | False (rho = -0.785, p = 0.001) | -1.60 ± 0.38 | [-2.35, -0.85] | 2.91 | True (W = 0.922, p = 0.237) | True (rho = -0.327, p = 0.253) |
| Neurokit & Greco & Kim | hard | -0.81 ± 0.90 | [-2.58, 0.96] | 5.01 | True (W = 0.893, p = 0.075) | True (rho = -0.404, p = 0.136) | nan ± nan | [nan, nan] |  | True (W = 0.951, p = 0.578) | True (rho = nan, p = nan) | nan ± nan | [nan, nan] |  | True (W = 0.965, p = 0.825) | True (rho = nan, p = nan) |
| Neurokit & Greco & Nabian | easy | -0.16 ± 0.47 | [-1.08, 0.76] | 3.26 | True (W = 0.982, p = 0.980) | False (rho = -0.589, p = 0.021) | -3.95 ± 1.87 | [-7.61, -0.28] | 1.61 | True (W = 0.973, p = 0.900) | False (rho = -0.650, p = 0.009) | nan ± nan | [nan, nan] |  | True (W = 0.922, p = 0.237) | True (rho = nan, p = nan) |
| Neurokit & Greco & Nabian | hard | -0.19 ± 0.65 | [-1.46, 1.08] | 8.91 | True (W = 0.931, p = 0.284) | False (rho = -0.718, p = 0.003) | -4.14 ± 2.02 | [-8.10, -0.19] | 1.97 | True (W = 0.946, p = 0.470) | False (rho = -0.789, p = 0.000) | nan ± nan | [nan, nan] |  | True (W = 0.965, p = 0.825) | True (rho = nan, p = nan) |
| Neurokit & Greco & Neurokit | easy | -0.71 ± 1.03 | [-2.73, 1.30] | 141.83 | True (W = 0.968, p = 0.826) | False (rho = -0.782, p = 0.001) | nan ± nan | [nan, nan] |  | True (W = 0.912, p = 0.227) | True (rho = nan, p = nan) | nan ± nan | [nan, nan] |  | True (W = 0.922, p = 0.237) | True (rho = nan, p = nan) |
| Neurokit & Greco & Neurokit | hard | -0.21 ± 0.70 | [-1.58, 1.16] | 10.08 | True (W = 0.951, p = 0.536) | True (rho = -0.346, p = 0.206) | nan ± nan | [nan, nan] |  | True (W = 0.944, p = 0.471) | True (rho = nan, p = nan) | nan ± nan | [nan, nan] |  | True (W = 0.965, p = 0.825) | True (rho = nan, p = nan) |
| Neurokit & Neurokit & Gamboa | easy | nan ± nan | [nan, nan] |  |  |  | -2.34 ± 0.79 | [-3.89, -0.78] | 0.64 | True (W = 0.974, p = 0.912) | True (rho = 0.039, p = 0.889) | -1.62 ± 0.37 | [-2.35, -0.89] | 2.62 | True (W = 0.926, p = 0.240) | True (rho = -0.321, p = 0.243) |
| Neurokit & Neurokit & Gamboa | hard | nan ± nan | [nan, nan] |  |  |  | -2.42 ± 0.83 | [-4.04, -0.80] | 0.69 | True (W = 0.983, p = 0.985) | True (rho = -0.050, p = 0.860) | -1.65 ± 0.35 | [-2.34, -0.96] | 2.12 | True (W = 0.961, p = 0.718) | False (rho = -0.739, p = 0.002) |
| Neurokit & Neurokit & Kim | easy | -0.09 ± 1.21 | [-2.47, 2.28] | 2.87 | True (W = 0.915, p = 0.352) | True (rho = -0.333, p = 0.381) | -2.64 ± 0.87 | [-4.35, -0.93] | 1.05 | True (W = 0.844, p = 0.064) | False (rho = -0.917, p = 0.001) | -1.61 ± 0.40 | [-2.39, -0.83] | 2.26 | False (W = 0.834, p = 0.050) | False (rho = -0.717, p = 0.030) |
| Neurokit & Neurokit & Kim | hard | -0.07 ± 0.57 | [-1.19, 1.04] | 1.37 | True (W = 0.942, p = 0.605) | True (rho = 0.050, p = 0.898) | -2.40 ± 0.82 | [-4.01, -0.79] | 1.06 | True (W = 0.962, p = 0.824) | False (rho = -0.883, p = 0.002) | -1.58 ± 0.34 | [-2.25, -0.91] | 1.9 | True (W = 0.934, p = 0.520) | True (rho = -0.383, p = 0.308) |
| Neurokit & Neurokit & Nabian | easy | 0.50 ± 0.33 | [-0.15, 1.15] | 0.6 | True (W = 0.933, p = 0.542) | True (rho = 0.119, p = 0.779) | -2.53 ± 0.84 | [-4.18, -0.88] | 0.92 | True (W = 0.941, p = 0.620) | False (rho = -0.833, p = 0.010) | -1.60 ± 0.42 | [-2.42, -0.78] | 2.42 | True (W = 0.835, p = 0.066) | False (rho = -0.738, p = 0.037) |
| Neurokit & Neurokit & Nabian | hard | 0.42 ± 0.39 | [-0.34, 1.17] | 0.67 | True (W = 0.896, p = 0.196) | True (rho = -0.115, p = 0.751) | -2.58 ± 0.82 | [-4.18, -0.97] | 0.88 | True (W = 0.956, p = 0.738) | True (rho = -0.467, p = 0.174) | -1.64 ± 0.37 | [-2.37, -0.91] | 2.16 | True (W = 0.935, p = 0.503) | False (rho = -0.855, p = 0.002) |
| Neurokit & Neurokit & Neurokit | easy | nan ± nan | [nan, nan] |  |  |  | -2.42 ± 0.65 | [-3.69, -1.14] | 0.64 | True (W = 0.927, p = 0.242) | True (rho = -0.161, p = 0.567) | -1.62 ± 0.37 | [-2.35, -0.89] | 2.62 | True (W = 0.926, p = 0.240) | True (rho = -0.321, p = 0.243) |
| Neurokit & Neurokit & Neurokit | hard | nan ± nan | [nan, nan] |  |  |  | -2.77 ± 0.87 | [-4.47, -1.06] | 0.94 | True (W = 0.953, p = 0.571) | True (rho = -0.414, p = 0.125) | -1.65 ± 0.35 | [-2.34, -0.96] | 2.12 | True (W = 0.961, p = 0.718) | False (rho = -0.739, p = 0.002) |
| Neurokit & Biopac & Neurokit | easy | nan ± nan | [nan, nan] |  |  |  | -2.30 ± 0.70 | [-3.67, -0.93] | 0.63 | True (W = 0.956, p = 0.622) | True (rho = -0.039, p = 0.889) | -1.62 ± 0.37 | [-2.35, -0.89] | 2.62 | True (W = 0.925, p = 0.233) | True (rho = -0.321, p = 0.243) |
| Neurokit & Biopac & Neurokit | hard | nan ± nan | [nan, nan] |  |  |  | -2.52 ± 0.84 | [-4.16, -0.88] | 0.79 | True (W = 0.960, p = 0.695) | True (rho = -0.346, p = 0.206) | -1.65 ± 0.35 | [-2.34, -0.97] | 2.12 | True (W = 0.961, p = 0.711) | False (rho = -0.739, p = 0.002) |

*Note*. The BAr results and corresponding values for all EDA parameters of interest across tested pipelines. The pipeline annotation consists of “data cleaning method & signal decomposition method & peak detection method”. The pipeline configuration used for creating the Bland-Altman plots is marked with an asterisk. Note that one configuration yielded no SCR amplitude values.

Table 5
*BAr Results for EDA Parameters Across Processing Pipelines*

| Processing pipeline | Workload condition | SCR | | | | | | SCR Amplitude | | | | | | SCL | | | | | |
| --- | --- | --- | --- | --- | --- | --- | --- | --- | --- | --- | --- | --- | --- | --- | --- | --- | --- | --- | --- |
|  |  | Mean ± SD | 95% CI | BAr | Within boundaries (%) | normality | homoscedasticity | Mean ± SD | 95% CI | BAr | Within boundaries (%) | normality | homoscedasticity | Mean ± SD | 95% CI | BAr | Within boundaries (%) | normality | homoscedasticity |
| BioSPPY & Greco & Gamboa | easy | -12.07 ± 9.96 | [-31.58, 7.45] | 1.69 | 0 | False (W = 0.847, p = 0.016) | False (rho = 0.739, p = 0.002) | -0.11 ± 0.08 | [-0.27, 0.05] | 2.78 | 100 | True (W = 0.930, p = 0.271) | False (rho = 1.000, p = 0.000) | -12.32 ± 3.76 | [-19.68, -4.96] | 1.12 | 0 | True (W = 0.893, p = 0.075) | False (rho = 0.918, p = 0.000) |
| BioSPPY & Greco & Gamboa | hard | -13.34 ± 12.92 | [-38.66, 11.99] | 2.06 | 13.33 | False (W = 0.770, p = 0.002) | False (rho = 0.846, p = 0.000) | -0.16 ± 0.10 | [-0.35, 0.02] | 2.21 | 100 | True (W = 0.887, p = 0.061) | False (rho = 0.996, p = 0.000) | -13.57 ± 3.14 | [-19.73, -7.41] | 0.85 | 0 | True (W = 0.981, p = 0.976) | False (rho = 0.889, p = 0.000) |
| BioSPPY & Greco & Kim | easy | -1.71 ± 4.07 | [-9.68, 6.27] | 1.85 | 40 | True (W = 0.911, p = 0.142) | True (rho = -0.214, p = 0.443) | -0.30 ± 0.16 | [-0.62, 0.02] | 2.07 | 100 | True (W = 0.903, p = 0.107) | False (rho = 0.943, p = 0.000) | -12.32 ± 3.76 | [-19.68, -4.96] | 1.12 | 0 | True (W = 0.893, p = 0.075) | False (rho = 0.918, p = 0.000) |
| BioSPPY & Greco & Kim | hard | -3.55 ± 6.42 | [-16.13, 9.02] | 2.66 | 50 | False (W = 0.708, p = 0.000) | True (rho = 0.499, p = 0.069) | -1.17 ± 2.51 | [-6.10, 3.75] | 8.32 | 78.57 | False (W = 0.427, p = 0.000) | False (rho = 1.000, p = 0.000) | -13.83 ± 3.09 | [-19.88, -7.78] | 0.83 | 0 | True (W = 0.964, p = 0.791) | False (rho = 0.864, p = 0.000) |
| BioSPPY & Greco & Nabian * | easy | -1.60 ± 1.82 | [-5.17, 1.96] | 1.24 | 66.67 | True (W = 0.978, p = 0.956) | True (rho = 0.336, p = 0.221) | -0.44 ± 0.28 | [-0.99, 0.12] | 2.48 | 73.33 | True (W = 0.970, p = 0.856) | False (rho = 0.989, p = 0.000) | -12.32 ± 3.76 | [-19.68, -4.96] | 1.12 | 0 | True (W = 0.893, p = 0.075) | False (rho = 0.918, p = 0.000) |
| BioSPPY & Greco & Nabian * | hard | -1.31 ± 1.53 | [-4.30, 1.68] | 1.14 | 80 | True (W = 0.982, p = 0.984) | True (rho = 0.157, p = 0.576) | -0.76 ± 0.51 | [-1.77, 0.24] | 2.61 | 46.67 | False (W = 0.857, p = 0.022) | False (rho = 0.989, p = 0.000) | -13.57 ± 3.14 | [-19.73, -7.41] | 0.85 | 0 | True (W = 0.981, p = 0.976) | False (rho = 0.889, p = 0.000) |
| BioSPPY & Greco & Neurokit | easy | -0.84 ± 1.57 | [-3.91, 2.24] | 1.56 | 93.33 | True (W = 0.970, p = 0.859) | True (rho = 0.118, p = 0.676) | nan ± nan | [nan, nan] |  | 60 | True (W = 0.930, p = 0.339) | True (rho = nan, p = nan) | -12.32 ± 3.76 | [-19.68, -4.96] | 1.12 | 0 | True (W = 0.893, p = 0.075) | False (rho = 0.918, p = 0.000) |
| BioSPPY & Greco & Neurokit | hard | -0.52 ± 1.70 | [-3.86, 2.82] | 1.74 | 93.33 | True (W = 0.977, p = 0.942) | True (rho = 0.021, p = 0.940) | nan ± nan | [nan, nan] |  | 46.67 | False (W = 0.586, p = 0.000) | True (rho = nan, p = nan) | -13.57 ± 3.14 | [-19.73, -7.41] | 0.85 | 0 | True (W = 0.981, p = 0.976) | False (rho = 0.889, p = 0.000) |
| BioSPPY & Neurokit & Gamboa | easy | nan ± nan | [nan, nan] |  |  |  |  | -0.08 ± 0.07 | [-0.21, 0.05] | 3.16 | 100 | False (W = 0.877, p = 0.043) | False (rho = 1.000, p = 0.000) | -12.58 ± 3.78 | [-19.99, -5.17] | 1.1 | 0 | False (W = 0.877, p = 0.043) | False (rho = 0.918, p = 0.000) |
| BioSPPY & Neurokit & Gamboa | hard | nan ± nan | [nan, nan] |  |  |  |  | -0.10 ± 0.06 | [-0.23, 0.02] | 2.33 | 100 | True (W = 0.900, p = 0.094) | False (rho = 1.000, p = 0.000) | -14.01 ± 3.19 | [-20.26, -7.76] | 0.84 | 0 | True (W = 0.972, p = 0.883) | False (rho = 0.918, p = 0.000) |
| BioSPPY & Neurokit & Kim | easy | 7.29 ± 12.25 | [-16.72, 31.30] | 2.78 | 33.33 | False (W = 0.860, p = 0.024) | False (rho = 0.743, p = 0.002) | -0.33 ± 0.19 | [-0.71, 0.05] | 2.22 | 93.33 | True (W = 0.912, p = 0.146) | False (rho = 0.943, p = 0.000) | -12.58 ± 3.78 | [-19.99, -5.17] | 1.1 | 0 | False (W = 0.877, p = 0.043) | False (rho = 0.918, p = 0.000) |
| BioSPPY & Neurokit & Kim | hard | 5.45 ± 10.17 | [-14.48, 25.38] | 2.53 | 26.67 | True (W = 0.920, p = 0.190) | False (rho = 0.836, p = 0.000) | -0.56 ± 0.39 | [-1.32, 0.20] | 2.68 | 66.67 | True (W = 0.896, p = 0.082) | False (rho = 0.993, p = 0.000) | -14.01 ± 3.19 | [-20.26, -7.76] | 0.84 | 0 | True (W = 0.972, p = 0.883) | False (rho = 0.918, p = 0.000) |
| BioSPPY & Neurokit & Nabian | easy | 9.83 ± 12.33 | [-14.34, 33.99] | 1.69 | 13.33 | True (W = 0.917, p = 0.175) | True (rho = 0.246, p = 0.376) | -0.25 ± 0.15 | [-0.55, 0.05] | 2.33 | 100 | True (W = 0.933, p = 0.306) | False (rho = 0.986, p = 0.000) | -12.58 ± 3.78 | [-19.99, -5.17] | 1.1 | 0 | False (W = 0.877, p = 0.043) | False (rho = 0.918, p = 0.000) |
| BioSPPY & Neurokit & Nabian | hard | 7.37 ± 10.85 | [-13.90, 28.63] | 1.72 | 13.33 | True (W = 0.963, p = 0.739) | False (rho = 0.650, p = 0.009) | -0.35 ± 0.29 | [-0.92, 0.21] | 3.14 | 86.67 | False (W = 0.780, p = 0.002) | False (rho = 0.989, p = 0.000) | -14.01 ± 3.19 | [-20.26, -7.76] | 0.84 | 0 | True (W = 0.972, p = 0.883) | False (rho = 0.918, p = 0.000) |
| BioSPPY & Neurokit & Neurokit | easy | 7.65 ± 13.11 | [-18.05, 33.35] | 2.79 | 33.33 | False (W = 0.851, p = 0.018) | False (rho = 0.686, p = 0.005) | -0.32 ± 0.20 | [-0.71, 0.07] | 2.35 | 93.33 | True (W = 0.944, p = 0.439) | False (rho = 0.979, p = 0.000) | -12.58 ± 3.78 | [-19.99, -5.17] | 1.1 | 0 | False (W = 0.877, p = 0.043) | False (rho = 0.918, p = 0.000) |
| BioSPPY & Neurokit & Neurokit | hard | 5.24 ± 12.34 | [-18.95, 29.44] | 2.78 | 33.33 | True (W = 0.884, p = 0.054) | False (rho = 0.861, p = 0.000) | -0.50 ± 0.35 | [-1.19, 0.19] | 2.7 | 80 | False (W = 0.865, p = 0.029) | False (rho = 0.993, p = 0.000) | -14.01 ± 3.19 | [-20.26, -7.76] | 0.84 | 0 | True (W = 0.972, p = 0.883) | False (rho = 0.918, p = 0.000) |
| BioSPPY & Biopac & Kim | easy | 4.50 ± 9.86 | [-14.82, 23.82] | 2.11 | 27.27 | True (W = 0.960, p = 0.777) | False (rho = 0.773, p = 0.005) | -0.06 ± 0.06 | [-0.17, 0.05] | 3.89 | 100 | False (W = 0.745, p = 0.002) | False (rho = 1.000, p = 0.000) | -13.42 ± 3.43 | [-20.14, -6.70] | 0.97 | 0 | True (W = 0.860, p = 0.058) | False (rho = 0.991, p = 0.000) |
| BioSPPY & Biopac & Kim | hard | 2.92 ± 14.88 | [-26.26, 32.09] | 2.8 | 16.67 | True (W = 0.952, p = 0.757) | False (rho = 0.829, p = 0.042) | -0.10 ± 0.08 | [-0.27, 0.06] | 3.23 | 100 | True (W = 0.883, p = 0.284) | False (rho = 1.000, p = 0.000) | -13.94 ± 2.29 | [-18.44, -9.44] | 0.61 | 0 | True (W = 0.937, p = 0.634) | False (rho = 1.000, p = 0.000) |
| BioSPPY & Biopac & Neurokit | easy | 5.79 ± 12.47 | [-18.65, 30.22] | 2.17 | 20 | True (W = 0.951, p = 0.547) | False (rho = 0.779, p = 0.001) | -0.11 ± 0.08 | [-0.27, 0.05] | 2.71 | 100 | True (W = 0.907, p = 0.122) | False (rho = 0.996, p = 0.000) | -12.58 ± 3.78 | [-19.98, -5.17] | 1.1 | 0 | False (W = 0.877, p = 0.043) | False (rho = 0.918, p = 0.000) |
| BioSPPY & Biopac & Neurokit | hard | 7.22 ± 12.24 | [-16.77, 31.21] | 2.01 | 13.33 | False (W = 0.869, p = 0.033) | False (rho = 0.707, p = 0.003) | -0.16 ± 0.10 | [-0.37, 0.04] | 2.41 | 100 | False (W = 0.869, p = 0.033) | False (rho = 0.996, p = 0.000) | -14.01 ± 3.19 | [-20.26, -7.76] | 0.84 | 0 | True (W = 0.972, p = 0.881) | False (rho = 0.918, p = 0.000) |
| Neurokit & Greco & Gamboa | easy | nan ± nan | [nan, nan] |  |  |  |  | -0.29 ± 0.66 | [-1.58, 1.00] | 8.86 | 93.33 | False (W = 0.421, p = 0.000) | False (rho = 1.000, p = 0.000) | -4.29 ± 28.94 | [-61.02, 52.43] | 22.07 | 0 | False (W = 0.401, p = 0.000) | False (rho = 0.586, p = 0.022) |
| Neurokit & Greco & Gamboa | hard | nan ± nan | [nan, nan] |  |  |  |  | -0.31 ± 0.54 | [-1.36, 0.74] | 6.72 | 86.67 | False (W = 0.469, p = 0.000) | False (rho = 1.000, p = 0.000) | -5.30 ± 27.50 | [-59.20, 48.59] | 17.53 | 6.67 | False (W = 0.434, p = 0.000) | False (rho = 0.543, p = 0.037) |
| Neurokit & Greco & Kim | easy | -6.62 ± 12.61 | [-31.32, 18.09] | 3.46 | 35.71 | False (W = 0.731, p = 0.001) | True (rho = 0.407, p = 0.149) | -0.29 ± 0.19 | [-0.66, 0.09] | 2.53 | 92.86 | True (W = 0.949, p = 0.547) | False (rho = 0.974, p = 0.000) | -11.70 ± 3.86 | [-19.28, -4.13] | 1.21 | 0 | True (W = 0.947, p = 0.512) | False (rho = 0.952, p = 0.000) |
| Neurokit & Greco & Kim | hard | -12.31 ± 20.60 | [-52.69, 28.08] | 4.5 | 40 | False (W = 0.715, p = 0.000) | False (rho = 0.929, p = 0.000) | -0.92 ± 2.73 | [-6.28, 4.44] | 11.56 | 66.67 | False (W = 0.611, p = 0.000) | False (rho = 0.664, p = 0.007) | -5.30 ± 27.50 | [-59.20, 48.59] | 17.53 | 6.67 | False (W = 0.434, p = 0.000) | False (rho = 0.543, p = 0.037) |
| Neurokit & Greco & Nabian | easy | -0.87 ± 1.91 | [-4.62, 2.89] | 1.43 | 80 | True (W = 0.945, p = 0.451) | True (rho = 0.386, p = 0.156) | -1.54 ± 2.76 | [-6.94, 3.87] | 7 | 53.33 | False (W = 0.569, p = 0.000) | False (rho = 0.996, p = 0.000) | -4.29 ± 28.94 | [-61.02, 52.43] | 22.07 | 0 | False (W = 0.401, p = 0.000) | False (rho = 0.586, p = 0.022) |
| Neurokit & Greco & Nabian | hard | -0.40 ± 1.63 | [-3.60, 2.81] | 1.5 | 93.33 | True (W = 0.972, p = 0.882) | True (rho = 0.239, p = 0.390) | -2.84 ± 6.24 | [-15.07, 9.38] | 8.57 | 26.67 | False (W = 0.421, p = 0.000) | False (rho = 0.975, p = 0.000) | -5.30 ± 27.50 | [-59.20, 48.59] | 17.53 | 6.67 | False (W = 0.434, p = 0.000) | False (rho = 0.543, p = 0.037) |
| Neurokit & Greco & Neurokit | easy | -4.06 ± 12.95 | [-29.44, 21.32] | 7.31 | 86.67 | False (W = 0.404, p = 0.000) | True (rho = 0.196, p = 0.483) | nan ± nan | [nan, nan] |  | 66.67 | False (W = 0.567, p = 0.000) | True (rho = nan, p = nan) | -4.29 ± 28.94 | [-61.02, 52.43] | 22.07 | 0 | False (W = 0.401, p = 0.000) | False (rho = 0.586, p = 0.022) |
| Neurokit & Greco & Neurokit | hard | 0.11 ± 1.76 | [-3.35, 3.57] | 2.19 | 86.67 | True (W = 0.953, p = 0.570) | False (rho = 0.679, p = 0.005) | nan ± nan | [nan, nan] |  | 53.33 | False (W = 0.596, p = 0.000) | True (rho = nan, p = nan) | -5.30 ± 27.50 | [-59.20, 48.59] | 17.53 | 6.67 | False (W = 0.434, p = 0.000) | False (rho = 0.543, p = 0.037) |
| Neurokit & Neurokit & Gamboa | easy | nan ± nan | [nan, nan] |  |  |  |  | -0.18 ± 0.46 | [-1.09, 0.72] | 9.81 | 93.33 | False (W = 0.389, p = 0.000) | False (rho = 1.000, p = 0.000) | -12.58 ± 3.78 | [-19.99, -5.17] | 1.1 | 0 | False (W = 0.877, p = 0.043) | False (rho = 0.918, p = 0.000) |
| Neurokit & Neurokit & Gamboa | hard | nan ± nan | [nan, nan] |  |  |  |  | -0.22 ± 0.48 | [-1.16, 0.73] | 8.64 | 93.33 | False (W = 0.443, p = 0.000) | False (rho = 0.996, p = 0.000) | -14.01 ± 3.19 | [-20.26, -7.76] | 0.84 | 0 | True (W = 0.972, p = 0.883) | False (rho = 0.918, p = 0.000) |
| Neurokit & Neurokit & Kim | easy | 3.20 ± 29.72 | [-55.05, 61.44] | 3.43 | 22.22 | True (W = 0.895, p = 0.225) | False (rho = 0.717, p = 0.030) | -0.76 ± 1.08 | [-2.88, 1.37] | 5.49 | 88.89 | False (W = 0.469, p = 0.000) | False (rho = 0.817, p = 0.007) | -13.73 ± 2.41 | [-18.47, -9.00] | 0.64 | 0 | True (W = 0.979, p = 0.959) | False (rho = 0.833, p = 0.005) |
| Neurokit & Neurokit & Kim | hard | -0.27 ± 17.26 | [-34.09, 33.56] | 2.41 | 44.44 | True (W = 0.906, p = 0.290) | False (rho = 0.900, p = 0.001) | -0.52 ± 0.27 | [-1.04, 0.00] | 1.96 | 66.67 | True (W = 0.904, p = 0.277) | False (rho = 0.883, p = 0.002) | -13.81 ± 3.70 | [-21.06, -6.56] | 0.98 | 0 | True (W = 0.944, p = 0.624) | False (rho = 0.900, p = 0.001) |
| Neurokit & Neurokit & Nabian | easy | 16.35 ± 10.98 | [-5.16, 37.87] | 1.32 | 12.5 | True (W = 0.960, p = 0.809) | False (rho = 0.881, p = 0.004) | -0.31 ± 0.09 | [-0.49, -0.13] | 1.11 | 100 | True (W = 0.917, p = 0.408) | False (rho = 0.929, p = 0.001) | -13.40 ± 2.52 | [-18.35, -8.46] | 0.68 | 0 | True (W = 0.944, p = 0.648) | False (rho = 0.857, p = 0.007) |
| Neurokit & Neurokit & Nabian | hard | 14.15 ± 12.55 | [-10.46, 38.75] | 1.45 | 30 | True (W = 0.893, p = 0.185) | True (rho = 0.564, p = 0.090) | -0.36 ± 0.30 | [-0.95, 0.22] | 3.13 | 90 | False (W = 0.719, p = 0.001) | False (rho = 0.952, p = 0.000) | -14.20 ± 2.96 | [-20.01, -8.39] | 0.77 | 0 | True (W = 0.918, p = 0.338) | False (rho = 0.818, p = 0.004) |
| Neurokit & Neurokit & Neurokit | easy | nan ± nan | [nan, nan] |  |  |  |  | -0.34 ± 0.69 | [-1.70, 1.02] | 7.72 | 93.33 | False (W = 0.401, p = 0.000) | False (rho = 1.000, p = 0.000) | -12.58 ± 3.78 | [-19.99, -5.17] | 1.1 | 0 | False (W = 0.877, p = 0.043) | False (rho = 0.918, p = 0.000) |
| Neurokit & Neurokit & Neurokit | hard | nan ± nan | [nan, nan] |  |  |  |  | -0.57 ± 0.77 | [-2.08, 0.95] | 5.27 | 73.33 | False (W = 0.560, p = 0.000) | False (rho = 0.925, p = 0.000) | -14.01 ± 3.19 | [-20.26, -7.76] | 0.84 | 0 | True (W = 0.972, p = 0.883) | False (rho = 0.918, p = 0.000) |
| Neurokit & Biopac & Neurokit | easy | nan ± nan | [nan, nan] |  |  |  |  | -0.28 ± 0.75 | [-1.75, 1.18] | 10.11 | 93.33 | False (W = 0.360, p = 0.000) | False (rho = 0.993, p = 0.000) | -12.61 ± 3.79 | [-20.04, -5.19] | 1.1 | 0 | False (W = 0.875, p = 0.040) | False (rho = 0.918, p = 0.000) |
| Neurokit & Biopac & Neurokit | hard | nan ± nan | [nan, nan] |  |  |  |  | -0.37 ± 0.81 | [-1.95, 1.22] | 8.52 | 93.33 | False (W = 0.408, p = 0.000) | False (rho = 0.979, p = 0.000) | -14.04 ± 3.19 | [-20.28, -7.79] | 0.84 | 0 | True (W = 0.970, p = 0.858) | False (rho = 0.914, p = 0.000) |

*Note*. The BAr results and corresponding values for all EDA parameters of interest across tested pipelines. The pipeline annotation consists of “data cleaning method & signal decomposition method & peak detection method”. The pipeline configuration used for creating the Bland-Altman plots is marked with an asterisk. Note that not all processing configurations were compatible.

Table 6
*BAr Results for Log-Transformed CVA Parameters Across Processing Pipelines*

| Processing pipeline | Workload condition | Log(HR) | | | | | Log(SD RR/PP interval) | | | | | Log(RMSSD) | | | | |
| --- | --- | --- | --- | --- | --- | --- | --- | --- | --- | --- | --- | --- | --- | --- | --- | --- |
|  |  | Mean ± SD | 95% CI | BAr | normality | homoscedasticity | Mean ± SD | 95% CI | BAr | normality | homoscedasticity | Mean ± SD | 95% CI | BAr | normality | homoscedasticity |
| ECG BioSPPY & Elgendi; PPG Elgendi & Elgendi | easy | 0.02 ± 0.06 | [-0.09, 0.14] | 0.06 | False (W = 0.425, p = 0.000) | False (rho = 0.668, p = 0.007) | -0.05 ± 0.29 | [-0.62, 0.53] | 0.29 | True (W = 0.945, p = 0.455) | True (rho = -0.343, p = 0.211) | -0.18 ± 0.36 | [-0.90, 0.53] | 0.37 | True (W = 0.897, p = 0.085) | True (rho = -0.321, p = 0.243) |
| ECG BioSPPY & Elgendi; PPG Elgendi & Elgendi | hard | 0.01 ± 0.02 | [-0.03, 0.05] | 0.02 | False (W = 0.636, p = 0.000) | True (rho = 0.471, p = 0.076) | -0.06 ± 0.30 | [-0.65, 0.53] | 0.31 | True (W = 0.938, p = 0.362) | True (rho = -0.207, p = 0.459) | -0.23 ± 0.42 | [-1.05, 0.58] | 0.43 | True (W = 0.937, p = 0.342) | True (rho = -0.314, p = 0.254) |
| ECG BioSPPY & Elgendi; PPG Langevin & Elgendi | easy | 0.02 ± 0.06 | [-0.09, 0.14] | 0.06 | False (W = 0.448, p = 0.000) | False (rho = 0.600, p = 0.018) | 0.03 ± 0.25 | [-0.46, 0.53] | 0.26 | True (W = 0.925, p = 0.228) | True (rho = 0.168, p = 0.550) | -0.07 ± 0.29 | [-0.65, 0.50] | 0.3 | True (W = 0.975, p = 0.922) | True (rho = -0.004, p = 0.990) |
| ECG BioSPPY & Elgendi; PPG Langevin & Elgendi | hard | 0.01 ± 0.02 | [-0.04, 0.05] | 0.02 | False (W = 0.599, p = 0.000) | True (rho = 0.014, p = 0.960) | 0.00 ± 0.28 | [-0.55, 0.55] | 0.29 | True (W = 0.936, p = 0.331) | True (rho = 0.004, p = 0.990) | -0.11 ± 0.38 | [-0.86, 0.64] | 0.4 | True (W = 0.918, p = 0.178) | True (rho = -0.068, p = 0.810) |
| ECG BioSPPY & Nabian; PPG Elgendi & Elgendi | easy | 0.00 ± 0.00 | [-0.01, 0.01] | 0 | True (W = 0.971, p = 0.874) | True (rho = 0.429, p = 0.111) | -0.22 ± 0.27 | [-0.75, 0.30] | 0.28 | True (W = 0.926, p = 0.238) | True (rho = -0.304, p = 0.271) | -0.40 ± 0.39 | [-1.16, 0.36] | 0.41 | True (W = 0.965, p = 0.780) | True (rho = -0.346, p = 0.206) |
| ECG BioSPPY & Nabian; PPG Elgendi & Elgendi | hard | -0.00 ± 0.01 | [-0.01, 0.01] | 0.01 | True (W = 0.900, p = 0.094) | True (rho = 0.139, p = 0.621) | -0.18 ± 0.21 | [-0.60, 0.23] | 0.23 | False (W = 0.865, p = 0.029) | True (rho = -0.139, p = 0.621) | -0.38 ± 0.36 | [-1.08, 0.32] | 0.39 | True (W = 0.940, p = 0.383) | True (rho = -0.400, p = 0.140) |
| ECG BioSPPY & Nabian; PPG Langevin & Elgendi | easy | -0.00 ± 0.00 | [-0.01, 0.01] | 0 | True (W = 0.929, p = 0.261) | True (rho = 0.489, p = 0.064) | -0.13 ± 0.20 | [-0.52, 0.27] | 0.21 | True (W = 0.972, p = 0.888) | True (rho = -0.304, p = 0.271) | -0.25 ± 0.32 | [-0.88, 0.37] | 0.35 | True (W = 0.973, p = 0.895) | True (rho = 0.004, p = 0.990) |
| ECG BioSPPY & Nabian; PPG Langevin & Elgendi | hard | -0.00 ± 0.01 | [-0.01, 0.01] | 0.01 | False (W = 0.737, p = 0.001) | True (rho = -0.207, p = 0.459) | -0.12 ± 0.19 | [-0.49, 0.24] | 0.2 | False (W = 0.867, p = 0.030) | True (rho = 0.014, p = 0.960) | -0.28 ± 0.36 | [-0.99, 0.43] | 0.4 | True (W = 0.920, p = 0.193) | True (rho = 0.150, p = 0.594) |
| ECG BioSPPY & Neurokit; PPG Elgendi & Elgendi | easy | 0.00 ± 0.00 | [-0.01, 0.01] | 0 | True (W = 0.893, p = 0.074) | True (rho = 0.014, p = 0.960) | -0.24 ± 0.21 | [-0.64, 0.17] | 0.22 | True (W = 0.944, p = 0.441) | True (rho = -0.100, p = 0.723) | -0.45 ± 0.29 | [-1.02, 0.13] | 0.32 | True (W = 0.955, p = 0.610) | True (rho = -0.086, p = 0.761) |
| ECG BioSPPY & Neurokit; PPG Elgendi & Elgendi | hard | -0.00 ± 0.01 | [-0.01, 0.01] | 0.01 | True (W = 0.928, p = 0.257) | True (rho = 0.407, p = 0.132) | -0.15 ± 0.26 | [-0.65, 0.35] | 0.27 | True (W = 0.929, p = 0.262) | True (rho = 0.014, p = 0.960) | -0.32 ± 0.37 | [-1.05, 0.41] | 0.4 | True (W = 0.944, p = 0.436) | True (rho = -0.018, p = 0.950) |
| ECG BioSPPY & Neurokit; PPG Langevin & Elgendi | easy | -0.00 ± 0.00 | [-0.01, 0.01] | 0 | False (W = 0.813, p = 0.005) | True (rho = 0.196, p = 0.483) | -0.16 ± 0.16 | [-0.47, 0.15] | 0.17 | True (W = 0.931, p = 0.280) | True (rho = -0.229, p = 0.413) | -0.34 ± 0.25 | [-0.83, 0.16] | 0.28 | True (W = 0.932, p = 0.288) | True (rho = -0.104, p = 0.713) |
| ECG BioSPPY & Neurokit; PPG Langevin & Elgendi | hard | -0.00 ± 0.01 | [-0.01, 0.01] | 0.01 | False (W = 0.798, p = 0.003) | True (rho = -0.129, p = 0.648) | -0.09 ± 0.25 | [-0.57, 0.39] | 0.26 | True (W = 0.889, p = 0.065) | True (rho = 0.289, p = 0.296) | -0.22 ± 0.39 | [-0.98, 0.54] | 0.42 | True (W = 0.929, p = 0.266) | True (rho = 0.179, p = 0.524) |
| ECG Elgendi & Elgendi; PPG Elgendi & Elgendi | easy | 0.00 ± 0.01 | [-0.01, 0.02] | 0.01 | False (W = 0.714, p = 0.000) | True (rho = -0.032, p = 0.909) | -0.23 ± 0.21 | [-0.64, 0.18] | 0.22 | True (W = 0.963, p = 0.739) | True (rho = -0.307, p = 0.265) | -0.40 ± 0.31 | [-1.00, 0.21] | 0.32 | True (W = 0.965, p = 0.776) | True (rho = 0.014, p = 0.960) |
| ECG Elgendi & Elgendi; PPG Elgendi & Elgendi | hard | 0.00 ± 0.01 | [-0.01, 0.01] | 0.01 | True (W = 0.883, p = 0.063) | True (rho = 0.152, p = 0.605) | -0.19 ± 0.22 | [-0.62, 0.25] | 0.24 | True (W = 0.908, p = 0.147) | True (rho = 0.033, p = 0.911) | -0.37 ± 0.37 | [-1.10, 0.36] | 0.4 | True (W = 0.969, p = 0.857) | True (rho = -0.121, p = 0.681) |
| ECG Elgendi & Elgendi; PPG Langevin & Elgendi | easy | 0.00 ± 0.01 | [-0.01, 0.02] | 0.01 | False (W = 0.689, p = 0.000) | True (rho = -0.100, p = 0.723) | -0.14 ± 0.14 | [-0.42, 0.15] | 0.15 | True (W = 0.937, p = 0.352) | True (rho = -0.379, p = 0.164) | -0.27 ± 0.25 | [-0.76, 0.22] | 0.27 | True (W = 0.942, p = 0.402) | True (rho = -0.007, p = 0.980) |
| ECG Elgendi & Elgendi; PPG Langevin & Elgendi | hard | -0.00 ± 0.01 | [-0.01, 0.01] | 0.01 | False (W = 0.605, p = 0.000) | True (rho = -0.099, p = 0.737) | -0.13 ± 0.20 | [-0.52, 0.27] | 0.22 | True (W = 0.901, p = 0.115) | True (rho = 0.200, p = 0.493) | -0.27 ± 0.39 | [-1.04, 0.50] | 0.44 | True (W = 0.978, p = 0.965) | True (rho = -0.042, p = 0.887) |
| ECG Elgendi & Nabian; PPG Elgendi & Elgendi | easy | 0.00 ± 0.00 | [-0.01, 0.01] | 0 | True (W = 0.981, p = 0.976) | True (rho = 0.068, p = 0.810) | -0.27 ± 0.23 | [-0.72, 0.18] | 0.24 | True (W = 0.932, p = 0.295) | True (rho = -0.221, p = 0.428) | -0.48 ± 0.33 | [-1.12, 0.16] | 0.35 | True (W = 0.946, p = 0.465) | True (rho = 0.100, p = 0.723) |
| ECG Elgendi & Nabian; PPG Elgendi & Elgendi | hard | 0.00 ± 0.01 | [-0.01, 0.01] | 0.01 | False (W = 0.870, p = 0.034) | True (rho = 0.225, p = 0.420) | -0.21 ± 0.20 | [-0.60, 0.17] | 0.21 | True (W = 0.894, p = 0.076) | True (rho = 0.025, p = 0.930) | -0.47 ± 0.31 | [-1.09, 0.14] | 0.35 | True (W = 0.984, p = 0.989) | True (rho = 0.104, p = 0.713) |
| ECG Elgendi & Nabian; PPG Langevin & Elgendi * | easy | -0.00 ± 0.00 | [-0.01, 0.01] | 0 | False (W = 0.858, p = 0.023) | True (rho = 0.286, p = 0.302) | -0.18 ± 0.16 | [-0.48, 0.13] | 0.17 | True (W = 0.943, p = 0.416) | True (rho = -0.246, p = 0.376) | -0.35 ± 0.26 | [-0.86, 0.16] | 0.29 | True (W = 0.951, p = 0.540) | True (rho = 0.211, p = 0.451) |
| ECG Elgendi & Nabian; PPG Langevin & Elgendi * | hard | -0.00 ± 0.00 | [-0.01, 0.01] | 0.01 | False (W = 0.606, p = 0.000) | True (rho = -0.307, p = 0.265) | -0.16 ± 0.17 | [-0.50, 0.18] | 0.19 | True (W = 0.887, p = 0.060) | True (rho = 0.307, p = 0.265) | -0.37 ± 0.32 | [-0.99, 0.26] | 0.36 | True (W = 0.981, p = 0.975) | True (rho = 0.386, p = 0.156) |
| ECG Elgendi & Neurokit; PPG Elgendi & Elgendi | easy | 0.00 ± 0.00 | [-0.01, 0.01] | 0 | True (W = 0.929, p = 0.263) | True (rho = 0.346, p = 0.206) | -0.23 ± 0.21 | [-0.65, 0.18] | 0.22 | True (W = 0.954, p = 0.592) | True (rho = -0.093, p = 0.742) | -0.39 ± 0.32 | [-1.02, 0.23] | 0.34 | True (W = 0.908, p = 0.125) | True (rho = 0.132, p = 0.639) |
| ECG Elgendi & Neurokit; PPG Elgendi & Elgendi | hard | 0.00 ± 0.01 | [-0.01, 0.01] | 0.01 | False (W = 0.881, p = 0.050) | True (rho = 0.304, p = 0.271) | -0.20 ± 0.20 | [-0.60, 0.20] | 0.22 | False (W = 0.872, p = 0.036) | True (rho = 0.089, p = 0.752) | -0.40 ± 0.34 | [-1.07, 0.27] | 0.37 | True (W = 0.960, p = 0.688) | True (rho = 0.136, p = 0.630) |
| ECG Elgendi & Neurokit; PPG Langevin & Elgendi | easy | -0.00 ± 0.00 | [-0.01, 0.01] | 0 | False (W = 0.835, p = 0.011) | True (rho = 0.382, p = 0.160) | -0.15 ± 0.16 | [-0.46, 0.16] | 0.17 | True (W = 0.924, p = 0.222) | True (rho = 0.011, p = 0.970) | -0.29 ± 0.28 | [-0.84, 0.27] | 0.31 | True (W = 0.937, p = 0.345) | True (rho = 0.046, p = 0.869) |
| ECG Elgendi & Neurokit; PPG Langevin & Elgendi | hard | -0.00 ± 0.00 | [-0.01, 0.01] | 0.01 | False (W = 0.644, p = 0.000) | True (rho = -0.375, p = 0.168) | -0.14 ± 0.18 | [-0.50, 0.22] | 0.2 | True (W = 0.883, p = 0.053) | True (rho = 0.468, p = 0.079) | -0.29 ± 0.36 | [-0.99, 0.41] | 0.4 | True (W = 0.966, p = 0.798) | True (rho = 0.314, p = 0.254) |
| ECG Langevin & Elgendi; PPG Elgendi & Elgendi | easy | 0.00 ± 0.01 | [-0.01, 0.02] | 0.01 | True (W = 0.978, p = 0.952) | True (rho = -0.011, p = 0.970) | 0.05 ± 0.20 | [-0.34, 0.43] | 0.19 | True (W = 0.968, p = 0.831) | True (rho = 0.111, p = 0.694) | 0.08 ± 0.28 | [-0.47, 0.64] | 0.27 | True (W = 0.977, p = 0.948) | True (rho = -0.125, p = 0.657) |
| ECG Langevin & Elgendi; PPG Elgendi & Elgendi | hard | 0.00 ± 0.01 | [-0.02, 0.02] | 0.01 | False (W = 0.792, p = 0.006) | True (rho = 0.099, p = 0.748) | 0.03 ± 0.26 | [-0.48, 0.53] | 0.26 | True (W = 0.933, p = 0.372) | True (rho = 0.033, p = 0.915) | 0.01 ± 0.36 | [-0.69, 0.71] | 0.35 | True (W = 0.957, p = 0.714) | True (rho = -0.269, p = 0.374) |
| ECG Langevin & Elgendi; PPG Langevin & Elgendi | easy | 0.00 ± 0.01 | [-0.01, 0.02] | 0.01 | True (W = 0.958, p = 0.655) | True (rho = 0.029, p = 0.919) | 0.10 ± 0.17 | [-0.22, 0.43] | 0.17 | True (W = 0.946, p = 0.462) | True (rho = -0.014, p = 0.960) | 0.17 ± 0.25 | [-0.32, 0.66] | 0.24 | True (W = 0.950, p = 0.527) | True (rho = -0.186, p = 0.508) |
| ECG Langevin & Elgendi; PPG Langevin & Elgendi | hard | -0.00 ± 0.01 | [-0.02, 0.01] | 0.01 | False (W = 0.695, p = 0.000) | True (rho = -0.170, p = 0.578) | 0.09 ± 0.25 | [-0.41, 0.59] | 0.26 | True (W = 0.906, p = 0.162) | True (rho = -0.181, p = 0.553) | 0.14 ± 0.36 | [-0.56, 0.84] | 0.36 | True (W = 0.917, p = 0.231) | True (rho = -0.335, p = 0.263) |
| ECG Langevin & Nabian; PPG Elgendi & Elgendi | easy | 0.00 ± 0.00 | [-0.01, 0.01] | 0 | False (W = 0.820, p = 0.007) | True (rho = 0.161, p = 0.567) | -0.17 ± 0.16 | [-0.48, 0.14] | 0.16 | True (W = 0.933, p = 0.298) | True (rho = 0.121, p = 0.666) | -0.31 ± 0.25 | [-0.79, 0.17] | 0.26 | True (W = 0.921, p = 0.197) | True (rho = 0.221, p = 0.428) |
| ECG Langevin & Nabian; PPG Elgendi & Elgendi | hard | 0.00 ± 0.01 | [-0.01, 0.01] | 0.01 | True (W = 0.908, p = 0.124) | True (rho = -0.046, p = 0.869) | -0.14 ± 0.20 | [-0.54, 0.25] | 0.22 | True (W = 0.940, p = 0.387) | True (rho = 0.193, p = 0.491) | -0.31 ± 0.33 | [-0.97, 0.34] | 0.36 | True (W = 0.942, p = 0.413) | True (rho = -0.032, p = 0.909) |
| ECG Langevin & Nabian; PPG Langevin & Elgendi | easy | -0.00 ± 0.00 | [-0.01, 0.01] | 0 | False (W = 0.833, p = 0.010) | True (rho = 0.268, p = 0.334) | -0.11 ± 0.13 | [-0.37, 0.15] | 0.14 | True (W = 0.965, p = 0.778) | True (rho = -0.100, p = 0.723) | -0.21 ± 0.20 | [-0.59, 0.18] | 0.21 | True (W = 0.918, p = 0.177) | True (rho = 0.271, p = 0.328) |
| ECG Langevin & Nabian; PPG Langevin & Elgendi | hard | -0.00 ± 0.00 | [-0.01, 0.01] | 0 | False (W = 0.693, p = 0.000) | True (rho = -0.500, p = 0.058) | -0.09 ± 0.18 | [-0.45, 0.28] | 0.2 | True (W = 0.953, p = 0.566) | True (rho = 0.389, p = 0.152) | -0.21 ± 0.33 | [-0.86, 0.45] | 0.37 | True (W = 0.933, p = 0.304) | True (rho = 0.186, p = 0.508) |
| ECG Langevin & Neurokit; PPG Elgendi & Elgendi | easy | 0.00 ± 0.00 | [-0.01, 0.01] | 0 | True (W = 0.903, p = 0.108) | True (rho = 0.057, p = 0.840) | -0.19 ± 0.16 | [-0.51, 0.13] | 0.17 | True (W = 0.919, p = 0.189) | True (rho = 0.057, p = 0.840) | -0.34 ± 0.24 | [-0.81, 0.13] | 0.25 | True (W = 0.948, p = 0.494) | True (rho = 0.118, p = 0.676) |
| ECG Langevin & Neurokit; PPG Elgendi & Elgendi | hard | -0.00 ± 0.01 | [-0.01, 0.01] | 0.01 | True (W = 0.900, p = 0.095) | True (rho = -0.225, p = 0.420) | -0.15 ± 0.19 | [-0.53, 0.23] | 0.2 | True (W = 0.935, p = 0.318) | True (rho = -0.146, p = 0.603) | -0.30 ± 0.32 | [-0.92, 0.32] | 0.34 | True (W = 0.943, p = 0.424) | True (rho = -0.036, p = 0.899) |
| ECG Langevin & Neurokit; PPG Langevin & Elgendi | easy | -0.00 ± 0.00 | [-0.01, 0.01] | 0 | True (W = 0.890, p = 0.068) | True (rho = 0.304, p = 0.271) | -0.13 ± 0.13 | [-0.39, 0.14] | 0.14 | True (W = 0.952, p = 0.548) | True (rho = -0.161, p = 0.567) | -0.24 ± 0.20 | [-0.62, 0.15] | 0.21 | True (W = 0.958, p = 0.661) | True (rho = 0.136, p = 0.630) |
| ECG Langevin & Neurokit; PPG Langevin & Elgendi | hard | -0.00 ± 0.00 | [-0.01, 0.01] | 0.01 | False (W = 0.779, p = 0.002) | True (rho = -0.396, p = 0.143) | -0.09 ± 0.17 | [-0.43, 0.25] | 0.19 | True (W = 0.907, p = 0.122) | True (rho = 0.261, p = 0.348) | -0.19 ± 0.32 | [-0.81, 0.43] | 0.34 | True (W = 0.940, p = 0.383) | True (rho = 0.021, p = 0.940) |
| ECG Neurokit & Elgendi; PPG Elgendi & Elgendi | easy | 0.03 ± 0.07 | [-0.10, 0.17] | 0.07 | False (W = 0.546, p = 0.000) | False (rho = 0.675, p = 0.006) | -0.01 ± 0.28 | [-0.56, 0.54] | 0.28 | True (W = 0.951, p = 0.543) | True (rho = -0.318, p = 0.248) | -0.14 ± 0.36 | [-0.85, 0.56] | 0.36 | True (W = 0.912, p = 0.147) | True (rho = -0.321, p = 0.243) |
| ECG Neurokit & Elgendi; PPG Elgendi & Elgendi | hard | 0.01 ± 0.03 | [-0.04, 0.07] | 0.03 | False (W = 0.554, p = 0.000) | False (rho = 0.625, p = 0.013) | 0.01 ± 0.31 | [-0.60, 0.62] | 0.32 | True (W = 0.953, p = 0.565) | True (rho = -0.164, p = 0.558) | -0.12 ± 0.42 | [-0.95, 0.71] | 0.43 | True (W = 0.951, p = 0.543) | True (rho = -0.257, p = 0.355) |
| ECG Neurokit & Elgendi; PPG Langevin & Elgendi | easy | 0.03 ± 0.07 | [-0.11, 0.17] | 0.07 | False (W = 0.538, p = 0.000) | False (rho = 0.543, p = 0.037) | 0.07 ± 0.24 | [-0.41, 0.54] | 0.25 | True (W = 0.946, p = 0.460) | True (rho = -0.025, p = 0.930) | -0.04 ± 0.29 | [-0.60, 0.53] | 0.29 | True (W = 0.920, p = 0.193) | True (rho = -0.386, p = 0.156) |
| ECG Neurokit & Elgendi; PPG Langevin & Elgendi | hard | 0.01 ± 0.03 | [-0.05, 0.07] | 0.03 | False (W = 0.531, p = 0.000) | True (rho = 0.346, p = 0.206) | 0.06 ± 0.29 | [-0.51, 0.64] | 0.3 | True (W = 0.982, p = 0.979) | True (rho = -0.229, p = 0.413) | -0.02 ± 0.42 | [-0.85, 0.81] | 0.44 | True (W = 0.952, p = 0.559) | True (rho = -0.246, p = 0.376) |
| ECG Neurokit & Nabian; PPG Elgendi & Elgendi | easy | 0.00 ± 0.00 | [-0.01, 0.01] | 0 | True (W = 0.928, p = 0.251) | True (rho = 0.157, p = 0.576) | -0.24 ± 0.26 | [-0.75, 0.27] | 0.27 | True (W = 0.912, p = 0.143) | True (rho = -0.296, p = 0.283) | -0.42 ± 0.38 | [-1.17, 0.32] | 0.4 | True (W = 0.943, p = 0.415) | True (rho = -0.386, p = 0.156) |
| ECG Neurokit & Nabian; PPG Elgendi & Elgendi | hard | -0.00 ± 0.01 | [-0.01, 0.01] | 0.01 | True (W = 0.915, p = 0.160) | True (rho = 0.279, p = 0.315) | -0.18 ± 0.23 | [-0.62, 0.27] | 0.24 | True (W = 0.904, p = 0.110) | True (rho = 0.054, p = 0.850) | -0.38 ± 0.39 | [-1.14, 0.39] | 0.42 | True (W = 0.961, p = 0.712) | True (rho = -0.179, p = 0.524) |
| ECG Neurokit & Nabian; PPG Langevin & Elgendi | easy | -0.00 ± 0.00 | [-0.01, 0.01] | 0 | False (W = 0.846, p = 0.015) | True (rho = 0.343, p = 0.211) | -0.15 ± 0.19 | [-0.51, 0.22] | 0.2 | True (W = 0.926, p = 0.240) | True (rho = -0.343, p = 0.211) | -0.29 ± 0.30 | [-0.87, 0.29] | 0.32 | True (W = 0.975, p = 0.922) | True (rho = -0.239, p = 0.390) |
| ECG Neurokit & Nabian; PPG Langevin & Elgendi | hard | -0.00 ± 0.01 | [-0.01, 0.01] | 0.01 | False (W = 0.692, p = 0.000) | True (rho = -0.064, p = 0.820) | -0.12 ± 0.20 | [-0.52, 0.27] | 0.22 | False (W = 0.881, p = 0.049) | True (rho = 0.275, p = 0.321) | -0.28 ± 0.38 | [-1.03, 0.47] | 0.42 | True (W = 0.939, p = 0.371) | True (rho = 0.261, p = 0.348) |
| ECG Neurokit & Neurokit; PPG Elgendi & Elgendi | easy | 0.00 ± 0.00 | [-0.01, 0.01] | 0 | True (W = 0.955, p = 0.605) | True (rho = -0.004, p = 0.990) | -0.28 ± 0.23 | [-0.72, 0.17] | 0.24 | True (W = 0.921, p = 0.200) | True (rho = -0.239, p = 0.390) | -0.50 ± 0.31 | [-1.11, 0.10] | 0.34 | True (W = 0.942, p = 0.405) | True (rho = 0.025, p = 0.930) |
| ECG Neurokit & Neurokit; PPG Elgendi & Elgendi | hard | -0.00 ± 0.01 | [-0.02, 0.01] | 0.01 | True (W = 0.932, p = 0.295) | True (rho = 0.461, p = 0.084) | -0.17 ± 0.26 | [-0.67, 0.34] | 0.28 | True (W = 0.959, p = 0.671) | True (rho = 0.107, p = 0.704) | -0.40 ± 0.38 | [-1.15, 0.34] | 0.42 | True (W = 0.972, p = 0.885) | True (rho = -0.096, p = 0.732) |
| ECG Neurokit & Neurokit; PPG Langevin & Elgendi | easy | -0.00 ± 0.00 | [-0.01, 0.01] | 0 | False (W = 0.829, p = 0.009) | True (rho = 0.404, p = 0.136) | -0.18 ± 0.15 | [-0.48, 0.11] | 0.16 | True (W = 0.935, p = 0.322) | True (rho = -0.286, p = 0.302) | -0.36 ± 0.22 | [-0.80, 0.07] | 0.25 | True (W = 0.958, p = 0.663) | True (rho = 0.089, p = 0.752) |
| ECG Neurokit & Neurokit; PPG Langevin & Elgendi | hard | -0.00 ± 0.01 | [-0.02, 0.01] | 0.01 | False (W = 0.770, p = 0.002) | True (rho = -0.061, p = 0.830) | -0.11 ± 0.23 | [-0.57, 0.34] | 0.25 | True (W = 0.954, p = 0.586) | True (rho = 0.214, p = 0.443) | -0.31 ± 0.38 | [-1.04, 0.43] | 0.42 | True (W = 0.972, p = 0.882) | True (rho = 0.239, p = 0.390) |

*Note*. The BAr results and corresponding values for all CVA parameters of interest across tested pipelines. The pipeline annotation consists of “ECG data cleaning method & peak detection method; PPG data cleaning method & peak detection method”. The pipeline configuration used for creating the Bland-Altman plots is marked with an asterisk.

Table 7
*BAr Results for CVA Parameters Across Processing Pipelines*

| Processing pipeline | Workload condition | HR (bpm) | | | | | | SD RR/PP interval (ms) | | | | | | RMSSD (ms) | | | | | |
| --- | --- | --- | --- | --- | --- | --- | --- | --- | --- | --- | --- | --- | --- | --- | --- | --- | --- | --- | --- |
|  |  | Mean ± SD | 95% CI | BAr | Within boundaries (%) | normality | homoscedasticity | Mean ± SD | 95% CI | BAr | Within boundaries (%) | normality | homoscedasticity | Mean ± SD | 95% CI | BAr | Within boundaries (%) | normality | homoscedasticity |
| ECG BioSPPY & Elgendi; PPG Elgendi & Elgendi | easy | 5.00 ± 14.40 | [-23.22, 33.21] | 0.39 | 86.67 | False (W = 0.380, p = 0.000) | False (rho = 0.761, p = 0.001) | -6.48 ± 60.25 | [-124.58, 111.62] | 1.23 | 60 | True (W = 0.959, p = 0.682) | True (rho = 0.050, p = 0.860) | -42.68 ± 75.71 | [-191.07, 105.71] | 1.46 | 53.33 | True (W = 0.972, p = 0.889) | False (rho = 0.539, p = 0.038) |
| ECG BioSPPY & Elgendi; PPG Elgendi & Elgendi | hard | 1.72 ± 4.32 | [-6.74, 10.18] | 0.12 | 93.33 | False (W = 0.559, p = 0.000) | True (rho = 0.496, p = 0.060) | -6.61 ± 58.04 | [-120.35, 107.14] | 1.33 | 66.67 | True (W = 0.989, p = 0.999) | True (rho = 0.264, p = 0.341) | -41.46 ± 75.61 | [-189.66, 106.75] | 1.67 | 60 | True (W = 0.944, p = 0.430) | True (rho = 0.471, p = 0.076) |
| ECG BioSPPY & Elgendi; PPG Langevin & Elgendi | easy | 4.91 ± 14.52 | [-23.55, 33.38] | 0.39 | 80 | False (W = 0.397, p = 0.000) | False (rho = 0.600, p = 0.018) | 11.86 ± 51.89 | [-89.85, 113.57] | 1.14 | 66.67 | True (W = 0.918, p = 0.178) | False (rho = 0.514, p = 0.050) | -11.60 ± 56.93 | [-123.19, 99.98] | 1.27 | 80 | True (W = 0.969, p = 0.840) | True (rho = 0.357, p = 0.191) |
| ECG BioSPPY & Elgendi; PPG Langevin & Elgendi | hard | 1.23 ± 4.42 | [-7.43, 9.89] | 0.12 | 93.33 | False (W = 0.526, p = 0.000) | True (rho = 0.193, p = 0.491) | 4.20 ± 53.14 | [-99.96, 108.36] | 1.3 | 73.33 | True (W = 0.976, p = 0.936) | True (rho = 0.214, p = 0.443) | -19.97 ± 67.60 | [-152.47, 112.53] | 1.67 | 60 | True (W = 0.961, p = 0.701) | True (rho = 0.468, p = 0.079) |
| ECG BioSPPY & Nabian; PPG Elgendi & Elgendi | easy | 0.17 ± 0.62 | [-1.04, 1.37] | 0.02 | 100 | True (W = 0.966, p = 0.790) | False (rho = 0.518, p = 0.048) | -36.57 ± 47.45 | [-129.57, 56.43] | 1.13 | 73.33 | True (W = 0.960, p = 0.698) | True (rho = 0.296, p = 0.283) | -67.77 ± 77.71 | [-220.08, 84.53] | 1.7 | 53.33 | True (W = 0.973, p = 0.895) | True (rho = 0.496, p = 0.060) |
| ECG BioSPPY & Nabian; PPG Elgendi & Elgendi | hard | -0.02 ± 1.00 | [-1.97, 1.93] | 0.03 | 100 | True (W = 0.925, p = 0.233) | True (rho = 0.186, p = 0.508) | -29.25 ± 37.28 | [-102.31, 43.82] | 0.99 | 73.33 | True (W = 0.882, p = 0.051) | True (rho = 0.211, p = 0.451) | -60.90 ± 63.64 | [-185.64, 63.84] | 1.58 | 60 | True (W = 0.923, p = 0.214) | True (rho = 0.350, p = 0.201) |
| ECG BioSPPY & Nabian; PPG Langevin & Elgendi | easy | -0.02 ± 0.63 | [-1.26, 1.21] | 0.02 | 100 | True (W = 0.904, p = 0.109) | False (rho = 0.546, p = 0.035) | -17.30 ± 33.98 | [-83.91, 49.30] | 0.9 | 93.33 | True (W = 0.971, p = 0.871) | True (rho = 0.132, p = 0.639) | -34.72 ± 59.16 | [-150.67, 81.23] | 1.54 | 66.67 | True (W = 0.950, p = 0.517) | True (rho = 0.411, p = 0.128) |
| ECG BioSPPY & Nabian; PPG Langevin & Elgendi | hard | -0.46 ± 0.83 | [-2.09, 1.16] | 0.02 | 100 | False (W = 0.750, p = 0.001) | True (rho = -0.150, p = 0.594) | -18.55 ± 31.14 | [-79.59, 42.48] | 0.89 | 86.67 | True (W = 0.889, p = 0.065) | True (rho = 0.239, p = 0.390) | -40.92 ± 59.10 | [-156.76, 74.92] | 1.68 | 60 | True (W = 0.907, p = 0.123) | False (rho = 0.532, p = 0.041) |
| ECG BioSPPY & Neurokit; PPG Elgendi & Elgendi | easy | 0.10 ± 0.53 | [-0.93, 1.14] | 0.01 | 100 | False (W = 0.858, p = 0.022) | True (rho = 0.096, p = 0.732) | -40.97 ± 37.77 | [-114.99, 33.05] | 0.92 | 73.33 | True (W = 0.942, p = 0.404) | True (rho = 0.396, p = 0.143) | -80.02 ± 61.24 | [-200.06, 40.01] | 1.43 | 46.67 | True (W = 0.965, p = 0.784) | False (rho = 0.768, p = 0.001) |
| ECG BioSPPY & Neurokit; PPG Elgendi & Elgendi | hard | -0.20 ± 1.14 | [-2.43, 2.04] | 0.03 | 100 | True (W = 0.930, p = 0.273) | True (rho = 0.414, p = 0.125) | -19.77 ± 56.23 | [-129.99, 90.44] | 1.4 | 66.67 | False (W = 0.846, p = 0.015) | True (rho = 0.411, p = 0.128) | -46.55 ± 88.35 | [-219.71, 126.61] | 2.02 | 53.33 | False (W = 0.866, p = 0.030) | False (rho = 0.718, p = 0.003) |
| ECG BioSPPY & Neurokit; PPG Langevin & Elgendi | easy | -0.15 ± 0.60 | [-1.32, 1.02] | 0.02 | 100 | False (W = 0.772, p = 0.002) | True (rho = 0.343, p = 0.211) | -24.40 ± 25.12 | [-73.64, 24.83] | 0.69 | 93.33 | True (W = 0.943, p = 0.421) | True (rho = 0.246, p = 0.376) | -49.69 ± 41.54 | [-131.11, 31.73] | 1.18 | 73.33 | True (W = 0.940, p = 0.378) | False (rho = 0.618, p = 0.014) |
| ECG BioSPPY & Neurokit; PPG Langevin & Elgendi | hard | -0.62 ± 0.88 | [-2.35, 1.11] | 0.02 | 100 | False (W = 0.790, p = 0.003) | True (rho = 0.000, p = 1.000) | -9.16 ± 53.69 | [-114.38, 96.07] | 1.44 | 80 | False (W = 0.780, p = 0.002) | True (rho = 0.486, p = 0.066) | -26.60 ± 87.71 | [-198.52, 145.32] | 2.26 | 53.33 | False (W = 0.837, p = 0.011) | False (rho = 0.718, p = 0.003) |
| ECG Elgendi & Elgendi; PPG Elgendi & Elgendi | easy | 0.45 ± 1.01 | [-1.53, 2.44] | 0.03 | 100 | False (W = 0.802, p = 0.004) | True (rho = 0.032, p = 0.909) | -40.98 ± 38.03 | [-115.52, 33.57] | 0.89 | 73.33 | True (W = 0.937, p = 0.346) | True (rho = 0.150, p = 0.594) | -73.20 ± 66.07 | [-202.69, 56.30] | 1.4 | 46.67 | True (W = 0.986, p = 0.995) | False (rho = 0.611, p = 0.016) |
| ECG Elgendi & Elgendi; PPG Elgendi & Elgendi | hard | 0.23 ± 1.02 | [-1.78, 2.23] | 0.03 | 100 | True (W = 0.920, p = 0.223) | True (rho = 0.160, p = 0.584) | -30.64 ± 38.43 | [-105.95, 44.67] | 1.04 | 71.43 | True (W = 0.927, p = 0.279) | True (rho = 0.433, p = 0.122) | -62.62 ± 65.42 | [-190.85, 65.61] | 1.61 | 57.14 | True (W = 0.960, p = 0.722) | False (rho = 0.569, p = 0.034) |
| ECG Elgendi & Elgendi; PPG Langevin & Elgendi | easy | 0.25 ± 1.09 | [-1.88, 2.38] | 0.03 | 100 | False (W = 0.768, p = 0.001) | True (rho = -0.079, p = 0.781) | -21.91 ± 22.97 | [-66.94, 23.12] | 0.59 | 93.33 | True (W = 0.917, p = 0.174) | True (rho = -0.043, p = 0.879) | -40.57 ± 44.75 | [-128.28, 47.13] | 1.11 | 86.67 | True (W = 0.970, p = 0.862) | False (rho = 0.611, p = 0.016) |
| ECG Elgendi & Elgendi; PPG Langevin & Elgendi | hard | -0.22 ± 0.79 | [-1.76, 1.33] | 0.02 | 100 | False (W = 0.656, p = 0.000) | True (rho = 0.051, p = 0.864) | -20.04 ± 31.86 | [-82.50, 42.41] | 0.93 | 85.71 | True (W = 0.932, p = 0.326) | True (rho = 0.459, p = 0.098) | -42.33 ± 60.73 | [-161.37, 76.71] | 1.71 | 57.14 | True (W = 0.955, p = 0.640) | True (rho = 0.503, p = 0.067) |
| ECG Elgendi & Nabian; PPG Elgendi & Elgendi | easy | 0.07 ± 0.59 | [-1.08, 1.23] | 0.02 | 100 | True (W = 0.958, p = 0.663) | True (rho = 0.182, p = 0.516) | -45.11 ± 39.85 | [-123.22, 33.00] | 1 | 73.33 | True (W = 0.945, p = 0.454) | True (rho = 0.289, p = 0.296) | -83.89 ± 62.94 | [-207.26, 39.48] | 1.5 | 40 | True (W = 0.969, p = 0.848) | False (rho = 0.832, p = 0.000) |
| ECG Elgendi & Nabian; PPG Elgendi & Elgendi | hard | 0.12 ± 0.97 | [-1.78, 2.03] | 0.03 | 100 | True (W = 0.903, p = 0.106) | True (rho = 0.257, p = 0.355) | -34.04 ± 34.51 | [-101.69, 33.60] | 0.95 | 73.33 | True (W = 0.914, p = 0.155) | True (rho = 0.464, p = 0.081) | -72.11 ± 56.78 | [-183.40, 39.19] | 1.52 | 60 | True (W = 0.968, p = 0.823) | False (rho = 0.743, p = 0.002) |
| ECG Elgendi & Nabian; PPG Langevin & Elgendi * | easy | -0.10 ± 0.62 | [-1.31, 1.11] | 0.02 | 100 | False (W = 0.835, p = 0.011) | True (rho = 0.396, p = 0.143) | -26.36 ± 24.65 | [-74.68, 21.95] | 0.68 | 93.33 | True (W = 0.953, p = 0.581) | True (rho = 0.214, p = 0.443) | -51.88 ± 41.49 | [-133.19, 29.44] | 1.19 | 73.33 | True (W = 0.975, p = 0.925) | False (rho = 0.664, p = 0.007) |
| ECG Elgendi & Nabian; PPG Langevin & Elgendi * | hard | -0.31 ± 0.73 | [-1.73, 1.12] | 0.02 | 100 | False (W = 0.634, p = 0.000) | True (rho = -0.193, p = 0.491) | -23.41 ± 28.46 | [-79.18, 32.37] | 0.84 | 86.67 | True (W = 0.935, p = 0.320) | True (rho = 0.496, p = 0.060) | -51.78 ± 52.54 | [-154.76, 51.19] | 1.62 | 60 | True (W = 0.955, p = 0.611) | False (rho = 0.807, p = 0.000) |
| ECG Elgendi & Neurokit; PPG Elgendi & Elgendi | easy | 0.14 ± 0.60 | [-1.03, 1.31] | 0.02 | 100 | True (W = 0.932, p = 0.294) | True (rho = 0.393, p = 0.147) | -41.31 ± 38.94 | [-117.64, 35.02] | 0.95 | 73.33 | True (W = 0.957, p = 0.639) | True (rho = 0.496, p = 0.060) | -74.98 ± 62.38 | [-197.25, 47.29] | 1.41 | 46.67 | True (W = 0.959, p = 0.671) | False (rho = 0.768, p = 0.001) |
| ECG Elgendi & Neurokit; PPG Elgendi & Elgendi | hard | 0.09 ± 0.93 | [-1.74, 1.92] | 0.03 | 100 | True (W = 0.918, p = 0.181) | True (rho = 0.332, p = 0.226) | -31.82 ± 35.73 | [-101.84, 38.21] | 0.96 | 73.33 | True (W = 0.912, p = 0.146) | False (rho = 0.539, p = 0.038) | -64.85 ± 60.47 | [-183.37, 53.66] | 1.54 | 60 | True (W = 0.971, p = 0.869) | False (rho = 0.711, p = 0.003) |
| ECG Elgendi & Neurokit; PPG Langevin & Elgendi | easy | -0.07 ± 0.64 | [-1.32, 1.18] | 0.02 | 100 | False (W = 0.795, p = 0.003) | True (rho = 0.439, p = 0.101) | -23.16 ± 25.84 | [-73.81, 27.49] | 0.7 | 93.33 | True (W = 0.961, p = 0.706) | True (rho = 0.461, p = 0.084) | -44.49 ± 43.93 | [-130.58, 41.60] | 1.2 | 80 | True (W = 0.958, p = 0.649) | False (rho = 0.600, p = 0.018) |
| ECG Elgendi & Neurokit; PPG Langevin & Elgendi | hard | -0.31 ± 0.74 | [-1.77, 1.14] | 0.02 | 100 | False (W = 0.684, p = 0.000) | True (rho = -0.225, p = 0.420) | -21.16 ± 29.72 | [-79.41, 37.09] | 0.86 | 86.67 | True (W = 0.944, p = 0.439) | False (rho = 0.611, p = 0.016) | -43.95 ± 56.46 | [-154.61, 66.72] | 1.64 | 66.67 | True (W = 0.960, p = 0.686) | False (rho = 0.775, p = 0.001) |
| ECG Langevin & Elgendi; PPG Elgendi & Elgendi | easy | 0.22 ± 1.30 | [-2.33, 2.77] | 0.04 | 100 | True (W = 0.969, p = 0.842) | True (rho = 0.157, p = 0.576) | 11.42 ± 48.20 | [-83.06, 105.90] | 0.91 | 80 | True (W = 0.975, p = 0.926) | True (rho = 0.379, p = 0.164) | 23.08 ± 79.51 | [-132.75, 178.91] | 1.18 | 60 | True (W = 0.969, p = 0.843) | True (rho = 0.157, p = 0.576) |
| ECG Langevin & Elgendi; PPG Elgendi & Elgendi | hard | 0.27 ± 1.42 | [-2.51, 3.05] | 0.04 | 100 | False (W = 0.824, p = 0.013) | True (rho = 0.209, p = 0.494) | 7.97 ± 61.72 | [-113.01, 128.95] | 1.32 | 92.31 | False (W = 0.829, p = 0.015) | True (rho = 0.434, p = 0.138) | 5.32 ± 90.32 | [-171.71, 182.35] | 1.55 | 53.85 | True (W = 0.933, p = 0.368) | True (rho = 0.357, p = 0.231) |
| ECG Langevin & Elgendi; PPG Langevin & Elgendi | easy | 0.01 ± 1.31 | [-2.55, 2.58] | 0.04 | 100 | True (W = 0.936, p = 0.333) | True (rho = 0.068, p = 0.810) | 24.08 ± 38.25 | [-50.90, 99.06] | 0.79 | 86.67 | True (W = 0.974, p = 0.908) | True (rho = 0.511, p = 0.052) | 46.46 ± 67.36 | [-85.56, 178.49] | 1.13 | 60 | True (W = 0.980, p = 0.967) | True (rho = 0.346, p = 0.206) |
| ECG Langevin & Elgendi; PPG Langevin & Elgendi | hard | -0.27 ± 1.20 | [-2.62, 2.07] | 0.03 | 100 | False (W = 0.730, p = 0.001) | True (rho = -0.088, p = 0.775) | 19.58 ± 58.98 | [-96.02, 135.19] | 1.34 | 84.62 | False (W = 0.805, p = 0.008) | True (rho = 0.115, p = 0.707) | 29.65 ± 85.86 | [-138.63, 197.93] | 1.61 | 69.23 | True (W = 0.888, p = 0.091) | True (rho = 0.236, p = 0.437) |
| ECG Langevin & Nabian; PPG Elgendi & Elgendi | easy | 0.27 ± 0.60 | [-0.92, 1.45] | 0.02 | 100 | False (W = 0.819, p = 0.007) | True (rho = 0.346, p = 0.206) | -32.64 ± 33.39 | [-98.09, 32.81] | 0.77 | 80 | True (W = 0.984, p = 0.989) | True (rho = 0.414, p = 0.125) | -65.40 ± 55.80 | [-174.77, 43.97] | 1.19 | 53.33 | True (W = 0.967, p = 0.804) | False (rho = 0.636, p = 0.011) |
| ECG Langevin & Nabian; PPG Elgendi & Elgendi | hard | 0.07 ± 0.98 | [-1.85, 1.99] | 0.03 | 100 | True (W = 0.922, p = 0.205) | True (rho = 0.039, p = 0.889) | -24.85 ± 37.83 | [-98.99, 49.30] | 0.98 | 80 | True (W = 0.933, p = 0.308) | False (rho = 0.579, p = 0.024) | -53.77 ± 64.27 | [-179.73, 72.19] | 1.54 | 60 | True (W = 0.943, p = 0.426) | True (rho = 0.504, p = 0.056) |
| ECG Langevin & Nabian; PPG Langevin & Elgendi | easy | -0.03 ± 0.60 | [-1.20, 1.14] | 0.02 | 100 | False (W = 0.788, p = 0.003) | True (rho = 0.489, p = 0.064) | -17.35 ± 24.55 | [-65.48, 30.77] | 0.64 | 93.33 | True (W = 0.986, p = 0.996) | True (rho = 0.296, p = 0.283) | -36.20 ± 38.96 | [-112.56, 40.16] | 1 | 80 | True (W = 0.966, p = 0.797) | False (rho = 0.557, p = 0.031) |
| ECG Langevin & Nabian; PPG Langevin & Elgendi | hard | -0.39 ± 0.69 | [-1.75, 0.96] | 0.02 | 100 | False (W = 0.717, p = 0.000) | True (rho = -0.300, p = 0.277) | -13.86 ± 32.00 | [-76.58, 48.87] | 0.89 | 93.33 | True (W = 0.946, p = 0.459) | True (rho = 0.446, p = 0.095) | -33.18 ± 58.80 | [-148.44, 82.07] | 1.6 | 66.67 | True (W = 0.926, p = 0.238) | False (rho = 0.604, p = 0.017) |
| ECG Langevin & Neurokit; PPG Elgendi & Elgendi | easy | 0.23 ± 0.70 | [-1.14, 1.60] | 0.02 | 100 | True (W = 0.883, p = 0.053) | True (rho = 0.143, p = 0.612) | -34.64 ± 33.39 | [-100.08, 30.80] | 0.78 | 80 | True (W = 0.970, p = 0.852) | True (rho = 0.429, p = 0.111) | -67.23 ± 55.97 | [-176.94, 42.48] | 1.21 | 53.33 | True (W = 0.944, p = 0.434) | False (rho = 0.593, p = 0.020) |
| ECG Langevin & Neurokit; PPG Elgendi & Elgendi | hard | -0.07 ± 1.00 | [-2.03, 1.89] | 0.03 | 100 | True (W = 0.917, p = 0.173) | True (rho = -0.032, p = 0.909) | -26.12 ± 35.86 | [-96.41, 44.17] | 0.93 | 80 | True (W = 0.912, p = 0.145) | True (rho = 0.218, p = 0.435) | -54.69 ± 61.20 | [-174.64, 65.27] | 1.45 | 60 | True (W = 0.934, p = 0.316) | False (rho = 0.529, p = 0.043) |
| ECG Langevin & Neurokit; PPG Langevin & Elgendi | easy | -0.08 ± 0.70 | [-1.45, 1.28] | 0.02 | 100 | False (W = 0.847, p = 0.016) | True (rho = 0.414, p = 0.125) | -19.44 ± 24.10 | [-66.67, 27.79] | 0.64 | 93.33 | True (W = 0.980, p = 0.968) | True (rho = 0.186, p = 0.508) | -38.57 ± 39.50 | [-116.00, 38.85] | 1.03 | 80 | True (W = 0.955, p = 0.606) | True (rho = 0.371, p = 0.173) |
| ECG Langevin & Neurokit; PPG Langevin & Elgendi | hard | -0.52 ± 0.76 | [-2.02, 0.97] | 0.02 | 100 | False (W = 0.808, p = 0.005) | True (rho = -0.296, p = 0.283) | -15.15 ± 29.64 | [-73.24, 42.93] | 0.83 | 93.33 | True (W = 0.908, p = 0.128) | True (rho = 0.411, p = 0.128) | -33.58 ± 54.46 | [-140.31, 73.16] | 1.48 | 66.67 | True (W = 0.928, p = 0.253) | False (rho = 0.518, p = 0.048) |
| ECG Neurokit & Elgendi; PPG Elgendi & Elgendi | easy | 6.82 ± 15.36 | [-23.28, 36.92] | 0.41 | 73.33 | False (W = 0.505, p = 0.000) | False (rho = 0.700, p = 0.004) | 3.81 ± 61.32 | [-116.38, 123.99] | 1.18 | 80 | True (W = 0.954, p = 0.591) | True (rho = -0.014, p = 0.960) | -34.62 ± 75.57 | [-182.73, 113.50] | 1.39 | 46.67 | True (W = 0.944, p = 0.434) | True (rho = 0.304, p = 0.271) |
| ECG Neurokit & Elgendi; PPG Elgendi & Elgendi | hard | 2.69 ± 5.37 | [-7.84, 13.22] | 0.14 | 86.67 | False (W = 0.544, p = 0.000) | False (rho = 0.682, p = 0.005) | 9.86 ± 63.52 | [-114.63, 134.35] | 1.32 | 60 | True (W = 0.945, p = 0.451) | True (rho = 0.161, p = 0.567) | -15.02 ± 93.00 | [-197.31, 167.27] | 1.78 | 53.33 | True (W = 0.916, p = 0.167) | True (rho = 0.418, p = 0.121) |
| ECG Neurokit & Elgendi; PPG Langevin & Elgendi | easy | 6.48 ± 15.45 | [-23.79, 36.76] | 0.41 | 80 | False (W = 0.500, p = 0.000) | False (rho = 0.668, p = 0.007) | 20.57 ± 52.38 | [-82.11, 123.24] | 1.1 | 86.67 | True (W = 0.939, p = 0.374) | True (rho = 0.368, p = 0.177) | -3.61 ± 56.11 | [-113.59, 106.36] | 1.19 | 80 | True (W = 0.932, p = 0.292) | True (rho = 0.175, p = 0.533) |
| ECG Neurokit & Elgendi; PPG Langevin & Elgendi | hard | 2.19 ± 5.54 | [-8.67, 13.05] | 0.15 | 86.67 | False (W = 0.522, p = 0.000) | True (rho = 0.393, p = 0.147) | 19.07 ± 60.16 | [-98.84, 136.99] | 1.34 | 80 | True (W = 0.901, p = 0.098) | True (rho = 0.204, p = 0.467) | 4.19 ± 89.00 | [-170.25, 178.63] | 1.9 | 46.67 | True (W = 0.909, p = 0.129) | False (rho = 0.604, p = 0.017) |
| ECG Neurokit & Nabian; PPG Elgendi & Elgendi | easy | 0.12 ± 0.64 | [-1.14, 1.39] | 0.02 | 100 | True (W = 0.939, p = 0.373) | True (rho = 0.407, p = 0.132) | -39.45 ± 44.90 | [-127.45, 48.55] | 1.09 | 73.33 | True (W = 0.950, p = 0.522) | True (rho = 0.243, p = 0.383) | -72.71 ± 72.12 | [-214.07, 68.64] | 1.61 | 53.33 | True (W = 0.956, p = 0.617) | True (rho = 0.450, p = 0.092) |
| ECG Neurokit & Nabian; PPG Elgendi & Elgendi | hard | -0.06 ± 1.10 | [-2.22, 2.10] | 0.03 | 100 | True (W = 0.900, p = 0.097) | True (rho = 0.289, p = 0.296) | -27.35 ± 40.50 | [-106.72, 52.03] | 1.06 | 73.33 | True (W = 0.935, p = 0.326) | False (rho = 0.604, p = 0.017) | -56.23 ± 72.33 | [-197.99, 85.54] | 1.74 | 60 | True (W = 0.967, p = 0.813) | False (rho = 0.564, p = 0.028) |
| ECG Neurokit & Nabian; PPG Langevin & Elgendi | easy | -0.08 ± 0.66 | [-1.38, 1.21] | 0.02 | 100 | False (W = 0.803, p = 0.004) | True (rho = 0.432, p = 0.108) | -20.84 ± 30.34 | [-80.30, 38.61] | 0.81 | 86.67 | True (W = 0.966, p = 0.791) | True (rho = 0.189, p = 0.499) | -40.91 ± 51.05 | [-140.96, 59.15] | 1.36 | 73.33 | True (W = 0.958, p = 0.664) | True (rho = 0.218, p = 0.435) |
| ECG Neurokit & Nabian; PPG Langevin & Elgendi | hard | -0.48 ± 0.95 | [-2.34, 1.38] | 0.03 | 100 | False (W = 0.658, p = 0.000) | True (rho = 0.057, p = 0.840) | -16.90 ± 33.83 | [-83.21, 49.40] | 0.95 | 86.67 | True (W = 0.941, p = 0.398) | True (rho = 0.496, p = 0.060) | -36.53 ± 65.11 | [-164.14, 91.08] | 1.78 | 60 | True (W = 0.950, p = 0.517) | False (rho = 0.639, p = 0.010) |
| ECG Neurokit & Neurokit; PPG Elgendi & Elgendi | easy | 0.06 ± 0.60 | [-1.11, 1.23] | 0.02 | 100 | True (W = 0.938, p = 0.361) | True (rho = 0.182, p = 0.516) | -45.96 ± 39.09 | [-122.58, 30.66] | 0.98 | 73.33 | True (W = 0.926, p = 0.236) | True (rho = 0.221, p = 0.428) | -85.37 ± 61.10 | [-205.12, 34.39] | 1.47 | 46.67 | True (W = 0.950, p = 0.522) | False (rho = 0.775, p = 0.001) |
| ECG Neurokit & Neurokit; PPG Elgendi & Elgendi | hard | -0.25 ± 1.26 | [-2.72, 2.21] | 0.03 | 100 | True (W = 0.920, p = 0.196) | True (rho = 0.511, p = 0.052) | -23.27 ± 48.16 | [-117.66, 71.12] | 1.23 | 66.67 | True (W = 0.980, p = 0.968) | True (rho = 0.468, p = 0.079) | -60.31 ± 69.34 | [-196.22, 75.61] | 1.73 | 60 | True (W = 0.981, p = 0.974) | False (rho = 0.554, p = 0.032) |
| ECG Neurokit & Neurokit; PPG Langevin & Elgendi | easy | -0.14 ± 0.60 | [-1.31, 1.04] | 0.02 | 100 | False (W = 0.804, p = 0.004) | False (rho = 0.529, p = 0.043) | -27.40 ± 23.67 | [-73.80, 19.00] | 0.66 | 93.33 | True (W = 0.923, p = 0.214) | True (rho = 0.054, p = 0.850) | -53.57 ± 38.84 | [-129.70, 22.57] | 1.13 | 73.33 | True (W = 0.938, p = 0.361) | False (rho = 0.579, p = 0.024) |
| ECG Neurokit & Neurokit; PPG Langevin & Elgendi | hard | -0.68 ± 1.05 | [-2.74, 1.38] | 0.03 | 100 | False (W = 0.718, p = 0.000) | True (rho = 0.071, p = 0.800) | -13.39 ± 42.09 | [-95.88, 69.10] | 1.16 | 80 | True (W = 0.971, p = 0.878) | False (rho = 0.539, p = 0.038) | -41.33 ± 63.36 | [-165.52, 82.85] | 1.81 | 60 | True (W = 0.978, p = 0.951) | False (rho = 0.646, p = 0.009) |

*Note*. The BAr results and corresponding values for all CVA parameters of interest across tested pipelines. The pipeline annotation consists of “ECG data cleaning method & peak detection method; PPG data cleaning method & peak detection method”. The pipeline configuration used for creating the Bland-Altman plots is marked with an asterisk.

Table 8
*BAr Results for Log-Transformed CVA Parameters Across Processing Pipelines with Original ECG Sampling Rate*

| Processing pipeline | Workload condition | Log(HR) | | | | | Log(SD RR/PP interval) | | | | | Log(RMSSD) | | | | |
| --- | --- | --- | --- | --- | --- | --- | --- | --- | --- | --- | --- | --- | --- | --- | --- | --- |
|  |  | Mean ± SD | 95% CI | BAr | normality | homoscedasticity | Mean ± SD | 95% CI | BAr | normality | homoscedasticity | Mean ± SD | 95% CI | BAr | normality | homoscedasticity |
| ECG BioSPPY & Elgendi; PPG Elgendi & Elgendi | easy | 0.05 ± 0.09 | [-0.12, 0.22] | 0.09 | False (W = 0.660, p = 0.000) | False (rho = 0.650, p = 0.009) | -0.07 ± 0.33 | [-0.71, 0.57] | 0.33 | True (W = 0.976, p = 0.937) | True (rho = -0.454, p = 0.089) | -0.20 ± 0.43 | [-1.04, 0.65] | 0.43 | True (W = 0.914, p = 0.156) | True (rho = -0.321, p = 0.243) |
| ECG BioSPPY & Elgendi; PPG Elgendi & Elgendi | hard | 0.03 ± 0.08 | [-0.12, 0.18] | 0.08 | False (W = 0.475, p = 0.000) | False (rho = 0.514, p = 0.050) | -0.14 ± 0.31 | [-0.75, 0.46] | 0.33 | True (W = 0.945, p = 0.448) | True (rho = -0.279, p = 0.315) | -0.25 ± 0.45 | [-1.12, 0.63] | 0.47 | True (W = 0.976, p = 0.933) | True (rho = -0.286, p = 0.302) |
| ECG BioSPPY & Elgendi; PPG Langevin & Elgendi | easy | 0.04 ± 0.08 | [-0.11, 0.19] | 0.08 | False (W = 0.689, p = 0.000) | False (rho = 0.650, p = 0.009) | 0.03 ± 0.31 | [-0.57, 0.63] | 0.31 | True (W = 0.969, p = 0.848) | True (rho = 0.121, p = 0.666) | -0.07 ± 0.40 | [-0.84, 0.71] | 0.41 | True (W = 0.943, p = 0.417) | True (rho = -0.446, p = 0.095) |
| ECG BioSPPY & Elgendi; PPG Langevin & Elgendi | hard | 0.03 ± 0.08 | [-0.12, 0.18] | 0.08 | False (W = 0.461, p = 0.000) | True (rho = 0.479, p = 0.071) | -0.09 ± 0.31 | [-0.70, 0.53] | 0.34 | True (W = 0.967, p = 0.803) | True (rho = -0.193, p = 0.491) | -0.15 ± 0.46 | [-1.06, 0.75] | 0.5 | True (W = 0.963, p = 0.743) | True (rho = -0.021, p = 0.940) |
| ECG BioSPPY & Nabian; PPG Elgendi & Elgendi | easy | 0.00 ± 0.02 | [-0.03, 0.04] | 0.02 | True (W = 0.913, p = 0.150) | True (rho = -0.111, p = 0.694) | -0.32 ± 0.29 | [-0.88, 0.25] | 0.31 | True (W = 0.897, p = 0.087) | False (rho = -0.654, p = 0.008) | -0.49 ± 0.40 | [-1.27, 0.30] | 0.43 | True (W = 0.936, p = 0.333) | True (rho = -0.357, p = 0.191) |
| ECG BioSPPY & Nabian; PPG Elgendi & Elgendi | hard | 0.00 ± 0.01 | [-0.02, 0.03] | 0.01 | True (W = 0.973, p = 0.894) | True (rho = 0.218, p = 0.435) | -0.24 ± 0.29 | [-0.80, 0.33] | 0.31 | True (W = 0.970, p = 0.862) | True (rho = -0.121, p = 0.666) | -0.45 ± 0.44 | [-1.32, 0.42] | 0.49 | True (W = 0.939, p = 0.365) | True (rho = 0.050, p = 0.860) |
| ECG BioSPPY & Nabian; PPG Langevin & Elgendi | easy | 0.00 ± 0.02 | [-0.03, 0.03] | 0.02 | True (W = 0.934, p = 0.318) | True (rho = 0.236, p = 0.398) | -0.23 ± 0.22 | [-0.66, 0.21] | 0.24 | True (W = 0.906, p = 0.119) | False (rho = -0.607, p = 0.016) | -0.37 ± 0.35 | [-1.06, 0.32] | 0.39 | True (W = 0.944, p = 0.436) | True (rho = -0.275, p = 0.321) |
| ECG BioSPPY & Nabian; PPG Langevin & Elgendi | hard | 0.00 ± 0.01 | [-0.02, 0.03] | 0.02 | True (W = 0.977, p = 0.943) | True (rho = 0.157, p = 0.576) | -0.16 ± 0.28 | [-0.72, 0.39] | 0.31 | True (W = 0.963, p = 0.751) | True (rho = 0.082, p = 0.771) | -0.32 ± 0.45 | [-1.20, 0.55] | 0.5 | True (W = 0.965, p = 0.771) | True (rho = 0.179, p = 0.524) |
| ECG BioSPPY & Neurokit; PPG Elgendi & Elgendi | easy | 0.00 ± 0.02 | [-0.03, 0.04] | 0.02 | True (W = 0.912, p = 0.148) | True (rho = 0.018, p = 0.950) | -0.29 ± 0.31 | [-0.91, 0.32] | 0.33 | True (W = 0.951, p = 0.545) | True (rho = -0.496, p = 0.060) | -0.47 ± 0.42 | [-1.29, 0.36] | 0.45 | True (W = 0.927, p = 0.250) | True (rho = -0.254, p = 0.362) |
| ECG BioSPPY & Neurokit; PPG Elgendi & Elgendi | hard | 0.01 ± 0.01 | [-0.02, 0.03] | 0.01 | True (W = 0.968, p = 0.823) | True (rho = 0.386, p = 0.156) | -0.23 ± 0.28 | [-0.79, 0.33] | 0.31 | True (W = 0.949, p = 0.506) | True (rho = -0.471, p = 0.076) | -0.41 ± 0.44 | [-1.28, 0.46] | 0.49 | True (W = 0.965, p = 0.776) | True (rho = -0.461, p = 0.084) |
| ECG BioSPPY & Neurokit; PPG Langevin & Elgendi | easy | -0.00 ± 0.02 | [-0.03, 0.03] | 0.02 | True (W = 0.911, p = 0.139) | True (rho = 0.236, p = 0.398) | -0.20 ± 0.25 | [-0.69, 0.29] | 0.27 | True (W = 0.961, p = 0.712) | True (rho = -0.425, p = 0.114) | -0.35 ± 0.37 | [-1.08, 0.38] | 0.41 | True (W = 0.953, p = 0.571) | True (rho = 0.032, p = 0.909) |
| ECG BioSPPY & Neurokit; PPG Langevin & Elgendi | hard | 0.00 ± 0.01 | [-0.02, 0.03] | 0.01 | True (W = 0.957, p = 0.641) | True (rho = 0.214, p = 0.443) | -0.17 ± 0.27 | [-0.70, 0.37] | 0.3 | True (W = 0.952, p = 0.557) | False (rho = -0.625, p = 0.013) | -0.31 ± 0.45 | [-1.18, 0.57] | 0.5 | True (W = 0.988, p = 0.998) | True (rho = -0.354, p = 0.196) |
| ECG Elgendi & Elgendi; PPG Elgendi & Elgendi | easy | 0.01 ± 0.02 | [-0.03, 0.06] | 0.02 | False (W = 0.824, p = 0.008) | True (rho = -0.211, p = 0.451) | -0.25 ± 0.34 | [-0.91, 0.41] | 0.36 | True (W = 0.983, p = 0.986) | True (rho = -0.336, p = 0.221) | -0.40 ± 0.45 | [-1.28, 0.48] | 0.47 | False (W = 0.872, p = 0.036) | True (rho = 0.136, p = 0.630) |
| ECG Elgendi & Elgendi; PPG Elgendi & Elgendi | hard | 0.01 ± 0.02 | [-0.02, 0.04] | 0.02 | True (W = 0.893, p = 0.090) | True (rho = 0.433, p = 0.122) | -0.25 ± 0.22 | [-0.68, 0.18] | 0.24 | True (W = 0.929, p = 0.299) | True (rho = 0.336, p = 0.240) | -0.49 ± 0.34 | [-1.16, 0.18] | 0.38 | True (W = 0.978, p = 0.961) | True (rho = -0.253, p = 0.383) |
| ECG Elgendi & Elgendi; PPG Langevin & Elgendi | easy | 0.01 ± 0.02 | [-0.04, 0.05] | 0.02 | False (W = 0.810, p = 0.005) | True (rho = -0.064, p = 0.820) | -0.16 ± 0.28 | [-0.70, 0.38] | 0.3 | True (W = 0.966, p = 0.800) | True (rho = -0.054, p = 0.850) | -0.29 ± 0.39 | [-1.06, 0.48] | 0.43 | False (W = 0.879, p = 0.045) | True (rho = 0.314, p = 0.254) |
| ECG Elgendi & Elgendi; PPG Langevin & Elgendi | hard | 0.01 ± 0.01 | [-0.02, 0.04] | 0.02 | True (W = 0.944, p = 0.477) | True (rho = 0.297, p = 0.303) | -0.19 ± 0.23 | [-0.65, 0.26] | 0.26 | True (W = 0.912, p = 0.169) | True (rho = 0.169, p = 0.563) | -0.38 ± 0.37 | [-1.11, 0.35] | 0.43 | True (W = 0.981, p = 0.979) | True (rho = -0.222, p = 0.446) |
| ECG Elgendi & Nabian; PPG Elgendi & Elgendi | easy | 0.00 ± 0.02 | [-0.03, 0.04] | 0.02 | True (W = 0.904, p = 0.110) | True (rho = 0.082, p = 0.771) | -0.33 ± 0.28 | [-0.88, 0.22] | 0.3 | True (W = 0.974, p = 0.913) | True (rho = -0.511, p = 0.052) | -0.51 ± 0.40 | [-1.29, 0.26] | 0.43 | True (W = 0.888, p = 0.063) | True (rho = 0.050, p = 0.860) |
| ECG Elgendi & Nabian; PPG Elgendi & Elgendi | hard | 0.01 ± 0.02 | [-0.02, 0.04] | 0.02 | True (W = 0.928, p = 0.259) | True (rho = 0.361, p = 0.187) | -0.27 ± 0.25 | [-0.75, 0.21] | 0.27 | True (W = 0.970, p = 0.860) | True (rho = 0.075, p = 0.791) | -0.54 ± 0.33 | [-1.19, 0.11] | 0.38 | True (W = 0.983, p = 0.984) | True (rho = 0.050, p = 0.860) |
| ECG Elgendi & Nabian; PPG Langevin & Elgendi * | easy | 0.00 ± 0.02 | [-0.03, 0.03] | 0.02 | True (W = 0.919, p = 0.189) | True (rho = -0.046, p = 0.869) | -0.24 ± 0.22 | [-0.68, 0.20] | 0.24 | True (W = 0.960, p = 0.686) | True (rho = -0.404, p = 0.136) | -0.41 ± 0.35 | [-1.10, 0.28] | 0.4 | True (W = 0.918, p = 0.178) | True (rho = 0.175, p = 0.533) |
| ECG Elgendi & Nabian; PPG Langevin & Elgendi * | hard | 0.01 ± 0.02 | [-0.02, 0.04] | 0.02 | True (W = 0.947, p = 0.473) | True (rho = 0.229, p = 0.413) | -0.21 ± 0.25 | [-0.69, 0.27] | 0.27 | True (W = 0.956, p = 0.626) | True (rho = 0.096, p = 0.732) | -0.43 ± 0.35 | [-1.12, 0.25] | 0.41 | True (W = 0.980, p = 0.970) | True (rho = 0.093, p = 0.742) |
| ECG Elgendi & Neurokit; PPG Elgendi & Elgendi | easy | 0.00 ± 0.02 | [-0.03, 0.04] | 0.02 | True (W = 0.901, p = 0.100) | True (rho = 0.021, p = 0.940) | -0.31 ± 0.28 | [-0.86, 0.24] | 0.3 | True (W = 0.973, p = 0.896) | False (rho = -0.529, p = 0.043) | -0.46 ± 0.40 | [-1.25, 0.34] | 0.44 | True (W = 0.929, p = 0.262) | True (rho = -0.136, p = 0.630) |
| ECG Elgendi & Neurokit; PPG Elgendi & Elgendi | hard | 0.01 ± 0.02 | [-0.02, 0.04] | 0.02 | True (W = 0.906, p = 0.116) | True (rho = 0.461, p = 0.084) | -0.26 ± 0.25 | [-0.75, 0.23] | 0.27 | True (W = 0.978, p = 0.951) | True (rho = 0.068, p = 0.810) | -0.44 ± 0.39 | [-1.19, 0.32] | 0.42 | True (W = 0.980, p = 0.972) | True (rho = -0.243, p = 0.383) |
| ECG Elgendi & Neurokit; PPG Langevin & Elgendi | easy | 0.00 ± 0.02 | [-0.03, 0.03] | 0.02 | True (W = 0.919, p = 0.184) | True (rho = 0.071, p = 0.800) | -0.22 ± 0.22 | [-0.65, 0.21] | 0.24 | True (W = 0.962, p = 0.735) | True (rho = -0.461, p = 0.084) | -0.34 ± 0.36 | [-1.04, 0.36] | 0.39 | True (W = 0.960, p = 0.699) | True (rho = 0.100, p = 0.723) |
| ECG Elgendi & Neurokit; PPG Langevin & Elgendi | hard | 0.01 ± 0.02 | [-0.02, 0.04] | 0.02 | True (W = 0.926, p = 0.239) | True (rho = 0.339, p = 0.216) | -0.19 ± 0.26 | [-0.70, 0.31] | 0.29 | True (W = 0.956, p = 0.619) | True (rho = 0.118, p = 0.676) | -0.33 ± 0.42 | [-1.15, 0.49] | 0.47 | True (W = 0.978, p = 0.958) | True (rho = -0.086, p = 0.761) |
| ECG Langevin & Elgendi; PPG Elgendi & Elgendi | easy | 0.01 ± 0.02 | [-0.03, 0.05] | 0.02 | False (W = 0.798, p = 0.003) | True (rho = 0.368, p = 0.177) | -0.06 ± 0.34 | [-0.72, 0.61] | 0.34 | True (W = 0.894, p = 0.076) | False (rho = -0.650, p = 0.009) | -0.10 ± 0.42 | [-0.93, 0.73] | 0.42 | True (W = 0.961, p = 0.718) | False (rho = -0.671, p = 0.006) |
| ECG Langevin & Elgendi; PPG Elgendi & Elgendi | hard | 0.01 ± 0.02 | [-0.03, 0.05] | 0.02 | True (W = 0.939, p = 0.410) | True (rho = 0.398, p = 0.159) | -0.14 ± 0.31 | [-0.74, 0.46] | 0.32 | True (W = 0.896, p = 0.098) | True (rho = -0.345, p = 0.227) | -0.19 ± 0.46 | [-1.09, 0.72] | 0.47 | True (W = 0.900, p = 0.113) | True (rho = -0.363, p = 0.203) |
| ECG Langevin & Elgendi; PPG Langevin & Elgendi | easy | 0.01 ± 0.02 | [-0.03, 0.05] | 0.02 | False (W = 0.830, p = 0.009) | True (rho = 0.407, p = 0.132) | 0.05 ± 0.22 | [-0.39, 0.48] | 0.22 | True (W = 0.961, p = 0.709) | True (rho = 0.064, p = 0.820) | 0.03 ± 0.33 | [-0.62, 0.69] | 0.33 | True (W = 0.955, p = 0.601) | True (rho = -0.218, p = 0.435) |
| ECG Langevin & Elgendi; PPG Langevin & Elgendi | hard | 0.01 ± 0.02 | [-0.03, 0.04] | 0.02 | True (W = 0.973, p = 0.912) | True (rho = 0.275, p = 0.342) | -0.06 ± 0.32 | [-0.69, 0.57] | 0.34 | True (W = 0.912, p = 0.170) | True (rho = -0.314, p = 0.274) | -0.07 ± 0.51 | [-1.06, 0.92] | 0.53 | True (W = 0.878, p = 0.055) | True (rho = -0.389, p = 0.169) |
| ECG Langevin & Nabian; PPG Elgendi & Elgendi | easy | 0.00 ± 0.02 | [-0.03, 0.04] | 0.02 | True (W = 0.922, p = 0.206) | True (rho = -0.157, p = 0.576) | -0.28 ± 0.32 | [-0.91, 0.35] | 0.34 | True (W = 0.973, p = 0.896) | False (rho = -0.771, p = 0.001) | -0.45 ± 0.45 | [-1.34, 0.44] | 0.49 | True (W = 0.949, p = 0.501) | True (rho = -0.382, p = 0.160) |
| ECG Langevin & Nabian; PPG Elgendi & Elgendi | hard | 0.01 ± 0.02 | [-0.02, 0.04] | 0.02 | True (W = 0.927, p = 0.248) | True (rho = 0.307, p = 0.265) | -0.25 ± 0.28 | [-0.79, 0.29] | 0.3 | True (W = 0.971, p = 0.879) | True (rho = -0.232, p = 0.405) | -0.45 ± 0.45 | [-1.34, 0.43] | 0.5 | True (W = 0.965, p = 0.774) | True (rho = -0.368, p = 0.177) |
| ECG Langevin & Nabian; PPG Langevin & Elgendi | easy | 0.00 ± 0.02 | [-0.03, 0.03] | 0.02 | True (W = 0.930, p = 0.277) | True (rho = -0.111, p = 0.694) | -0.17 ± 0.24 | [-0.64, 0.30] | 0.26 | True (W = 0.953, p = 0.567) | False (rho = -0.625, p = 0.013) | -0.30 ± 0.37 | [-1.03, 0.42] | 0.4 | True (W = 0.943, p = 0.424) | True (rho = -0.307, p = 0.265) |
| ECG Langevin & Nabian; PPG Langevin & Elgendi | hard | 0.01 ± 0.02 | [-0.02, 0.04] | 0.02 | True (W = 0.952, p = 0.558) | True (rho = 0.164, p = 0.558) | -0.15 ± 0.28 | [-0.70, 0.41] | 0.31 | True (W = 0.946, p = 0.469) | True (rho = -0.154, p = 0.585) | -0.28 ± 0.46 | [-1.19, 0.63] | 0.52 | True (W = 0.978, p = 0.950) | True (rho = -0.346, p = 0.206) |
| ECG Langevin & Neurokit; PPG Elgendi & Elgendi | easy | -0.01 ± 0.05 | [-0.11, 0.08] | 0.05 | False (W = 0.798, p = 0.005) | True (rho = -0.086, p = 0.771) | 0.02 ± 0.45 | [-0.86, 0.90] | 0.44 | True (W = 0.923, p = 0.241) | True (rho = 0.235, p = 0.418) | -0.07 ± 0.59 | [-1.23, 1.09] | 0.57 | True (W = 0.981, p = 0.982) | True (rho = -0.095, p = 0.748) |
| ECG Langevin & Neurokit; PPG Elgendi & Elgendi | hard | -0.01 ± 0.04 | [-0.09, 0.06] | 0.04 | False (W = 0.813, p = 0.005) | True (rho = 0.171, p = 0.541) | -0.04 ± 0.49 | [-1.00, 0.92] | 0.51 | True (W = 0.953, p = 0.568) | True (rho = 0.146, p = 0.603) | -0.15 ± 0.66 | [-1.44, 1.15] | 0.67 | True (W = 0.959, p = 0.683) | True (rho = -0.082, p = 0.771) |
| ECG Langevin & Neurokit; PPG Langevin & Elgendi | easy | -0.01 ± 0.03 | [-0.06, 0.04] | 0.03 | True (W = 0.967, p = 0.837) | True (rho = 0.130, p = 0.659) | 0.09 ± 0.39 | [-0.68, 0.86] | 0.39 | True (W = 0.929, p = 0.297) | True (rho = 0.451, p = 0.106) | 0.04 ± 0.53 | [-1.01, 1.09] | 0.53 | True (W = 0.980, p = 0.978) | True (rho = 0.182, p = 0.533) |
| ECG Langevin & Neurokit; PPG Langevin & Elgendi | hard | -0.02 ± 0.04 | [-0.09, 0.06] | 0.04 | False (W = 0.831, p = 0.009) | True (rho = 0.132, p = 0.639) | 0.04 ± 0.48 | [-0.90, 0.97] | 0.5 | True (W = 0.957, p = 0.638) | True (rho = 0.293, p = 0.289) | -0.00 ± 0.64 | [-1.25, 1.25] | 0.66 | True (W = 0.980, p = 0.971) | True (rho = 0.075, p = 0.791) |
| ECG Neurokit & Elgendi; PPG Elgendi & Elgendi | easy | 0.06 ± 0.10 | [-0.13, 0.24] | 0.1 | False (W = 0.615, p = 0.000) | False (rho = 0.793, p = 0.000) | -0.02 ± 0.31 | [-0.63, 0.60] | 0.31 | True (W = 0.954, p = 0.594) | False (rho = -0.611, p = 0.016) | -0.11 ± 0.40 | [-0.90, 0.69] | 0.4 | True (W = 0.974, p = 0.916) | True (rho = -0.475, p = 0.074) |
| ECG Neurokit & Elgendi; PPG Elgendi & Elgendi | hard | 0.05 ± 0.08 | [-0.11, 0.20] | 0.08 | False (W = 0.667, p = 0.000) | False (rho = 0.664, p = 0.007) | -0.01 ± 0.37 | [-0.74, 0.72] | 0.38 | True (W = 0.939, p = 0.368) | True (rho = -0.311, p = 0.260) | -0.09 ± 0.48 | [-1.03, 0.85] | 0.48 | True (W = 0.916, p = 0.167) | False (rho = -0.600, p = 0.018) |
| ECG Neurokit & Elgendi; PPG Langevin & Elgendi | easy | 0.05 ± 0.09 | [-0.12, 0.23] | 0.09 | False (W = 0.621, p = 0.000) | False (rho = 0.829, p = 0.000) | 0.12 ± 0.22 | [-0.32, 0.56] | 0.22 | True (W = 0.957, p = 0.641) | True (rho = 0.132, p = 0.639) | 0.03 ± 0.34 | [-0.64, 0.71] | 0.34 | True (W = 0.979, p = 0.964) | True (rho = -0.507, p = 0.054) |
| ECG Neurokit & Elgendi; PPG Langevin & Elgendi | hard | 0.05 ± 0.08 | [-0.11, 0.20] | 0.08 | False (W = 0.661, p = 0.000) | False (rho = 0.682, p = 0.005) | 0.12 ± 0.32 | [-0.51, 0.74] | 0.32 | True (W = 0.961, p = 0.714) | True (rho = -0.100, p = 0.723) | 0.06 ± 0.44 | [-0.80, 0.92] | 0.45 | True (W = 0.953, p = 0.575) | True (rho = -0.236, p = 0.398) |
| ECG Neurokit & Nabian; PPG Elgendi & Elgendi | easy | 0.01 ± 0.02 | [-0.03, 0.04] | 0.02 | True (W = 0.929, p = 0.267) | True (rho = -0.104, p = 0.713) | -0.30 ± 0.30 | [-0.90, 0.29] | 0.32 | True (W = 0.967, p = 0.818) | True (rho = -0.368, p = 0.177) | -0.48 ± 0.42 | [-1.30, 0.34] | 0.46 | True (W = 0.957, p = 0.645) | True (rho = -0.132, p = 0.639) |
| ECG Neurokit & Nabian; PPG Elgendi & Elgendi | hard | 0.01 ± 0.01 | [-0.02, 0.03] | 0.01 | True (W = 0.966, p = 0.802) | True (rho = 0.129, p = 0.648) | -0.23 ± 0.27 | [-0.77, 0.31] | 0.3 | True (W = 0.920, p = 0.190) | True (rho = -0.086, p = 0.761) | -0.42 ± 0.41 | [-1.24, 0.39] | 0.45 | True (W = 0.977, p = 0.942) | True (rho = -0.246, p = 0.376) |
| ECG Neurokit & Nabian; PPG Langevin & Elgendi | easy | 0.00 ± 0.02 | [-0.03, 0.03] | 0.02 | True (W = 0.937, p = 0.346) | True (rho = -0.093, p = 0.742) | -0.21 ± 0.25 | [-0.69, 0.27] | 0.27 | True (W = 0.979, p = 0.961) | True (rho = -0.443, p = 0.098) | -0.36 ± 0.38 | [-1.10, 0.37] | 0.42 | True (W = 0.962, p = 0.730) | True (rho = 0.061, p = 0.830) |
| ECG Neurokit & Nabian; PPG Langevin & Elgendi | hard | 0.00 ± 0.01 | [-0.02, 0.03] | 0.01 | True (W = 0.954, p = 0.590) | True (rho = 0.143, p = 0.612) | -0.16 ± 0.27 | [-0.69, 0.36] | 0.29 | True (W = 0.919, p = 0.189) | True (rho = -0.121, p = 0.666) | -0.31 ± 0.41 | [-1.11, 0.49] | 0.46 | True (W = 0.954, p = 0.585) | True (rho = -0.179, p = 0.524) |
| ECG Neurokit & Neurokit; PPG Elgendi & Elgendi | easy | 0.00 ± 0.02 | [-0.03, 0.04] | 0.02 | True (W = 0.914, p = 0.154) | True (rho = -0.118, p = 0.676) | -0.35 ± 0.26 | [-0.87, 0.17] | 0.28 | True (W = 0.942, p = 0.406) | True (rho = -0.436, p = 0.104) | -0.57 ± 0.34 | [-1.24, 0.10] | 0.38 | True (W = 0.961, p = 0.708) | True (rho = -0.096, p = 0.732) |
| ECG Neurokit & Neurokit; PPG Elgendi & Elgendi | hard | 0.00 ± 0.01 | [-0.02, 0.03] | 0.01 | True (W = 0.973, p = 0.900) | True (rho = 0.175, p = 0.533) | -0.24 ± 0.29 | [-0.81, 0.33] | 0.32 | True (W = 0.984, p = 0.991) | True (rho = -0.050, p = 0.860) | -0.49 ± 0.40 | [-1.28, 0.31] | 0.45 | True (W = 0.962, p = 0.722) | True (rho = -0.132, p = 0.639) |
| ECG Neurokit & Neurokit; PPG Langevin & Elgendi | easy | 0.00 ± 0.02 | [-0.03, 0.03] | 0.02 | True (W = 0.926, p = 0.236) | True (rho = -0.068, p = 0.810) | -0.26 ± 0.20 | [-0.66, 0.14] | 0.23 | True (W = 0.947, p = 0.473) | True (rho = -0.425, p = 0.114) | -0.45 ± 0.30 | [-1.05, 0.14] | 0.35 | True (W = 0.966, p = 0.792) | True (rho = 0.107, p = 0.704) |
| ECG Neurokit & Neurokit; PPG Langevin & Elgendi | hard | 0.00 ± 0.01 | [-0.03, 0.03] | 0.02 | True (W = 0.981, p = 0.977) | True (rho = 0.093, p = 0.742) | -0.18 ± 0.27 | [-0.72, 0.36] | 0.3 | True (W = 0.974, p = 0.918) | True (rho = 0.100, p = 0.723) | -0.38 ± 0.39 | [-1.14, 0.37] | 0.44 | True (W = 0.984, p = 0.990) | True (rho = -0.104, p = 0.713) |

*Note*. The BAr results and corresponding values for all CVA parameters of interest across tested pipelines. The pipeline annotation consists of “ECG data cleaning method & peak detection method; PPG data cleaning method & peak detection method”. The pipeline configuration used for creating the Bland-Altman plots is marked with an asterisk.

Table 9
*BAr Results for CVA Parameters Across Processing Pipelines with Original ECG Sampling Rate*

| Processing pipeline | Workload condition | HR (bpm) | | | | | | SD RR/PP interval (ms) | | | | | | RMSSD (ms) | | | | | |
| --- | --- | --- | --- | --- | --- | --- | --- | --- | --- | --- | --- | --- | --- | --- | --- | --- | --- | --- | --- |
|  |  | Mean ± SD | 95% CI | BAr | Within boundaries (%) | normality | homoscedasticity | Mean ± SD | 95% CI | BAr | Within boundaries (%) | normality | homoscedasticity | Mean ± SD | 95% CI | BAr | Within boundaries (%) | normality | homoscedasticity |
| ECG BioSPPY & Elgendi; PPG Elgendi & Elgendi | easy | 10.68 ± 20.50 | [-29.49, 50.85] | 0.53 | 66.67 | False (W = 0.606, p = 0.000) | False (rho = 0.707, p = 0.003) | -8.14 ± 70.32 | [-145.96, 129.69] | 1.42 | 73.33 | True (W = 0.949, p = 0.508) | True (rho = 0.121, p = 0.666) | -38.98 ± 95.59 | [-226.33, 148.37] | 1.79 | 46.67 | True (W = 0.919, p = 0.188) | False (rho = 0.564, p = 0.028) |
| ECG BioSPPY & Elgendi; PPG Elgendi & Elgendi | hard | 7.37 ± 21.10 | [-34.00, 48.73] | 0.55 | 80 | False (W = 0.401, p = 0.000) | False (rho = 0.568, p = 0.027) | -19.09 ± 58.01 | [-132.78, 94.61] | 1.45 | 60 | True (W = 0.904, p = 0.109) | True (rho = 0.271, p = 0.328) | -40.00 ± 81.72 | [-200.17, 120.18] | 1.8 | 60 | True (W = 0.969, p = 0.847) | False (rho = 0.525, p = 0.044) |
| ECG BioSPPY & Elgendi; PPG Langevin & Elgendi | easy | 9.09 ± 17.73 | [-25.65, 43.84] | 0.46 | 66.67 | False (W = 0.639, p = 0.000) | False (rho = 0.707, p = 0.003) | 17.73 ± 66.21 | [-112.05, 147.50] | 1.4 | 60 | True (W = 0.945, p = 0.443) | False (rho = 0.657, p = 0.008) | -1.59 ± 80.12 | [-158.64, 155.45] | 1.68 | 73.33 | True (W = 0.923, p = 0.215) | True (rho = 0.161, p = 0.567) |
| ECG BioSPPY & Elgendi; PPG Langevin & Elgendi | hard | 7.06 ± 21.11 | [-34.32, 48.43] | 0.55 | 80 | False (W = 0.390, p = 0.000) | True (rho = 0.511, p = 0.052) | -7.97 ± 54.29 | [-114.38, 98.43] | 1.44 | 86.67 | True (W = 0.908, p = 0.128) | True (rho = 0.064, p = 0.820) | -21.40 ± 78.22 | [-174.71, 131.91] | 1.95 | 60 | True (W = 0.939, p = 0.375) | False (rho = 0.671, p = 0.006) |
| ECG BioSPPY & Nabian; PPG Elgendi & Elgendi | easy | 0.74 ± 3.15 | [-5.44, 6.93] | 0.09 | 86.67 | False (W = 0.866, p = 0.030) | True (rho = 0.307, p = 0.265) | -48.35 ± 43.38 | [-133.37, 36.68] | 1.1 | 60 | True (W = 0.887, p = 0.059) | True (rho = 0.000, p = 1.000) | -77.68 ± 71.63 | [-218.07, 62.71] | 1.65 | 46.67 | True (W = 0.953, p = 0.566) | True (rho = 0.357, p = 0.191) |
| ECG BioSPPY & Nabian; PPG Elgendi & Elgendi | hard | 0.77 ± 2.40 | [-3.94, 5.47] | 0.07 | 93.33 | True (W = 0.961, p = 0.708) | True (rho = 0.461, p = 0.084) | -30.53 ± 49.45 | [-127.46, 66.41] | 1.33 | 60 | True (W = 0.981, p = 0.978) | True (rho = 0.382, p = 0.160) | -57.48 ± 81.71 | [-217.63, 102.68] | 2.0 | 33.33 | True (W = 0.956, p = 0.623) | False (rho = 0.614, p = 0.015) |
| ECG BioSPPY & Nabian; PPG Langevin & Elgendi | easy | 0.28 ± 2.98 | [-5.56, 6.11] | 0.08 | 86.67 | True (W = 0.887, p = 0.061) | True (rho = 0.296, p = 0.283) | -30.23 ± 30.04 | [-89.11, 28.65] | 0.86 | 80 | True (W = 0.912, p = 0.147) | True (rho = -0.329, p = 0.232) | -47.59 ± 53.36 | [-152.17, 56.99] | 1.49 | 66.67 | True (W = 0.943, p = 0.423) | True (rho = 0.214, p = 0.443) |
| ECG BioSPPY & Nabian; PPG Langevin & Elgendi | hard | 0.50 ± 2.47 | [-4.35, 5.34] | 0.07 | 93.33 | True (W = 0.958, p = 0.651) | True (rho = 0.218, p = 0.435) | -18.57 ± 45.04 | [-106.85, 69.72] | 1.29 | 80 | True (W = 0.979, p = 0.966) | False (rho = 0.586, p = 0.022) | -35.80 ± 76.68 | [-186.10, 114.49] | 2.11 | 40 | True (W = 0.960, p = 0.688) | False (rho = 0.700, p = 0.004) |
| ECG BioSPPY & Neurokit; PPG Elgendi & Elgendi | easy | 0.61 ± 3.16 | [-5.59, 6.80] | 0.09 | 86.67 | False (W = 0.860, p = 0.024) | True (rho = 0.121, p = 0.666) | -44.67 ± 48.77 | [-140.25, 50.91] | 1.21 | 60 | True (W = 0.964, p = 0.767) | True (rho = 0.075, p = 0.791) | -76.06 ± 74.99 | [-223.04, 70.93] | 1.71 | 46.67 | True (W = 0.963, p = 0.742) | True (rho = 0.443, p = 0.098) |
| ECG BioSPPY & Neurokit; PPG Elgendi & Elgendi | hard | 1.22 ± 2.43 | [-3.55, 5.98] | 0.07 | 86.67 | True (W = 0.943, p = 0.427) | True (rho = 0.457, p = 0.087) | -32.27 ± 44.61 | [-119.71, 55.16] | 1.21 | 60 | True (W = 0.949, p = 0.501) | True (rho = 0.114, p = 0.685) | -60.96 ± 70.73 | [-199.60, 77.68] | 1.77 | 53.33 | True (W = 0.969, p = 0.843) | True (rho = 0.486, p = 0.066) |
| ECG BioSPPY & Neurokit; PPG Langevin & Elgendi | easy | 0.14 ± 2.96 | [-5.66, 5.94] | 0.08 | 86.67 | False (W = 0.853, p = 0.019) | True (rho = 0.318, p = 0.248) | -26.70 ± 35.18 | [-95.65, 42.25] | 0.98 | 80 | True (W = 0.982, p = 0.979) | True (rho = -0.064, p = 0.820) | -46.13 ± 57.41 | [-158.66, 66.41] | 1.59 | 66.67 | True (W = 0.960, p = 0.690) | True (rho = 0.486, p = 0.066) |
| ECG BioSPPY & Neurokit; PPG Langevin & Elgendi | hard | 0.88 ± 2.33 | [-3.69, 5.45] | 0.06 | 93.33 | True (W = 0.955, p = 0.604) | True (rho = 0.268, p = 0.334) | -20.74 ± 40.07 | [-99.27, 57.80] | 1.17 | 86.67 | True (W = 0.952, p = 0.555) | True (rho = 0.118, p = 0.676) | -40.93 ± 65.74 | [-169.78, 87.92] | 1.87 | 53.33 | True (W = 0.981, p = 0.976) | False (rho = 0.529, p = 0.043) |
| ECG Elgendi & Elgendi; PPG Elgendi & Elgendi | easy | 2.02 ± 3.55 | [-4.93, 8.97] | 0.1 | 80 | False (W = 0.825, p = 0.008) | True (rho = -0.175, p = 0.533) | -30.98 ± 76.45 | [-180.82, 118.86] | 1.76 | 60 | False (W = 0.828, p = 0.009) | True (rho = 0.389, p = 0.152) | -55.38 ± 119.08 | [-288.78, 178.02] | 2.38 | 33.33 | False (W = 0.824, p = 0.008) | False (rho = 0.643, p = 0.010) |
| ECG Elgendi & Elgendi; PPG Elgendi & Elgendi | hard | 2.12 ± 3.42 | [-4.59, 8.83] | 0.09 | 85.71 | False (W = 0.824, p = 0.010) | True (rho = 0.490, p = 0.075) | -38.86 ± 35.45 | [-108.35, 30.62] | 1.02 | 71.43 | True (W = 0.934, p = 0.344) | False (rho = 0.587, p = 0.027) | -73.35 ± 54.05 | [-179.28, 32.58] | 1.43 | 42.86 | True (W = 0.971, p = 0.886) | False (rho = 0.776, p = 0.001) |
| ECG Elgendi & Elgendi; PPG Langevin & Elgendi | easy | 1.51 ± 3.52 | [-5.39, 8.40] | 0.1 | 80 | False (W = 0.829, p = 0.009) | True (rho = -0.057, p = 0.840) | -12.71 ± 66.26 | [-142.58, 117.16] | 1.68 | 80 | False (W = 0.739, p = 0.001) | True (rho = 0.436, p = 0.104) | -25.38 ± 104.09 | [-229.40, 178.64] | 2.46 | 60 | False (W = 0.743, p = 0.001) | False (rho = 0.764, p = 0.001) |
| ECG Elgendi & Elgendi; PPG Langevin & Elgendi | hard | 1.73 ± 2.93 | [-4.01, 7.46] | 0.08 | 85.71 | False (W = 0.866, p = 0.036) | True (rho = 0.415, p = 0.140) | -27.66 ± 32.93 | [-92.21, 36.89] | 1.02 | 92.86 | True (W = 0.904, p = 0.131) | False (rho = 0.565, p = 0.035) | -52.35 ± 52.28 | [-154.83, 50.12] | 1.6 | 71.43 | True (W = 0.931, p = 0.315) | False (rho = 0.679, p = 0.008) |
| ECG Elgendi & Nabian; PPG Elgendi & Elgendi | easy | 0.93 ± 3.05 | [-5.05, 6.91] | 0.08 | 86.67 | False (W = 0.859, p = 0.023) | True (rho = 0.196, p = 0.483) | -50.59 ± 44.16 | [-137.13, 35.96] | 1.14 | 60 | True (W = 0.971, p = 0.877) | True (rho = 0.439, p = 0.101) | -83.72 ± 71.54 | [-223.94, 56.49] | 1.7 | 33.33 | True (W = 0.950, p = 0.525) | False (rho = 0.746, p = 0.001) |
| ECG Elgendi & Nabian; PPG Elgendi & Elgendi | hard | 1.79 ± 3.18 | [-4.45, 8.02] | 0.09 | 86.67 | False (W = 0.862, p = 0.026) | True (rho = 0.393, p = 0.147) | -38.81 ± 40.05 | [-117.31, 39.69] | 1.14 | 60 | True (W = 0.967, p = 0.814) | False (rho = 0.621, p = 0.013) | -75.39 ± 57.06 | [-187.23, 36.45] | 1.57 | 46.67 | True (W = 0.977, p = 0.942) | False (rho = 0.682, p = 0.005) |
| ECG Elgendi & Nabian; PPG Langevin & Elgendi * | easy | 0.42 ± 2.85 | [-5.17, 6.00] | 0.08 | 86.67 | False (W = 0.858, p = 0.023) | True (rho = 0.014, p = 0.960) | -32.39 ± 31.32 | [-93.78, 29.00] | 0.91 | 80 | True (W = 0.989, p = 0.999) | True (rho = 0.171, p = 0.541) | -54.02 ± 54.13 | [-160.11, 52.07] | 1.58 | 66.67 | True (W = 0.918, p = 0.179) | False (rho = 0.754, p = 0.001) |
| ECG Elgendi & Nabian; PPG Langevin & Elgendi * | hard | 1.44 ± 2.97 | [-4.39, 7.27] | 0.08 | 86.67 | True (W = 0.907, p = 0.122) | True (rho = 0.300, p = 0.277) | -27.12 ± 36.57 | [-98.81, 44.56] | 1.12 | 86.67 | True (W = 0.959, p = 0.677) | True (rho = 0.468, p = 0.079) | -55.07 ± 54.14 | [-161.18, 51.03] | 1.72 | 53.33 | True (W = 0.968, p = 0.831) | False (rho = 0.657, p = 0.008) |
| ECG Elgendi & Neurokit; PPG Elgendi & Elgendi | easy | 0.82 ± 3.07 | [-5.20, 6.85] | 0.08 | 86.67 | False (W = 0.854, p = 0.020) | True (rho = 0.136, p = 0.630) | -48.99 ± 44.09 | [-135.41, 37.44] | 1.13 | 66.67 | True (W = 0.970, p = 0.856) | True (rho = 0.304, p = 0.271) | -78.59 ± 73.37 | [-222.38, 65.21] | 1.69 | 40 | True (W = 0.968, p = 0.827) | False (rho = 0.671, p = 0.006) |
| ECG Elgendi & Neurokit; PPG Elgendi & Elgendi | hard | 1.71 ± 3.39 | [-4.93, 8.36] | 0.09 | 86.67 | False (W = 0.825, p = 0.008) | False (rho = 0.518, p = 0.048) | -37.04 ± 41.60 | [-118.58, 44.50] | 1.17 | 66.67 | True (W = 0.982, p = 0.980) | False (rho = 0.529, p = 0.043) | -65.15 ± 64.15 | [-190.89, 60.60] | 1.65 | 60 | True (W = 0.987, p = 0.997) | False (rho = 0.664, p = 0.007) |
| ECG Elgendi & Neurokit; PPG Langevin & Elgendi | easy | 0.32 ± 2.89 | [-5.34, 5.99] | 0.08 | 86.67 | False (W = 0.857, p = 0.022) | True (rho = 0.175, p = 0.533) | -30.63 ± 30.80 | [-90.99, 29.73] | 0.88 | 80 | True (W = 0.974, p = 0.914) | True (rho = 0.136, p = 0.630) | -48.31 ± 55.33 | [-156.76, 60.14] | 1.55 | 66.67 | True (W = 0.942, p = 0.404) | False (rho = 0.675, p = 0.006) |
| ECG Elgendi & Neurokit; PPG Langevin & Elgendi | hard | 1.36 ± 3.14 | [-4.80, 7.51] | 0.08 | 86.67 | False (W = 0.868, p = 0.032) | True (rho = 0.339, p = 0.216) | -25.06 ± 38.82 | [-101.15, 51.03] | 1.17 | 86.67 | True (W = 0.968, p = 0.832) | True (rho = 0.507, p = 0.054) | -44.23 ± 62.57 | [-166.86, 78.41] | 1.83 | 66.67 | True (W = 0.969, p = 0.846) | False (rho = 0.704, p = 0.003) |
| ECG Langevin & Elgendi; PPG Elgendi & Elgendi | easy | 2.23 ± 4.18 | [-5.96, 10.43] | 0.11 | 80 | False (W = 0.749, p = 0.001) | True (rho = 0.421, p = 0.118) | -1.19 ± 64.36 | [-127.34, 124.95] | 1.25 | 73.33 | True (W = 0.966, p = 0.797) | True (rho = 0.086, p = 0.761) | -10.45 ± 92.91 | [-192.55, 171.64] | 1.53 | 40 | True (W = 0.962, p = 0.727) | True (rho = 0.025, p = 0.930) |
| ECG Langevin & Elgendi; PPG Elgendi & Elgendi | hard | 1.82 ± 3.56 | [-5.16, 8.80] | 0.1 | 85.71 | True (W = 0.900, p = 0.114) | False (rho = 0.547, p = 0.043) | -18.77 ± 49.76 | [-116.30, 78.76] | 1.22 | 78.57 | True (W = 0.915, p = 0.188) | True (rho = 0.011, p = 0.970) | -22.74 ± 87.27 | [-193.78, 148.30] | 1.71 | 50 | True (W = 0.964, p = 0.783) | True (rho = 0.275, p = 0.342) |
| ECG Langevin & Elgendi; PPG Langevin & Elgendi | easy | 1.59 ± 3.76 | [-5.78, 8.96] | 0.1 | 80 | False (W = 0.782, p = 0.002) | True (rho = 0.475, p = 0.074) | 16.72 ± 48.45 | [-78.24, 111.68] | 1.03 | 86.67 | True (W = 0.966, p = 0.787) | False (rho = 0.518, p = 0.048) | 20.64 ± 71.42 | [-119.34, 160.63] | 1.34 | 66.67 | True (W = 0.938, p = 0.355) | True (rho = 0.325, p = 0.237) |
| ECG Langevin & Elgendi; PPG Langevin & Elgendi | hard | 1.30 ± 3.14 | [-4.86, 7.46] | 0.08 | 85.71 | True (W = 0.955, p = 0.643) | True (rho = 0.358, p = 0.208) | -3.40 ± 49.34 | [-100.11, 93.32] | 1.28 | 92.86 | True (W = 0.912, p = 0.170) | True (rho = -0.002, p = 0.994) | 1.72 ± 89.05 | [-172.82, 176.26] | 1.94 | 50 | True (W = 0.970, p = 0.881) | True (rho = 0.393, p = 0.164) |
| ECG Langevin & Nabian; PPG Elgendi & Elgendi | easy | 0.94 ± 3.05 | [-5.05, 6.92] | 0.08 | 86.67 | False (W = 0.871, p = 0.035) | True (rho = -0.036, p = 0.899) | -43.00 ± 49.67 | [-140.35, 54.35] | 1.22 | 60 | True (W = 0.969, p = 0.844) | True (rho = -0.143, p = 0.612) | -73.15 ± 78.56 | [-227.12, 80.82] | 1.76 | 46.67 | True (W = 0.963, p = 0.751) | True (rho = 0.121, p = 0.666) |
| ECG Langevin & Nabian; PPG Elgendi & Elgendi | hard | 1.42 ± 3.14 | [-4.75, 7.58] | 0.09 | 86.67 | False (W = 0.868, p = 0.031) | True (rho = 0.307, p = 0.265) | -35.00 ± 45.44 | [-124.07, 54.07] | 1.26 | 66.67 | True (W = 0.978, p = 0.952) | True (rho = 0.379, p = 0.164) | -62.78 ± 74.25 | [-208.31, 82.74] | 1.87 | 53.33 | True (W = 0.967, p = 0.818) | False (rho = 0.589, p = 0.021) |
| ECG Langevin & Nabian; PPG Langevin & Elgendi | easy | 0.41 ± 2.89 | [-5.25, 6.07] | 0.08 | 86.67 | False (W = 0.869, p = 0.032) | True (rho = -0.021, p = 0.940) | -23.18 ± 34.50 | [-90.79, 44.44] | 0.93 | 86.67 | True (W = 0.974, p = 0.918) | True (rho = -0.104, p = 0.713) | -40.42 ± 57.35 | [-152.83, 71.99] | 1.52 | 66.67 | True (W = 0.938, p = 0.356) | True (rho = 0.286, p = 0.302) |
| ECG Langevin & Nabian; PPG Langevin & Elgendi | hard | 1.12 ± 2.89 | [-4.53, 6.78] | 0.08 | 86.67 | True (W = 0.920, p = 0.192) | True (rho = 0.211, p = 0.451) | -17.03 ± 43.27 | [-101.84, 67.78] | 1.23 | 86.67 | True (W = 0.979, p = 0.960) | True (rho = 0.464, p = 0.081) | -35.33 ± 69.23 | [-171.02, 100.37] | 1.9 | 53.33 | True (W = 0.968, p = 0.829) | True (rho = 0.511, p = 0.052) |
| ECG Langevin & Neurokit; PPG Elgendi & Elgendi | easy | -1.81 ± 7.15 | [-15.83, 12.21] | 0.2 | 78.57 | False (W = 0.836, p = 0.015) | True (rho = 0.015, p = 0.958) | 160.02 ± 571.45 | [-960.02, 1280.06] | 6.13 | 50 | False (W = 0.423, p = 0.000) | False (rho = 0.666, p = 0.009) | 211.91 ± 831.99 | [-1418.79, 1842.62] | 7.02 | 35.71 | False (W = 0.416, p = 0.000) | True (rho = 0.490, p = 0.075) |
| ECG Langevin & Neurokit; PPG Elgendi & Elgendi | hard | -2.22 ± 6.21 | [-14.39, 9.95] | 0.17 | 73.33 | False (W = 0.832, p = 0.010) | True (rho = 0.200, p = 0.475) | 58.38 ± 220.38 | [-373.56, 490.31] | 3.69 | 53.33 | False (W = 0.668, p = 0.000) | False (rho = 0.604, p = 0.017) | 81.14 ± 333.70 | [-572.92, 735.20] | 4.38 | 40 | False (W = 0.686, p = 0.000) | False (rho = 0.675, p = 0.006) |
| ECG Langevin & Neurokit; PPG Langevin & Elgendi | easy | -1.34 ± 4.48 | [-10.11, 7.43] | 0.12 | 78.57 | True (W = 0.973, p = 0.914) | True (rho = 0.222, p = 0.446) | 109.76 ± 331.81 | [-540.59, 760.12] | 4.66 | 64.29 | False (W = 0.491, p = 0.000) | False (rho = 0.824, p = 0.000) | 153.10 ± 479.51 | [-786.74, 1092.95] | 5.47 | 50 | False (W = 0.504, p = 0.000) | False (rho = 0.670, p = 0.009) |
| ECG Langevin & Neurokit; PPG Langevin & Elgendi | hard | -2.38 ± 5.99 | [-14.12, 9.37] | 0.17 | 73.33 | False (W = 0.856, p = 0.021) | True (rho = 0.157, p = 0.576) | 69.00 ± 217.35 | [-357.01, 495.00] | 3.81 | 66.67 | False (W = 0.644, p = 0.000) | False (rho = 0.789, p = 0.000) | 102.43 ± 329.38 | [-543.15, 748.01] | 4.61 | 46.67 | False (W = 0.661, p = 0.000) | False (rho = 0.771, p = 0.001) |
| ECG Neurokit & Elgendi; PPG Elgendi & Elgendi | easy | 11.78 ± 21.71 | [-30.77, 54.34] | 0.56 | 60 | False (W = 0.588, p = 0.000) | False (rho = 0.800, p = 0.000) | 6.60 ± 66.95 | [-124.63, 137.82] | 1.26 | 66.67 | True (W = 0.930, p = 0.276) | True (rho = -0.068, p = 0.810) | -15.20 ± 89.12 | [-189.88, 159.49] | 1.5 | 40 | False (W = 0.879, p = 0.047) | True (rho = 0.321, p = 0.243) |
| ECG Neurokit & Elgendi; PPG Elgendi & Elgendi | hard | 10.14 ± 20.74 | [-30.51, 50.78] | 0.53 | 60 | False (W = 0.543, p = 0.000) | False (rho = 0.664, p = 0.007) | 15.39 ± 73.89 | [-129.43, 160.22] | 1.51 | 53.33 | True (W = 0.916, p = 0.170) | True (rho = 0.318, p = 0.248) | -0.60 ± 98.96 | [-194.57, 193.36] | 1.78 | 33.33 | True (W = 0.920, p = 0.195) | True (rho = 0.279, p = 0.315) |
| ECG Neurokit & Elgendi; PPG Langevin & Elgendi | easy | 11.35 ± 20.12 | [-28.09, 50.79] | 0.52 | 53.33 | False (W = 0.591, p = 0.000) | False (rho = 0.836, p = 0.000) | 35.27 ± 50.46 | [-63.63, 134.17] | 0.97 | 60 | True (W = 0.964, p = 0.764) | True (rho = 0.489, p = 0.064) | 23.10 ± 70.61 | [-115.30, 161.49] | 1.31 | 80 | True (W = 0.907, p = 0.121) | True (rho = 0.086, p = 0.761) |
| ECG Neurokit & Elgendi; PPG Langevin & Elgendi | hard | 10.13 ± 20.80 | [-30.64, 50.91] | 0.53 | 60 | False (W = 0.544, p = 0.000) | False (rho = 0.682, p = 0.005) | 37.33 ± 63.90 | [-87.91, 162.56] | 1.3 | 60 | True (W = 0.965, p = 0.783) | False (rho = 0.596, p = 0.019) | 28.63 ± 87.97 | [-143.79, 201.04] | 1.67 | 40 | True (W = 0.950, p = 0.527) | True (rho = 0.439, p = 0.101) |
| ECG Neurokit & Nabian; PPG Elgendi & Elgendi | easy | 1.01 ± 3.06 | [-4.99, 7.01] | 0.08 | 86.67 | False (W = 0.881, p = 0.050) | True (rho = 0.107, p = 0.704) | -43.94 ± 51.19 | [-144.28, 56.39] | 1.27 | 53.33 | True (W = 0.976, p = 0.935) | True (rho = 0.289, p = 0.296) | -70.81 ± 87.10 | [-241.53, 99.91] | 1.92 | 40 | True (W = 0.966, p = 0.790) | False (rho = 0.746, p = 0.001) |
| ECG Neurokit & Nabian; PPG Elgendi & Elgendi | hard | 0.95 ± 2.22 | [-3.39, 5.30] | 0.06 | 93.33 | True (W = 0.958, p = 0.653) | True (rho = 0.246, p = 0.376) | -32.27 ± 44.10 | [-118.71, 54.17] | 1.2 | 60 | True (W = 0.936, p = 0.335) | True (rho = 0.321, p = 0.243) | -59.25 ± 72.93 | [-202.19, 83.69] | 1.8 | 60 | True (W = 0.968, p = 0.829) | False (rho = 0.532, p = 0.041) |
| ECG Neurokit & Nabian; PPG Langevin & Elgendi | easy | 0.50 ± 2.87 | [-5.12, 6.13] | 0.08 | 86.67 | False (W = 0.880, p = 0.048) | True (rho = -0.032, p = 0.909) | -25.59 ± 39.99 | [-103.97, 52.79] | 1.1 | 73.33 | True (W = 0.943, p = 0.427) | True (rho = 0.250, p = 0.369) | -40.71 ± 71.92 | [-181.68, 100.27] | 1.91 | 60 | True (W = 0.901, p = 0.098) | False (rho = 0.761, p = 0.001) |
| ECG Neurokit & Nabian; PPG Langevin & Elgendi | hard | 0.68 ± 2.30 | [-3.83, 5.19] | 0.06 | 93.33 | True (W = 0.943, p = 0.425) | True (rho = 0.250, p = 0.369) | -20.98 ± 39.12 | [-97.66, 55.71] | 1.14 | 86.67 | True (W = 0.937, p = 0.348) | True (rho = 0.350, p = 0.201) | -39.21 ± 65.07 | [-166.74, 88.31] | 1.83 | 60 | True (W = 0.957, p = 0.645) | False (rho = 0.543, p = 0.037) |
| ECG Neurokit & Neurokit; PPG Elgendi & Elgendi | easy | 0.93 ± 3.06 | [-5.07, 6.92] | 0.08 | 86.67 | False (W = 0.862, p = 0.026) | True (rho = 0.061, p = 0.830) | -53.23 ± 40.88 | [-133.35, 26.89] | 1.07 | 60 | True (W = 0.946, p = 0.468) | True (rho = 0.293, p = 0.289) | -88.49 ± 65.71 | [-217.29, 40.31] | 1.61 | 33.33 | True (W = 0.982, p = 0.982) | False (rho = 0.679, p = 0.005) |
| ECG Neurokit & Neurokit; PPG Elgendi & Elgendi | hard | 0.77 ± 2.38 | [-3.90, 5.43] | 0.07 | 93.33 | True (W = 0.969, p = 0.841) | True (rho = 0.321, p = 0.243) | -30.10 ± 52.00 | [-132.03, 71.82] | 1.39 | 60 | True (W = 0.972, p = 0.882) | True (rho = 0.482, p = 0.069) | -65.57 ± 69.82 | [-202.42, 71.27] | 1.8 | 40 | True (W = 0.985, p = 0.992) | True (rho = 0.418, p = 0.121) |
| ECG Neurokit & Neurokit; PPG Langevin & Elgendi | easy | 0.43 ± 2.86 | [-5.18, 6.03] | 0.08 | 100 | False (W = 0.867, p = 0.031) | True (rho = 0.046, p = 0.869) | -34.71 ± 27.85 | [-89.30, 19.88] | 0.82 | 80 | True (W = 0.962, p = 0.726) | True (rho = 0.064, p = 0.820) | -58.05 ± 48.29 | [-152.70, 36.61] | 1.46 | 60 | True (W = 0.947, p = 0.484) | False (rho = 0.657, p = 0.008) |
| ECG Neurokit & Neurokit; PPG Langevin & Elgendi | hard | 0.49 ± 2.47 | [-4.35, 5.32] | 0.07 | 100 | True (W = 0.967, p = 0.808) | True (rho = 0.221, p = 0.428) | -19.24 ± 45.74 | [-108.90, 70.42] | 1.32 | 80 | True (W = 0.956, p = 0.621) | True (rho = 0.389, p = 0.152) | -45.84 ± 62.80 | [-168.92, 77.25] | 1.86 | 53.33 | True (W = 0.973, p = 0.902) | True (rho = 0.429, p = 0.111) |

*Note*. The BAr results and corresponding values for all CVA parameters of interest across tested pipelines. The pipeline annotation consists of “ECG data cleaning method & peak detection method; PPG data cleaning method & peak detection method”. The pipeline configuration used for creating the Bland-Altman plots is marked with an asterisk.
